# Genetic Association of Juvenile Idiopathic Arthritis With Adult Rheumatic Disease

**DOI:** 10.1001/jamanetworkopen.2024.51341

**Published:** 2024-12-27

**Authors:** Jingxian Fan, Jian Hao, Yuqiao Fu, Xiaoyang Liu, Hui-Qi Qu, Joseph T. Glessner, Dandan Ji, Wei Liu, Gang Zheng, Zhiyong Ding, Shuzhong Cui, Qianghua Xia, Hakon Hakonarson, Wei Wei, Jin Li

**Affiliations:** 1 Department of Cell Biology, The Province and Ministry Cosponsored Collaborative Innovation Center for Medical Epigenetics, Key Laboratory of Immune Microenvironment and Disease (Ministry of Education), Tianjin Key Laboratory of Medical Epigenetics, Tianjin Institute of Immunology, School of Basic Medical Sciences, Tianjin Medical University, Tianjin, China; 2 Department of Rheumatology and Immunology, Tianjin Clinical Research Center for Rheumatic and Immune Diseases, Tianjin Medical University General Hospital, Tianjin, China; 3 Department of Bioinformatics, Tianjin Medical University, Tianjin, China; 4 Center for Applied Genomics, Children’s Hospital of Philadelphia, Philadelphia, Pennsylvania; 5 Division of Human Genetics, Children’s Hospital of Philadelphia, Philadelphia, Pennsylvania; 6 Department of Pediatrics, Perelman School of Medicine, University of Pennsylvania, Philadelphia; 7 Tianjin Children’s Hospital, Tianjin University, Tianjin, China; 8 Tianjin Key Laboratory of Birth Defects for Prevention and Treatment, Tianjin, China; 9 National Supercomputer Center in Tianjin, Tianjin, China; 10 Mills Institute for Personalized Cancer Care, Fynn Biotechnologies Ltd, Jinan, China; 11 Affiliated Cancer Hospital, Institute of Guangzhou Medical University, Guangzhou, China

## Abstract

**Importance:**

Patients with juvenile idiopathic arthritis (JIA) may develop adult rheumatic diseases later in life, and prolonged or recurrent disease activity is often associated with substantial disability; therefore, it is important to identify patients with JIA at high risk of developing adult rheumatic diseases and provide specialized attention and preventive care to them.

**Objective:**

To elucidate the full extent of the genetic association of JIA with adult rheumatic diseases, to improve treatment strategies and patient outcomes for patients at high risk of developing long-term rheumatic diseases.

**Design, Setting, and Participants:**

In this genetic association study of 4 disease genome-wide association study (GWAS) cohorts from 2013 to 2024 (JIA, rheumatoid arthritis [RA], systemic lupus erythematosus [SLE], and systemic sclerosis [SSc]), patients in the JIA cohort were recruited from the US, Australia, and Norway (with a UK cohort included in the meta-analyzed cohort), while patients in the other 3 cohorts were recruited from US and Western European countries. All analyses were conducted between September 2023 and April 2024.

**Exposures:**

Genetic associations.

**Main Outcomes and Measures:**

Genetic correlations and shared genomic loci between JIA and adult rheumatic diseases. Genetic correlation analyses and cross-trait meta-analysis were conducted on the JIA cohort and the summary statistics of the GWASs from adult rheumatic diseases (RA, SLE, and SSc). Mendelian randomization analyses were also conducted.

**Results:**

This study included 33 207 patients across the 4 cohorts, with 4550 patients in the meta-analyzed JIA cohort (JIA cohort: 1485 patients with arthritis onset before 16 years; 1017 female [68.5%]; 10 352 controls; UK cohort: 3305 patients with JIA; 9196 controls), 143 61 patients in the RA cohort, 5201 patients in the SLE cohort; and 9095 patients in the SSc cohort. After the GWAS result of the JIA cohort was meta-analyzed with the UK JIA cohort, there was a total of 4550 JIA cases and 18 446 controls. The analysis revealed a significant global correlation between JIA and adult rheumatic diseases, with 84 regions harboring signals associated with multiple diseases. Cross-trait analyses uncovered novel disease loci and 20 loci associated with JIA and adult diseases. Mendelian randomization analysis revealed the significant association of 11 proteins with rheumatic disorders. Both shared, organ-specific, and disease-specific critical cell types were highlighted.

**Conclusions and Relevance:**

In this genetic association study, there was significant genetic overlap between JIA and adult rheumatic diseases. These findings may help to refine JIA classification, risk stratification, and therapeutic strategy of repurposing adult disease drugs for pediatric patients with similar mechanisms.

## Key Points

**Question:**

What genetic loci may pose high risk of developing rheumatic diseases in adulthood for patients with juvenile idiopathic arthritis (JIA)?

**Findings:**

This genetic association study of 33 207 patients with JIA or adult rheumatic disease revealed significant genetic correlations, with 84 genomic regions harboring signals associated with multiple diseases, notably highlighting the janus kinase signal transducers and activators of transcription pathway. Cross-trait analyses uncovered 20 genomic loci conferring associations of JIA with adult diseases.

**Meaning:**

These findings suggest that genomics-informed disease therapeutics and prevention is crucial for improving JIA treatment strategies and patient outcomes, and aids in repurposing of drugs proven effective and safe in adult rheumatic diseases for pediatric conditions with similar pathogenic mechanisms.

## Teaser

This genetic association study analyzes associations of genomic loci with adult rheumatic diseases among patients with juvenile idiopathic arthritis.

## Introduction

Juvenile idiopathic arthritis (JIA) is widely recognized for its clinical and molecular similarities to arthritis of adult onset. Although the overall prognosis for children with JIA has significantly improved with treatment using nonsteroidal anti-inflammatory drugs and disease-modifying antirheumatic drugs, approximately 25% to 50% of patients continue to have the disease into adulthood and require ongoing medication.^1,2^ Certain subtypes correspond to specific adult autoimmune rheumatic diseases, albeit with different names than those used in adult forms.^3^ For example, rheumatoid factor (RF)–positive oligoarticular JIA is comparable to seropositive rheumatoid arthritis (RA); the enthesitis-related arthritis subtype of JIA falls within the broader spectrum of spondylarthritis, which includes ankylosing spondylitis in adults. Early-onset oligoarticular JIA and a subset of RF-negative polyarticular JIA closely resemble adult seronegative diseases. Systemic JIA shares clinical and genetic features with adult-onset Still disease.^2^ The current classification aligns certain JIA subtypes with adult rheumatic diseases, albeit under different terminologies.^4^

Moreover, JIA and RA have comorbidities with other rheumatic diseases such as systemic lupus erythematosus (SLE) and systemic sclerosis (SSc). It has been reported that children with JIA are at an increased risk of developing SLE and SSc in adulthood.^5^ These diseases (RA, SLE, and SSc), despite being distinct, share syndrome overlap, including tissue and organ damage, inflammation, and serological indicators.^6^ Despite the heterogenous nature of rheumatic diseases, it is well documented that there is obvious family aggregation.^7,8^ Patients with polyautoimmunity may have multiple autoimmune rheumatic diseases, including JIA, SLE, and SSc.^9^ The shared characteristics among these diseases suggest overlapping pathways and common biological mechanisms.^10^ Gaining a deeper understanding of these similarities could offer a more comprehensive perspective on joint inflammation and potentially lead to a broader range of treatment options; this is particularly relevant for JIA, which can benefit substantially from such advancements in treatment.^3^

In this study, we hypothesized that pediatric and adult rheumatic diseases may exhibit substantial shared genetic causes. Specifically, we proposed that patients with JIA carrying risk alleles that showed consistent directional associations with these diseases could face an increased susceptibility to persistent or recurrent conditions into adulthood. Such integrative analysis is crucial for refining JIA classification and developing targeted, effective treatments in the era of precision medicine.

## Methods

### Ethics Approval

This genetic association study of the JIA cohort^19^ was approved by the institutional review board of the Children’s Hospital of Philadelphia and written informed consent was obtained from all the participants or their parents. The study adhered to the Strengthening the Reporting of Genetic Association Studies (STREGA) reporting guideline and was conducted in accordance with the Helsinki Declaration.^38^

### Methods

**Figure 1.**
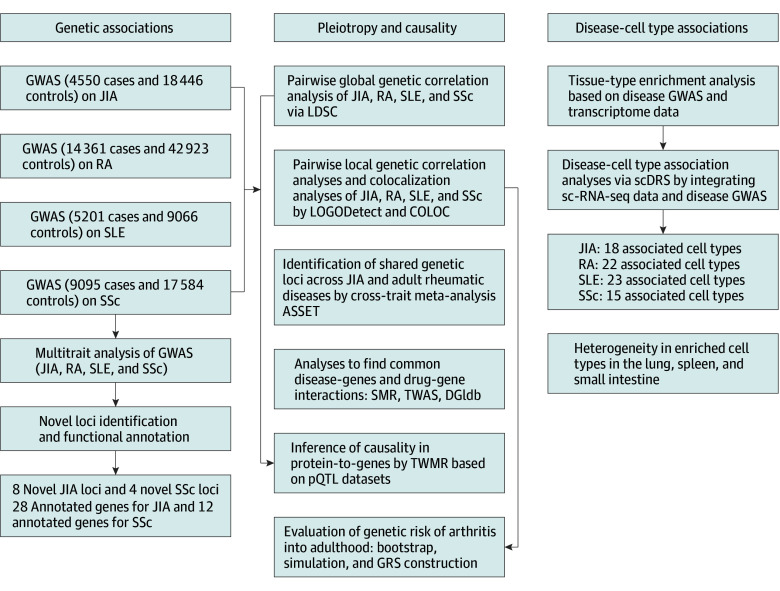
Overview of the Study Design and Analyses ASSET indicates association analysis based on subsets; COLOC, colocalization; DGIdb, Drug-Gene Interaction database; GRS, genetic risk score; GWAS, genome-wide association study; JIA, juvenile idiopathic arthritis; LDSC, linkage disequilibrium score regression; LOGODetect, local genetic correlation detector; pQTL, protein quantitative trait loci; RA, rheumatoid arthritis; scDRS, single-cell disease-relevance score; sc-RNA-seq, single-cell RNA sequencing; SLE, systemic lupus erythematosus; SMR, summary-databased mendelian randomization; SSc, systemic sclerosis; TWAS, transcriptome-wide association study; TWMR, 2-sample mendelian randomization.

We conducted a genetic association study on JIA and adult rheumatic diseases among 4 cohorts^19,20,21,22^ (JIA, RA, SLE, and SSc) who were of European ancestry; these studies were conducted between 2013 and 2024. Patients in the JIA cohort were recruited from the US, Australia, and Norway (with a UK cohort included in the meta-analyzed cohort),^19^ The study design is presented in Figure 1, and the information of the 4 disease cohorts^19,20,21,22^ (JIA, RA, SLE, and SSc) is shown in eTable 1 in Supplement 1.

### Statistical Analysis

After data quality control, linkage disequilibrium score regression^11^ was used to estimate single nucleotide variation (SNV [formerly SNP])–based global correlations among these diseases with the statistical significance threshold at *P* < 8.3 × 10^−3^ (.05/6) for Bonferroni correction. Multitrait analysis of genome-wide association studies (GWAS [MTAG])^12^ was performed between disease pairs with maximum false discovery rate being calculated. Association analysis based on subsets ^13^ was used for cross-trait meta-analysis, and the discrete local maximum method was used for multiple testing correction over the disease combinations. Local genetic correlations were analyzed using local genetic correlation detector ^14^ and bayesian colocalization^15^ with adjusted *P* values calculated for the number of independent genomic regions tested. Bootstrap resampling^16^ and simulation^17^ based on the real genotype of the JIA cohort and genetic risk score (GRS) construction^18^ were employed for risk modeling and predictive assessment. The Mann-Whitney *U* Test was used for markers’ bootstrap rank comparison. GRS comparison between JIA cases and controls was conducted via a 2-sided *t* test. The human reference genome build GRCh37 was used in all analyses. Additional detailed methodologies are provided in the eMethods in Supplement 1. Analysis was conducted using R software version 4.4.1 (R Project for Statistical Computing). Analyses were conducted from September 2023 to April 2024.

## Results

This study included 33 207 patients across the 4 cohorts, with 4550 patients in the meta-analyzed JIA cohort (including our JIA cohort: 1485 patients with arthritis onset before 16 years; 1017 female [68.5%], 10 352 controls; UK cohort: 3305 patients with JIA; 9196 controls^19^), 14 361 patients in the RA cohort, 5201 patients in the SLE cohort; and 9095 patients in the SSc cohort. After the GWAS result of our JIA cohort was meta-analyzed with the UK JIA cohort, there was a total of 4550 JIA cases and 18 446 controls. The detailed demographic description of the published RA,^20^ SLE,^21^ and SSc^22^ cohorts is given in their original publications. Information on the GWAS summary statistics of each cohort is presented in eTable 1 in Supplement 1.

Through a systematic investigation of the genetic association of JIA with adult rheumatic diseases (RA, SLE, and SSc) by incorporating genomic, transcriptomic and proteomic data, we pinpointed the candidate common genes underlying the risk of developing adult rheumatic diseases in patients with JIA, as well as genes contributing to the comorbidity of rheumatic diseases (Figure 1).

### Genetic Correlation Between JIA and Adult Rheumatic Diseases

**Figure 2.**
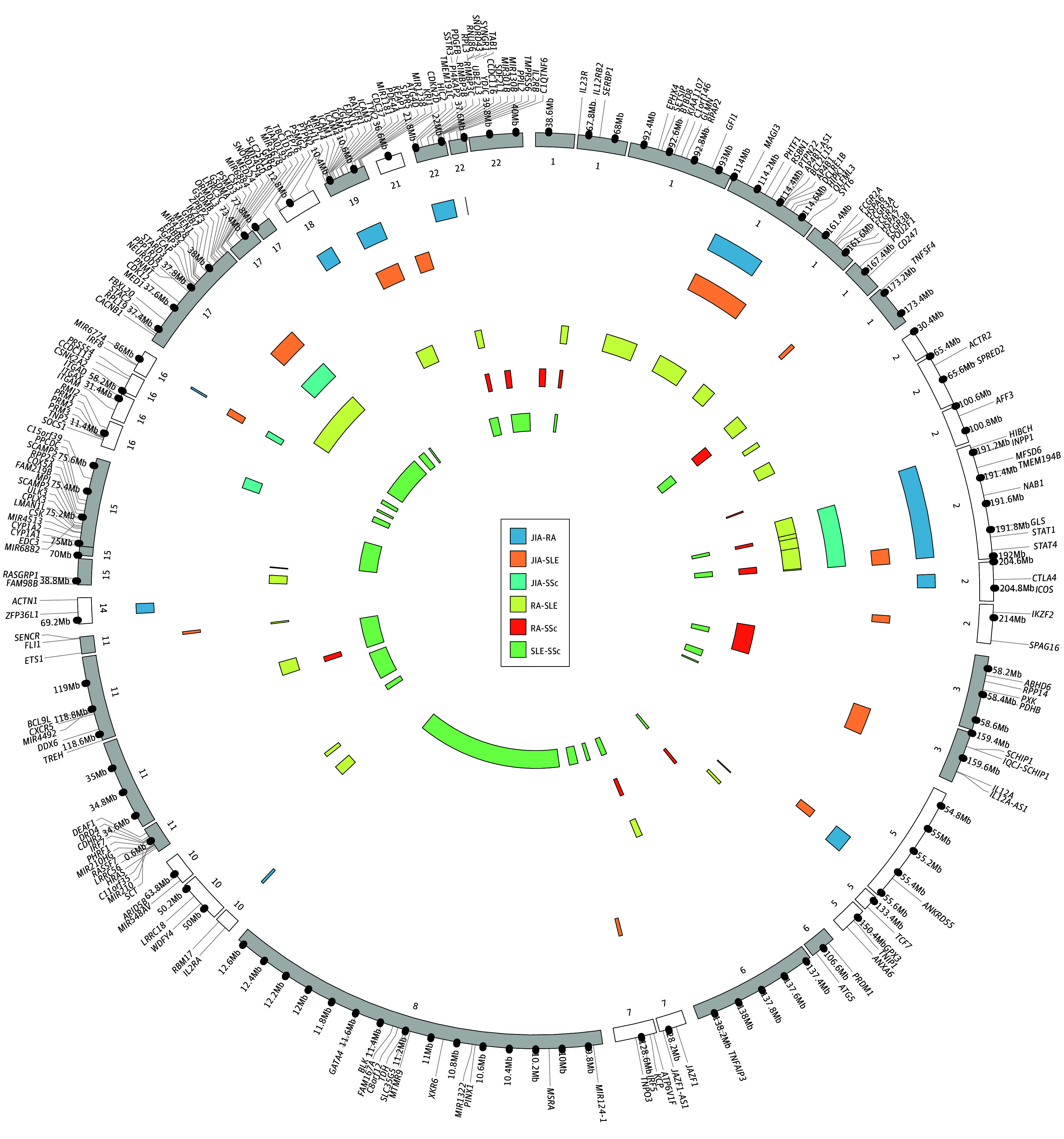
Circos Plot Showing the Shared Genetic Regions Between Pairs of the 4 Rheumatic Diseases Each colored track presents 1 pairwise disease. The labeled genes passed nominal statistical significance (*P* < .05) conducted by the Genome-Wide Complex Trait Analysis software package for colocalized genetic regions. JIA indicates juvenile idiopathic arthritis; Mb, megabase; RA, rheumatoid arthritis; SLE, systemic lupus erythematosus; SSc, systemic sclerosis.

Initially, we assessed the global genetic correlation between JIA and the adult diseases using LDSC.^11^ Significant positive correlations (*r_g_*) were observed among all pairs of rheumatic diseases. Notably, the genetic correlation between JIA and every adult rheumatic disease was significant (Figure 2 and eTable 2 in Supplement 1). RA exhibited genetic correlation with SLE (*r_g_* = 0.48) and SSc at similar scales (*r_g_* = 0.49), but SSc showed a larger-magnitude genetic correlation with SLE (*r_g_* = 0.84; *P* = 9.38 × 10^−15^).

### Common Genetic Regions in JIA and Adult Rheumatic Diseases

Considering the global genetic correlation, we further sought to investigate genomic segments commonly associated with JIA and adult rheumatic diseases. We excluded the human leukocyte antigen region due to its complex linkage disequilibrium structure (chromosome 6:29Mb-32Mb). Initially, we used local genetic correlation detector ^14^ to estimate genetic segments shared between pairwise diseases. A total of 167 regions were identified with consistent correlations across pairwise diseases (eTable 3 in Supplement 1). Subsequently, we conducted colocalization analysis^15^ to pinpoint genetic signals within these regions associated with both diseases, either through the same variant or variants in linkage disequilibrium . The analysis revealed that 84 regions harbored common signals associated with more than 1 disease (Figure 2 and eTable 4 in Supplement 1).

By aggregating SNV association *P* values to the gene level using the Genome-Wide ComplexTrait Analysis (GCTA-fastBAT) software package version 1.94 (Yang Lab; Westlake University),^23^ we identified 213 genes at these loci associated with pairwise diseases with nominal *P* values less than .05 (Figure 2), with significant enrichment of expression in blood, muscle, and other multiorgans (eFigure 1 in Supplement 1). Pathway enrichment analysis showed that these genes are enriched in helper T cells signaling pathways (eFigure 2 and eTable 5 in Supplement 1). Furthermore, the janus kinase signal transducers and activators of transcription (JAK-STAT) signaling pathway was enriched across each disease pair, highlighting its crucial role in the pathogenesis of rheumatic diseases. The effective application of JAK-targeting regimens reported in clinical trials^24,25,26,27,28^ emphasizes the association of a patient’s genetic profile with potential benefits from precision medicines.

### Rheumatic-Disease Loci

Building on the extended genetic correlation and colocalization findings, we conducted MTAG analyses between JIA and each adult rheumatic disease. We identified 8 novel genome-wide significant loci (P < 5 × 10^−8^) for JIA from the MTAG analyses (specifically, 7 from JIA-RA pairwise analysis, 4 from JIA-SLE analysis, and 2 from JIA-SSc analysis) (Figure 2 and eFigures 3-7 and eTable 6 in Supplement 1). Notably, the novel loci close to genes *IL12A-AS1* and *IRF5* were found in all 3 pairwise analyses but were indexed by different SNVs in 3 analyses (eFigures 4-6 in Supplement 1). MTAG analysis between SSc and the other 2 rheumatic diseases yielded 4 novel loci being significantly associated with SSc (eFigure 3, eFigure 8, and eTable 6 in Supplement 1).

Through conditional analysis, we observed 2 independent signals at 1 SSc locus and additional independent signals within chromosome 7 (128.5-128.8Mb region) for MTAG results of multiple disease pairs (JIA-RA, JIA-SLE, and JIA-SSc) (eFigure 8 and eTable 7 in Supplement 1). We mapped the novel loci to 28 candidate genes for JIA and 12 genes for SSc by integrating chromatin interactions evidence and expression quantitative trait loci (eQTL) data (eFigures 9-12 in Supplement 1).

### Common Genetic Loci

In light of the genetic association at local genomic segments, we conducted a 2-sided association analysis based on subsets^13^ to further pinpoint loci shared across rheumatic diseases. Twenty loci with genome-wide significance showed common associations of JIA with the 3 adult rheumatic diseases (Table). We found 5 independent loci with discordant associations across the 4 diseases (eTable 8 and eFigure 13 in Supplement 1).

**Table.** Loci With Common Associations of JIA With Adult Rheumatic Disorders

Single nucleotide variation	Chromosome	bp (GRCh37)	Effect allele	Reference allele	Frequency^a^	*P* value from ASSET analysis	Subset 1^b^	Subset 2^b^	Nearest gene	Variant annotation
*rs2476601*	1	114377568	A	G	0.10	6.92 × 10^−150^	JIA, RA, and SLE	NA	*PTPN22*	Exonic
*rs2056626*	1	167420425	G	T	0.41	5.23 × 10^−17^	NA	JIA, SLE, and SSc	*CD247*	Intronic
*rs2009094*	2	100765077	A	G	0.36	2.27 × 10^−15^	NA	JIA, RA, and SSc	*AFF3*	Intergenic
*rs4853458*	2	191959489	A	G	0.23	7.22 × 10^−71^	JIA, SLE, and SSc	NA	*STAT4*	Intronic
*rs1020195*	2	214089238	A	G	0.10	3.47 × 10^−8^	NA	JIA and SLE	*SPAG16*	Intergenic
*rs60020651*	3	46287651	C	A	0.14	2.38 × 10^−10^	NA	JIA and RA	*CCR3*	Intronic
*rs485499*	3	159745863	C	T	0.41	6.18 × 10^−19^	NA	JIA, SLE, and SSc	*IL12A-AS1*	Intronic
*rs10065637*	5	55438851	T	C	0.21	1.37 × 10^−23^	NA	JIA, RA, and SLE	*ANKRD55*	Intronic
*rs1002658*	6	137981584	T	C	0.18	2.94 × 10^−8^	NA	JIA and RA	*TNFAIP3*	Intergenic
*rs729302*	7	128568960	C	A	0.32	6.86 × 10^−26^	NA	JIA, SLE, and SSc	*IRF5*	Intergenic
*rs57593539*	8	129567515	A	G	0.13	2.43 × 10^−11^	NA	JIA, RA, SLE, and SSc	*LINC00824*	Intronic
*rs3134883*	10	6100725	A	G	0.30	1.86 × 10^−13^	JIA and RA	NA	*IL2RA*	Intronic
*rs17630235*	12	112591686	A	G	0.42	1.12 × 10^−15^	JIA, RA, and SLE	NA	*TRAFD1*	Downstream transcript
*rs7319041*	13	40345356	A	G	0.34	1.89 × 10^−13^	NA	JIA, RA, and SSc	*COG6*	Intronic
*rs35406824*	13	42957324	A	G	0.22	2.94 × 10^−11^	RA, SLE, and SSc	JIA	*RP11-413N19.2*	Intergenic
*rs2236262*	14	69261472	A	G	0.49	1.09 × 10^−10^	JIA	RA	*ZFP36L1*	Intronic
*rs17445836*	16	86017663	A	G	0.22	8.94 × 10^−17^	NA	JIA, SLE, and SSc	*LOC124903741*	ncRNA intronic
*rs9303277*	17	37976469	C	T	0.48	5.19 × 10^−23^	NA	JIA, RA, SLE, and SSc	*IKZF3*	Intronic
*rs2278442*	19	10444826	G	A	0.35	2.95 × 10^−18^	NA	JIA, RA, SLE, and SSc	*ICAM3*	Intronic
*rs7247222*	19	18392873	C	A	0.37	1.43 × 10^−8^	NA	JIA, SLE, and SSc	*JUND*	Upstream transcript

Abbreviations: ASSET, association analysis based on subsets; bp, position on human genome build hg19; JIA, juvenile idiopathic arthritis; NA, not applicable; RA, rheumatoid arthritis; SLE, systemic lupus erythematosus; SSc, systemic sclerosis.

^a^ Frequency of the effect allele.

^b^ Subset 1 and subset 2 refer to the subset combinations in the positive and negative directions, respectively.

### Common Genes and Drug-Gene Interactions

To elucidate how these common loci influence disease development, we conducted target gene mapping by integrating blood eQTL data through summary-databased mendelian randomization (MR).^29^ Our analysis identified 30 genes associated with JIA, 87 genes associated with RA, 64 genes associated with SLE, and 15 genes associated with SSc (eTable 9 in Supplement 1). Transcriptome-wide association studies incorporating the whole blood expression model from genotype tissue expression revealed more genes significantly associated with these diseases (eTable 10 in Supplement 1). By combining results from both analyses, we identified 43 genes that may have common influences on 2 or more traits (eTable 11 and eFigure 14 in Supplement 1), including 28 genes located in the shared genomic segments from the local genetic correlation analyses.

Among the common genes, *IL12RB2, PXK, IL12A, DRD4, CSK, PNMT,* and *TYK2* were druggable genes with properties suitable for drug targeting, according to the Drug Gene Interaction Database.^30^ These genes had interactions with 223 drugs (antagonists, inhibitors, or agonists) (eTable 12 in Supplement 1).

### Identification of Significantly Associated Proteins

The cellular functions of target genes are typically carried out by their protein products, whose expression may not consistently match mRNA levels. To identify proteins crucial to the pathogenesis of the autoimmune rheumatic diseases, we performed MR analysis based on the protein QTL (pQTL) data obtained from 4 large-scale GWAS on blood plasma proteins.^31^ Considering potential unknown pleiotropy between instrumental variables and outcomes, we focused on cis-pQTL variants as instrumental variables to infer important associations.

**Figure 3.**
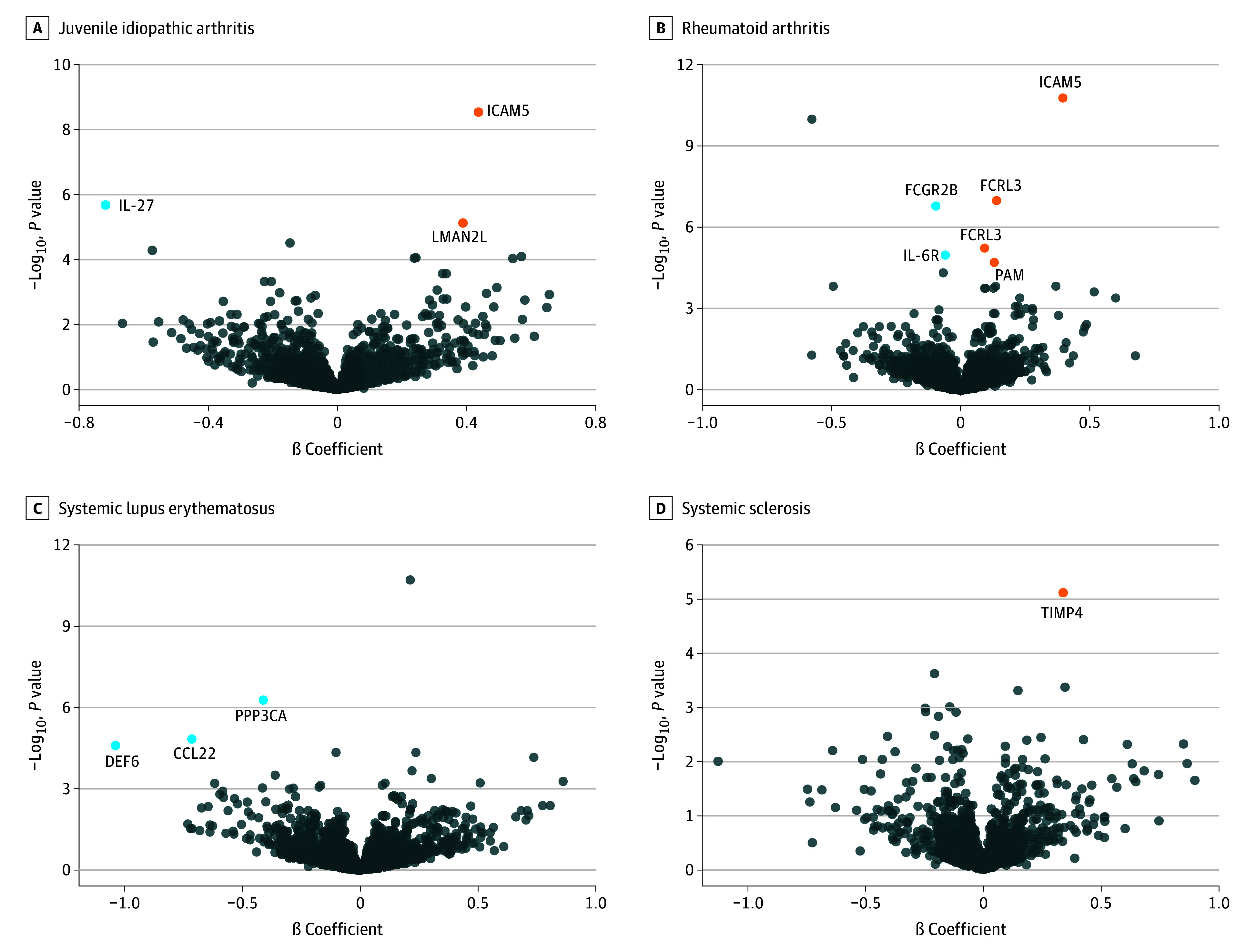
Mendelian Randomization Analyses With Cis-Protein Quantitative Trait Locus Data for Significantly Associated Proteins The circles represent the results for proteins on 4 autoimmune rheumatic diseases. Labeled proteins reached the significance threshold after Bonferroni correction. Blue indicates the proteins with a negative association with diseases, while orange indicates positive associations.

We found that 11 proteins had a significant association with rheumatic disorders with Bonferroni correction (eTable 13 in Supplement 1), namely, interleukin-27, ICAM5, LMAN2L with JIA; TIMP4 with SSc; and 6 proteins with RA, and 4 proteins with SLE (Figure 3). Due to the limited availability of pQTL data, the protein-based MR analysis identified only a few candidate proteins for each disease.

Four of these proteins showed significant associations with rheumatic disorders at the mRNA level. Summary-data–based MR results for ICAM5, FCRL3 and DEF6, as well as transcriptome-wide association study results for FCRL3, PAM, and DEF6, reached the significance threshold following Benjamin and Hochberg correction (eTable 9 and eTable 10 in Supplement 1).

We further assessed the druggability of these proteins, in comparison with the findings in the study by Finan et al.^32^ In total, 8 of 11 proteins were considered druggable, including 3 in tier 1 position (interleukin-6R, FCGR2B, and PPP3CA), 1 in tier 2 (PAM), 2 in tier 3A (interleukin-27 and FCRL3), and 2 in tier 3B (CCL22 and TIMP4) (eTable 13 in Supplement 1).

### Construction of GRS for Arthritis in Adulthood

Given the established association of JIA with RA, including consistent reports of incidence rates for patients with JIA experiencing progression to adult forms of arthritis,^1,2^ we assessed the potential genetic susceptibility of individuals with JIA to later development of arthritis in adulthood. Through bootstrap resampling^16^ and simulation of the 1245 patients in our JIA cohort, we assessed the ranking of the RA index SNVs within the 27 genomic regions concordantly correlated between JIA and RA (eTable 3 in Supplement 1). The bootstrap confidence intervals for most rankings were wide, except for the autoimmune risk locus *PTPN22*, which consistently ranked as the top in every bootstrap (eFigure 15 in Supplement 1). Encouragingly, index SNVs from the 10 regions identified by both local correlation and colocalization analyses (eTable 4 in Supplement 1) tended to rank higher than the other SNVs, with 70% (7 of 10 SNVs) ranking in the top half in terms of median rank. Additionally, we constructed GRS for RA based on the dosage of the risk allele at the 27 index SNVs in each patient with JIA. The resultant GRS followed a normal distribution across patients with JIA (eFigure 15 in Supplement 1). As expected, GRS was significantly higher on average for patients with JIA (GRS, 0.96; 95% CI, 0.95-0.97) compared with controls (GRS, 0.89; 95% CI, 0.89-0.90) (*P* = 2.19 × 10^−39^) and demonstrated potential to distinguish between cases and controls across simulations (empirical *P* value <.001). Patients with JIA carrying a greater polygenic burden for established RA risk variants may face elevated likelihood of persistent or recurrent arthritis later in life. Lifespan genomic datasets are needed to validate our hypothesized model of shared genomic risk loci impacting rheumatological disease trajectories from childhood through adulthood.

### Shared and Disease-Specific Cell-Type Associations With JIA and Adult Rheumatic Diseases

Our initial tissue enrichment analysis revealed that blood, spleen, lung, and small intestine were enriched for expressed genes at the GWAS loci of these 4 autoimmune rheumatic diseases (eFigure 16 in Supplement 1). These are the organs commonly affected in patients. To further characterize specific disease-associated cell types, we conducted single-cell disease relevance score analysis^33^ by integrating original GWAS summary statistics with single cell RNA-sequencing data.

**Figure 4.**
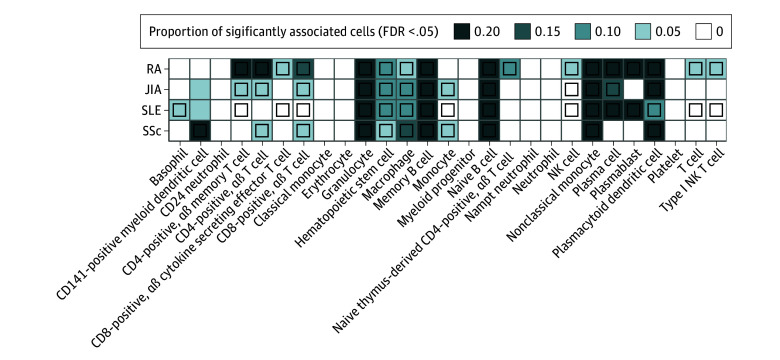
Associations of Cell Types in Blood With Genetic Risk of Autoimmune Rheumatic Disorders The heatmap depicts associations of cell type with disease for each of the 4 diseases. The darkness of heatmap colors denotes the proportion of significantly associated cells for each disease. Squares represent significant associations of cell type with disease with multiple testing adjustment (false discovery rate [FDR] < .05). aβ indicates amyloid-β; JIA juvenile idiopathic arthritis; NK, natural killer; RA, rheumatoid arthritis; SLE, systemic lupus erythematosus; SSc, systemic sclerosis.

We found that genes associated with all 4 rheumatic diseases were highly enriched in 5 blood cell types: granulocytes, naive B cells, memory B cells, nonclassical monocytes, and plasmacytoid dendritic cells. This enrichment underscores the important roles of antigen presentation and type I interferon production in the pathogenesis of both pediatric and adult rheumatic diseases (Figure 4 and eFigure 17 in Supplement 1). Fewer immune cell types showed significant enrichment in the lung, spleen, and small intestine, but with prominent heterogeneity across individual cells (eFigures 18-20 in Supplement 1).

Disease-specific associations were found with certain cell types in the blood, spleen, lung, and small intestine, such as CD141-positive myeloid dendritic cells with SSc and erythrocytes in the spleen with SLE (Figure 4 and eFigure 19 in Supplement 1), which may be an important triggering source of type I interferon disturbance in SLE. Interestingly, we observed significant associations of hematopoietic stem cells (HSC) in the spleen and transit amplifying cells in the small intestine with SLE (eFigure 19 and eFigure 20 in Supplement 1).

## Discussion

We conducted a comprehensive genetic association study to explore the associations of JIA with RA, SLE, and SSc from different aspects, including global genetic correlation, local genetic correlation, and shared genetic risk loci. While there may be a causal relationship between pediatric disease and adult-onset conditions, the pervasive local genetic correlations and existing functional evidence supporting shared molecular pathways suggest that current analytical methods may lack sufficient sensitivity to reliably detect horizontal pleiotropy in this context. This limitation weakens the ability to draw definitive causal inferences between JIA and adult rheumatological conditions using standard MR approaches.

Additional cross-trait genomic analyses revealed 20 loci commonly associated with JIA and adult autoimmune rheumatic diseases. Notably, 5 loci had opposite effect directions between the diseases identified in cross-trait meta-analyses. For instance, the T allele of *rs17457484* at 3’ of *AKAP11*, potentially regulating *TNFSF11*, was associated with increased risk for all 3 adult diseases but decreased risk for JIA. The differing effect directions could potentially be explained by cell-type and context-dependent regulation by transcription factors. As evidenced by our findings, the key cell types involved in rheumatic diseases may differ, and transcription factor expression profiles vary in different cell types and change throughout life. The opposite associations could also be mediated by distinct molecular pathways.^34^ In addition, such discordant associations have been conceptualized as antagonistic pleiotropy in evolutionary theories of aging, whereby variants impart early-life advantages and later-life costs, allowing population-level selection.^35^ By mitigating overall fitness impact, antagonistically pleiotropic alleles may evade negative selection. Their cumulative impacts are proposed to drive biological aging over the long term.

The differences in candidate genes identified through eQTL and transcriptome-wide association study compared with candidate proteins discovered via incorporating of pQTL data highlight the importance of protein-level data, which are essential for translating GWAS loci into biologically meaningful insights regarding SNV function and disease pathogenesis. Protein abundance, interactions, conformational changes, and posttranslational modifications are critical for cellular functions involved in disease pathogenesis.

Our analysis revealed an association of SLE with HSC and erythrocytes in the spleen, as well as transit amplifying cells in the small intestine. SLE is considered a stem cell–mediated disease^36^ with some patients also exhibiting defects in the HSC of the spleen. HSC transplantation has been administrated for all 4 autoimmune rheumatic diseases studied, but certain indications have shown high relapse rates.^37^ Our study suggests that patients with SLE with specific genotypes may be suitable candidates for HSC transplantation, although not all patients may benefit from this treatment.

### Limitations

This study has several limitations. First, because no existing GWAS study has longitudinally followed up patients with JIA into adulthood, we could not directly validate whether the shared genetic loci identified predispose them to the later development of specific adult rheumatic diseases. Second, without accessible sex-stratified GWAS data on adult disease cohorts, we were unable to examine potential sex-specific differences in genetic architecture and long-term progression. Autoimmune disorders are known to be more prevalent among females, so dissecting the genetic impacts by sex could provide valuable insight. Third, our analysis was restricted to patients of European ancestry due to limitations in available GWAS data. However, the subtype distribution of JIA varies between ethnicities, so investigating shared genetics across ancestries could uncover population-specific risk factors. Continued collection of diverse, well-annotated longitudinal cohorts will be important to address these limitations and help determine the robustness and clinical applicability of shared loci over the life course.

## Conclusions

In this genetic association study of JIA and adult rheumatic disease, we revealed significant genetic correlations between diseases, identified novel and shared loci between diseases, and highlighted the JAK-STAT pathway, resonating with clinical observations. We further built a GRS model of arthritis progression into adulthood for patients with JIA on the basis of the RA assessment of 27 genomic regions concordantly associated with JIA and RA. We also identified proteins with a significant association with rheumatic disorders and found both shared and disease-specific critical cell types. Our findings advocate for a genomic-based classification strategy for JIA and suggest the repurposing of drugs that are effective and safe in treating adult autoimmune rheumatic conditions for use in pediatric patients with similar pathogenic mechanisms.

## Notes

Supplement 1. **eTable 1.** Summary of the Rheumatic-Disease Cohorts Included in the Study
eMethods.
**eTable 2.** Pairwise Genetic Correlation Estimation Using LDSC (rg and P-Value) **eTable 3.** Genomic region With Concordant Associations Among Autoimmune Rheumatic Diseases Estimated by LOGODetect **eTable 4.** The Genomic Regions With Shared Effects Among Rheumatic Diseases **eFigure 1.** Tissue Enrichment of Genes in the Genomic Regions Having Shared Associations With Pairs of Rheumatic Diseases **eFigure 2.** Significantly Enriched KEGG Pathways for Genes in the Colocalized Genetic Regions Associated With Pairwise Diseases **eTable 5.** KEGG Pathways Enrichment for Genes in the genomic Regions With Shared Associations Among Rheumatic Diseases **eFigure 3.** Manhattan Plots Summarizing Results From Bivariate MTAG Analyses Between JIA and Adult Rheumatic Diseases **eFigure 4.** Regional Association Plots of the 7 Novel JIA Loci From Bivariate MTAG Between JIA and RA **eFigure 5.** Regional Association Plots of the 4 Novel JIA Loci From Bivariate MTAG Between JIA and SLE **eFigure 6.** Regional Association Plots of the 2 Novel JIA Loci From Bivariate MTAG Between JIA and SSc **eFigure 7.** Regional Association Plots of the 4 Novel SSc Loci From Bivariate MTAG Between SSc and SLE **eTable 6.** Lead SNPs (P < 5×10−8 and r2 < 0.1) From MTAG Analyses **eFigure 8.** Conditional Analyses on the Three JIA Novel Loci and One SSc Novel Locus From MTAG Analysis **eTable 7.** Conditional Analysis Yielded Additional Association Signals at the Novel Loci for JIA and SSc **eFigure 9.** Circos Plots of Chromatin Interactions and eQTLs at the 7 Novel JIA Loci From Bivariate MTAG Between JIA and RA **eFigure 10.** Circos Plots of Chromatin Interactions and eQTLs at the 4 Novel JIA Loci From Bivariate MTAG Between JIA and SLE **eFigure 11.** Circos Plots of Chromatin Interactions and eQTLs at the 2 Novel JIA Loci From Bivariate MTAG Between JIA and SSc **eFigure 12.** Circos plots of chromatin interactions and eQTLs at the 4 novel SSc loci from bivariate MTAG between SSc and SLE **eTable 8.** Loci With Opposite Directions of Effect **eFigure 13.** The Five Independent Loci With Effects in Opposite Directions Across the Four Diseases **eTable 9.** Candidate Genes Identified by SMR With FDR <0.05 **eTable 10.** Candidate Genes Identified by TWAS With FDR <0.05 **eTable 11.** Genes Having Common Significant Effects on Two or More Rheumatic Diseases **eFigure 14.** Venn Diagram of Genes Which Are Significantly Associated With 4 Autoimmune Rheumatic Diseases Identified by SMR and TWAS (FDR ≤ 0.05) **eTable 12.** Drug-Gene Interactions Among Genes at the Common Loci Associated With Rheumatic Diseases **eTable 13.** MR Results for Blood Proteins Obtaining a Significant MR P Value (after Bonferroni Correction) for Association With Rheumatic Disorders **eFigure 15.** Construction of Genetic Risk Score of Progression to RA for JIA Patients **eFigure 16.** Tissue Expression Analysis of 30 General Tissue Types Obtained From Genotype-Tissue Expression Version 8 (GTEx v8) Using MAGMA Gene-Property Test for 4 Rheumatic Diseases **eFigure 17.** The UMAPs Showing the Associations of Cell Types in Blood With Genetic Risk of Autoimmune Rheumatic Disorders **eFigure 18.** Associations of Cell Types in Lung With Genetic Risk of Autoimmune Rheumatic Disorders **eFigure 19.** Associations of Cell Types in Spleen With Genetic Risk of Autoimmune Rheumatic Disorders **eFigure 20.** Associations of Cell Types in Small Intestine With Genetic Risk of Autoimmune Rheumatic Disorder
eReferences

Supplement 2.
Data Sharing Statement
