# Supplementary material for: Genetic Association of Juvenile Idiopathic Arthritis With Adult Rheumatic Disease
Source: JAMA Netw Open. 2024 Dec 27;7(12):e2451341. doi: 10.1001/jamanetworkopen.2024.51341 (PMC11681380; doi:10.1001/jamanetworkopen.2024.51341)
Supplement: Supplement 1. — eTable 1. Summary of the Rheumatic-Disease Cohorts Included in the Study eMethods. eTable 2. Pairwise Genetic Correlation Estimation Using LDSC (rg and P-Value) eTable 3. Genomic region With Concordant Associations Among Autoimmune Rheumatic Diseases Estimated by LOGODetect eTable 4. The Genomic Regions With Shared Effects Among Rheumatic Diseases eFigure 1. Tissue Enrichment of Genes in the Genomic Regions Having Shared Associations With Pairs of Rheumatic Diseases eFigure 2. Significantly Enriched KEGG Pathways for Genes in the Colocalized Genetic Regions Associated With Pairwise Diseases eTable 5. KEGG Pathways Enrichment for Genes in the genomic Regions With Shared Associations Among Rheumatic Diseases eFigure 3. Manhattan Plots Summarizing Results From Bivariate MTAG Analyses Between JIA and Adult Rheumatic Diseases eFigure 4. Regional Association Plots of the 7 Novel JIA Loci From Bivariate MTAG Between JIA and RA eFigure 5. Regional Association Plots of the 4 Novel JIA Loci From Bivariate MTAG Between JIA and SLE eFigure 6. Regional Association Plots of the 2 Novel JIA Loci From Bivariate MTAG Between JIA and SSc eFigure 7. Regional Association Plots of the 4 Novel SSc Loci From Bivariate MTAG Between SSc and SLE eTable 6. Lead SNPs (P < 5×10^−8^ and r2 < 0.1) From MTAG Analyses eFigure 8. Conditional Analyses on the Three JIA Novel Loci and One SSc Novel Locus From MTAG Analysis eTable 7. Conditional Analysis Yielded Additional Association Signals at the Novel Loci for JIA and SSc eFigure 9. Circos Plots of Chromatin Interactions and eQTLs at the 7 Novel JIA Loci From Bivariate MTAG Between JIA and RA eFigure 10. Circos Plots of Chromatin Interactions and eQTLs at the 4 Novel JIA Loci From Bivariate MTAG Between JIA and SLE eFigure 11. Circos Plots of Chromatin Interactions and eQTLs at the 2 Novel JIA Loci From Bivariate MTAG Between JIA and SSc eFigure 12. Circos plots of chromatin interactions and eQTLs at the 4 novel SSc loci from bivariate MTAG between SS [file jamanetwopen-e2451341-s001.pdf]

Supplemental Online Content

Fan J, Hao J, Fu Y, et al. Genetic association of juvenile idiopathic arthritis with adult rheumatic disease. *JAMA Netw. Open*. 2024;7(12):e2451341. doi:10.1001/jamanetworkopen.2024.51341

eTable 1. Summary of the Rheumatic-Disease Cohorts Included in the Study
eMethods
eTable 2. Pairwise Genetic Correlation Estimation Using LDSC (rg and P-Value)
eTable 3. Genomic region With Concordant Associations Among Autoimmune Rheumatic Diseases Estimated by LOGODetect
eTable 4. The Genomic Regions With Shared Effects Among Rheumatic Diseases
eFigure 1. Tissue Enrichment of Genes in the Genomic Regions Having Shared Associations With Pairs of Rheumatic Diseases
eFigure 2. Significantly Enriched KEGG Pathways for Genes in the Colocalized Genetic Regions Associated With Pairwise Diseases
eTable 5. KEGG Pathways Enrichment for Genes in the genomic Regions With Shared Associations Among Rheumatic Diseases
eFigure 3. Manhattan Plots Summarizing Results From Bivariate MTAG Analyses Between JIA and Adult Rheumatic Diseases
eFigure 4. Regional Association Plots of the 7 Novel JIA Loci From Bivariate MTAG Between JIA and RA
eFigure 5. Regional Association Plots of the 4 Novel JIA Loci From Bivariate MTAG Between JIA and SLE
eFigure 6. Regional Association Plots of the 2 Novel JIA Loci From Bivariate MTAG Between JIA and SSc
eFigure 7. Regional Association Plots of the 4 Novel SSc Loci From Bivariate MTAG Between SSc and SLE
eTable 6. Lead SNPs ($P < 5\times10^{-8}$ and $r^2 < 0.1$) From MTAG Analyses
eFigure 8. Conditional Analyses on the Three JIA Novel Loci and One SSc Novel Locus From MTAG Analysis
eTable 7. Conditional Analysis Yielded Additional Association Signals at the Novel Loci for JIA and SSc
eFigure 9. Circos Plots of Chromatin Interactions and eQTLs at the 7 Novel JIA Loci From Bivariate MTAG Between JIA and RA
eFigure 10. Circos Plots of Chromatin Interactions and eQTLs at the 4 Novel JIA Loci From Bivariate MTAG Between JIA and SLE
eFigure 11. Circos Plots of Chromatin Interactions and eQTLs at the 2 Novel JIA Loci From Bivariate MTAG Between JIA and SSc
eFigure 12. Circos plots of chromatin interactions and eQTLs at the 4 novel SSc loci from bivariate MTAG between SSc and SLE
eTable 8. Loci With Opposite Directions of Effect
eFigure 13. The Five Independent Loci With Effects in Opposite Directions Across the Four Diseases
eTable 9. Candidate Genes Identified by SMR With FDR < 0.05
eTable 10. Candidate Genes Identified by TWAS With FDR < 0.05
eTable 11. Genes Having Common Significant Effects on Two or More Rheumatic Diseases

© 2024 Fan J et al. *JAMA Network Open.*

eFigure 14. Venn Diagram of Genes Which Are Significantly Associated With 4 Autoimmune Rheumatic Diseases Identified by SMR and TWAS (FDR ≤ 0.05)
eTable 12. Drug-Gene Intercations Among Genes at the Common Loci Associated With Rheumatic Diseases
eTable 13. MR Results for Blood Proteins Obtaining a Significant MR *P* Value (after Bonferroni Correction) for Association With Rheumatic Disorders
eFigure 15. Construction of Genetic Risk Score of Progression to RA for JIA Patients
eFigure 16. Tissue Expression Analysis of 30 General Tissue Types Obtained From Genotype-Tissue Expression Version 8 (GTEx v8) Using MAGMA Gene-Property Test for 4 Rheumatic Diseases
eFigure 17. The UMAPs Showing the Associations of Cell Types in Blood With Genetic Risk of Autoimmune Rheumatic Disorders
eFigure 18. Associations of Cell Types in Lung With Genetic Risk of Autoimmune Rheumatic Disorders
eFigure 19. Associations of Cell Types in Spleen With Genetic Risk of Autoimmune Rheumatic Disorders
eFigure 20. Associations of Cell Types in Small Intestine With Genetic Risk of Autoimmune Rheumatic Disorder
eReferences

This supplemental material has been provided by the authors to give readers additional information about their work.

eTable 1. Summary of the rheumatic-disease cohorts included in the study.

Disease	Abbreviation	Population	N case	N control	SNP number	Lambda 1000	h2
Juvenile Idiopathic Arthritis	JIA	European	4550	18446	6654778	1.016	0.197
Rheumatoid Arthritis	RA	European	14361	42923	6199502	1.002	0.150
Systemic Lupus Erythematosus	SLE	European	5201	9066	6957966	1.026	0.519
Systemic Scleroderma	SSc	European	9095	17584	4598108	1.007	0.110

N: sample size; SNP: single nucleotide polymorphism; SNP number: the number of SNPs after QC; h2: SNP-based heritability.

© 2024 Fan J et al. *JAMA Network Open.*

eMethods.

Quality control on GWAS summary statistics for LD score regression

Patients in the JIA cohort were recruited in the US, Australia, and Norway and the cohort included 1,485 patients with arthritis onset at age <16 years and 10,352 controls. Genome-wide association study (GWAS) result of this cohort was meta-analyzed with the UK cohort[1], yielding a combined study of 4550 JIA cases and 18446 controls. The meta-analysis has been described in our previous publication[2]. The GWAS summary statistics of the other three autoimmune rheumatic diseases were downloaded from the NHGRI-EBI GWAS Catalog (https://www.ebi.ac.uk/gwas/), including Rheumatoid Arthritis (RA, N = 54284)[3], Systemic Lupus Erythematosus (SLE, N = 14267)[4], Systemic sclerosis (SSc, N = 26679)[5] . All SNPs with MAF <1%, insertions, and deletion polymorphisms were excluded from the analysis. The study subjects in all four cohorts were of European ancestry.

We applied linkage disequilibrium score regression (LDSC)[6] to estimate the SNP-based global correlations between these diseases. The LDSC scores were calculated with 1000 Genomes Project EUR population as reference. All SNPs were aligned to human genome reference build GRCh37. The statistical significance threshold was set at $P < 8.3 \times 10^{-3}$ (0.05/6) for Bonferroni correction.

Multi-trait GWAS analysis

Multi-Trait Analysis of GWAS (MTAG)[7] was used for disease meta-analysis to improve statistical power by genetic correlation among multiple related traits. The max-FDR, a theoretical upper bound limit on the FDR for a GWAS, was calculated using standard settings.

Cross-trait meta-analysis with ASSET

Cross-trait meta-analysis of association analysis was by Association analysis based on SubSETs (ASSET)[8], which tests the association of each SNP with all possible subsets-combinations of traits and identifies the optimal trait-subset, to explore the pleiotropy among the four autoimmune rheumatic diseases. Two-sided ASSET was applied to detect associations in both same and opposite directions of effect.

Identification of novel loci and annotation

Clumping was conducted on genome-wide significant SNPs ($p < 5 \times 10^{-8}$) from MTAG analysis using PLINK[9]. We selected independent SNPs with the lowest P-value in each linkage disequilibrium block (LD, $r2 > 0.1$) within each 500 kilobase (kb) window. The LD relationship was computed from the 1000 Genomes EUR cohort (The 1000 Genomes Project Consortium, 2015).

A novel independent locus was defined as being outside 500Kb upstream and downstream of known disease loci and not in LD ($r2 > 0.1$) with any reported loci in the GWAS catalog. Considering the complexity of LD structure within the major histocompatibility complex region (MHC, Chr6: 27-35 Mb, GRCh37), we merged this region into one locus.

Stepwise conditional analysis at each novel locus (±200Kb) was conducted using GCTA COJO[10] for independent signals, continuing until the most significant p-values in conditional

© 2024 Fan J et al. *JAMA Network Open.*

analysis is above 1 x 10^{-5}.

SNP annotation was conducted using FUMA[11], including MAGMA analysis to assess tissue enrichment of variant-associated genes. Integration of chromatin conformation capture (HiC) data from spleen and GM12878 cells, along with eQTL data, enabled the mapping of putative regulated genes for novel index SNPs. Additionally, MAGMA analysis was conducted on the FUMA platform. The position of all the SNPs and genes is on human genome build GRCh37.

Local genetic correlation analysis

Genomic regions with significant local genetic correlations between pairwise traits were identified with the following pipeline. LOGODetect (LOcal Genetic cOrrelation Detector)[12] divides the whole genome into 224 independent blocks based on LD patterns and identifies pleiotropic loci between each pair of traits. Then, the identified pleiotropic loci between pairwise traits were subjected to Bayesian colocalization analysis by coloc[13] R package to compute the posterior probability of single variant associations with both traits. A combined posterior probability for H3 (two independent causal SNPs sharing association with pairwise traits in the same genomic region) and H4 (a single SNP association with pairwise traits) more than 90% or PP.H4 > 50% was considered as the evidence for colocalization. We excluded the SNPs within the *HLA* region from the analysis.

In addition, we conducted gene-based association analysis for each pleiotropic region using GCTA-fastBAT[14] and conducted pathway enrichment analysis on genes significantly associated with disease-pairs using the R package clusterProfile[15] with KEGG as the pathway resource.

Candidate gene prioritization

Summary-data-based Mendelian randomization (SMR) analysis was applied to prioritize candidate functional genes at each genome-wide significant locus by integrating eQTL and GWAS summary statistics. Blood cis-eQTL data were obtained from three databases: Genotype-Tissue Expression (GTEx)[16], the eQTLGen Consortium[17], and the Consortium for the Architecture of Gene Expression (CAGE)[18]. Candidate functional genes were defined as those with adjusted p-value < 0.05 (Benjaminiand Hochberg method) and showing no heterogeneity in the dependent instruments (HEIDI) test (P_{HEIDI}>0.05).

Additionally, TWAS (transcriptome-wide association studies) analysis using S-PrediXcan[19] was employed to infer the causality between genes and diseases with the MASHR blood expression model of GTEx, which provided complementary results to SMR. Significance was defined as FDR < 0.05 after Benjamini-Hochberg correction.

MR analysis to identify potential disease-causing proteins

We performed Mendelian randomization (MR) analysis to identify the potential causal association of proteins with autoimmune rheumatic diseases by integrating protein quantitative trait loci (pQTL) and GWAS data. Instruments variables (IVs) for blood proteins were obtained from the three GWAS cohorts, with pQTL data from: the INTERVAL cohort study by Sun et al.[20] , the pQTLs with 4,785 proteins from the SOMAscan platform by Emilsson et al.[21], the pQTLs with 807 proteins by Gudjonsson et al.[22], and 539 pQTLs identified by Suhre et al.[23].

IVs were filtered based on the following criteria: (1) Rare variants (MAF < 0.01) and palindromic SNPs were excluded. LD clumping was performed to select independent SNPs (p <

5 x 10-8, r2 > 0.001 within a 500 kb window), and F-statistics were calculated to assess the strength of the genetic instruments. IVs with an F-statistic greater than 10 were considered free from weak instrument bias[24]. (2) Cochrane's Q-statistic was applied to evaluate heterogeneity among the instrumental variables[25], and those with a p-value < 0.05 were excluded from further analysis. Leave-one-out analysis[26] was also employed to detect heterogeneous SNPs. (3) Horizontal pleiotropy was assessed using the MR-Egger regression test. An intercept close to 0 (p-value < 0.05) suggested an absence of horizontal pleiotropy. The MR-PRESSO test[27] was also used to address directional pleiotropy by removing outliers. Additionally, the Steiger test was applied to confirm the effect direction from exposure to outcome.

To account for potential unknown confounders between exposure and outcome, multiple MR methods were applied in the analysis. The inverse variance weighted (IVW) method was a classic method to calculate an accurate causal association in the absence of horizontal pleiotropy. The weighted median estimator (WM) was used to assess robust causality under the condition that more than 50% of the IVs exhibited pleiotropic bias[28]. For single IV analysis, the Wald ratio test was applied to estimate the effect on the outcome. The Bonferroni correction method was used to define an experiment-wide statistical significance threshold.

Polygenic enrichment analysis at single-cell level

We used scDRS[29] pipeline to investigate the tissue/cell type-specific impact by risk variants identified in GWAS. Initially, MAGMA analysis was used to compute the Z score and P-value of genes based on GWAS results and the top 1,000 genes were selected as putative disease-genes. Subsequently, a disease score for each cell type was computed by aggregating the expression of these putative disease genes, along with a set of 1,000 Monte Carlo control scores from a random gene set based on scRNA-seq data. The scores for each disease gene were then normalized and compared against the control scores; and disease-association P-values for individual cells were output. With the function of perform-downstream, we performed a cell-type level analysis to examine disease-associations by integrating cell-type annotations. Heterogeneity in disease-associations across individual cells within a given cell type was also assessed. The Benjamini Hochberg method was applied for multiple testing. The single-cell RNA sequencing data was from the Tabula Sapiens Consortium[30], with the data of 24 tissues or organs available.

Bootstrap and simulation

We performed bootstrap resampling[31] and simulation analyses[32]. Within each significantly and concordantly correlated region identified by local genetic correlation analysis between JIA and RA, we selected the index SNPs from the RA summary statistics derived from the MTAG analysis. Using real genotype data from 1,245 JIA patients in the CAG cohort, we performed 1,000 rounds of bootstrap resampling and simulation. Given an estimated arthritis prevalence of 0.25-0.5 in JIA patients transitioning to adulthood, each round randomly assigned case/control status to 400 and 600 individuals, respectively, based on genotypes and effect sizes of RA-risk alleles from the MTAG analysis. Sex was included as a covariate in logistic regression models to assess the association between each SNP and the assigned phenotype in each resample. Z-statistics were computed and ranked across all SNPs and resamples to derive bootstrap confidence intervals.

© 2024 Fan J et al. *JAMA Network Open.*

Genetic risk score (GRS) construction

We then constructed genetic risk scores (GRS)[33] based on these index SNPs at the genomic regions shared between JIA and RA. Specifically, GRS for RA was calculated using effect sizes from RA MTAG results and applied to assess risk in our JIA cohort. This approach allowed us to indirectly evaluate whether JIA patients carrying higher polygenic burdens for adult diseases might face an elevated risk of developing RA later in life.

© 2024 Fan J et al. *JAMA Network Open.*

eTable 2. Pairwise Genetic Correlation Estimation using LDSC (rg and p-value).

Trait1	Trait2	rg	se	P
JIA	SLE	0.610	0.097	2.75×10^{-10}
JIA	SSc	0.457	0.104	1.18×10^{-5}
RA	JIA	0.481	0.082	4.40×10^{-9}
RA	SLE	0.476	0.061	4.89×10^{-15}
RA	SSc	0.485	0.081	1.96×10^{-9}
SLE	SSc	0.841	0.109	9.38×10^{-15}

rg: genetic correlation

© 2024 Fan J et al. *JAMA Network Open.*

eTable 3. Genomic region with concordant associations among rheumatic diseases estimated by LOGODetect.

Chr	Start(GRCh37)	End(GRCh37)	Stat	pval	qval	Traits
1	114069670	114538653	66.828	2.00×10^{-4}	1.70×10^{-3}	JIA-RA
1	167404078	167453259	12.966	2.00×10^{-4}	1.70×10^{-3}	
2	65546879	65642200	-3.428	1.08×10^{-2}	3.89×10^{-2}	
2	100627660	100838157	5.713	2.00×10^{-4}	1.13×10^{-3}	
2	191161063	191973034	8.226	2.00×10^{-4}	1.13×10^{-3}	
2	204676238	204794496	7.464	2.00×10^{-4}	1.13×10^{-3}	
3	159710098	159744609	4.846	2.00×10^{-4}	2.80×10^{-3}	
3	188369449	188449052	3.300	6.00×10^{-3}	4.20×10^{-2}	
5	55410143	55567581	10.917	2.00×10^{-4}	1.00×10^{-3}	
5	150442829	150458604	1.781	6.20×10^{-3}	1.51×10^{-2}	
5	176755342	176800361	3.065	4.00×10^{-4}	1.50×10^{-3}	
10	6097165	6120424	15.650	2.00×10^{-4}	2.00×10^{-3}	
11	128495100	128504704	2.286	6.00×10^{-4}	5.40×10^{-3}	
14	21271573	21280895	4.699	3.60×10^{-3}	1.44×10^{-2}	
14	69225494	69311424	-8.982	2.00×10^{-4}	1.60×10^{-3}	
14	105903381	107101695	4.370	1.30×10^{-2}	3.47×10^{-2}	
15	58693661	58717919	1.547	1.10×10^{-2}	3.85×10^{-2}	
15	69992647	70044495	3.143	6.00×10^{-4}	4.20×10^{-3}	
16	28347140	29010771	13.343	1.80×10^{-3}	6.30×10^{-3}	
16	86002467	86018861	10.224	4.00×10^{-4}	2.80×10^{-3}	
18	12750758	12898548	23.119	2.00×10^{-4}	1.20×10^{-3}	
18	30269017	30333190	-6.702	1.34×10^{-2}	2.68×10^{-2}	
18	67497393	67561518	6.945	6.00×10^{-3}	1.80×10^{-2}	
19	10402131	10628548	22.692	2.00×10^{-4}	8.00×10^{-4}	
19	18270491	18424596	8.449	2.80×10^{-3}	5.60×10^{-3}	
21	36730471	36817791	5.897	5.80×10^{-3}	1.16×10^{-2}	
21	43737135	43880098	5.497	5.40×10^{-3}	1.16×10^{-2}	
22	21802961	21998833	16.733	2.00×10^{-4}	8.00×10^{-4}	
22	37528362	37562111	10.141	1.00×10^{-3}	1.33×10^{-3}	
22	39663185	39756270	10.188	8.00×10^{-4}	1.33×10^{-3}	
1	25282856	25309259	8.419	6.20×10^{-3}	3.51×10^{-2}	JIA-SLE
1	114071617	114651076	13.681	4.00×10^{-4}	3.40×10^{-3}	
1	167410391	167452610	12.513	2.00×10^{-4}	3.40×10^{-3}	
2	191851897	192003031	13.371	2.00×10^{-4}	3.40×10^{-3}	
2	214041359	214113781	2.348	8.20×10^{-3}	3.89×10^{-2}	
3	159481987	159747686	15.817	2.00×10^{-4}	2.80×10^{-3}	
5	55420280	55502476	2.858	2.00×10^{-4}	1.30×10^{-3}	
5	141826678	141843351	-1.581	4.60×10^{-3}	1.38×10^{-2}	

© 2024 Fan J et al. *JAMA Network Open.*

5	150433447	150461285	2.499	2.00×10^{-4}	1.30×10^{-3}	
5	159866993	159897501	1.705	1.40×10^{-3}	6.07×10^{-3}	
7	70961321	70986744	0.854	1.22×10^{-2}	4.47×10^{-2}	
7	128550030	128585104	4.647	2.00×10^{-4}	2.20×10^{-3}	
7	148332647	148358111	1.711	6.80×10^{-3}	3.74×10^{-2}	
11	511805	713912	3.212	6.00×10^{-4}	5.40×10^{-3}	
11	35162801	35213378	2.344	6.40×10^{-3}	2.88×10^{-2}	
11	128492373	128519816	2.224	1.18×10^{-2}	3.54×10^{-2}	
12	4005222	4331272	8.370	4.00×10^{-3}	2.43×10^{-2}	
12	56364321	56508821	6.951	5.40×10^{-3}	2.43×10^{-2}	
16	26719794	26734146	2.983	1.18×10^{-2}	3.15×10^{-2}	
16	85959438	86028237	6.760	2.00×10^{-4}	1.40×10^{-3}	
17	37828072	38125891	12.816	6.00×10^{-4}	1.80×10^{-3}	
17	73250311	73409693	10.478	6.00×10^{-4}	1.80×10^{-3}	
19	10393234	10630474	21.748	2.00×10^{-4}	8.00×10^{-4}	
19	18165602	18518778	9.206	1.40×10^{-3}	2.80×10^{-3}	
21	36531582	36673573	10.094	4.00×10^{-4}	1.60×10^{-3}	
2	100737715	100795783	6.078	5.60×10^{-3}	3.36×10^{-2}	
2	191293601	191989356	10.934	6.00×10^{-4}	1.02×10^{-2}	
8	61315720	61562268	9.554	2.00×10^{-4}	2.00×10^{-3}	
16	85964828	86032099	5.982	1.10×10^{-2}	3.85×10^{-2}	
17	37784990	38140524	11.012	1.20×10^{-3}	3.60×10^{-3}	JIA-SSc
17	73070788	73416771	8.235	8.00×10^{-4}	3.60×10^{-3}	
19	10432924	10471538	4.022	1.84×10^{-2}	2.45×10^{-2}	
19	18156090	18395776	4.195	1.80×10^{-3}	3.60×10^{-3}	
19	49945850	49955953	4.502	1.20×10^{-3}	3.60×10^{-3}	
1	38238903	38380285	1.740	2.48×10^{-2}	4.96×10^{-2}	
1	67737775	67828688	2.153	2.00×10^{-3}	7.20×10^{-3}	
1	92512218	92931263	1.923	1.88×10^{-2}	4.44×10^{-2}	
1	114140889	114537522	9.007	2.00×10^{-4}	1.13×10^{-3}	
1	161395972	161582255	-2.121	2.00×10^{-4}	1.13×10^{-3}	
1	167406510	167453780	2.102	5.80×10^{-3}	1.66×10^{-2}	
1	173183951	173372105	6.758	2.00×10^{-4}	1.13×10^{-3}	
1	234628809	234634952	1.732	2.00×10^{-2}	4.44×10^{-2}	RA-SLE
2	30437636	30500663	5.172	2.00×10^{-4}	1.70×10^{-3}	
2	65516234	65676583	4.971	1.00×10^{-3}	4.50×10^{-3}	
2	191511939	191568838	7.076	8.00×10^{-4}	4.50×10^{-3}	
2	191701210	191974435	24.793	2.00×10^{-4}	1.70×10^{-3}	
2	204593862	204754353	4.092	7.00×10^{-3}	2.22×10^{-2}	
5	1279790	1295349	-1.954	1.24×10^{-2}	3.10×10^{-2}	

© 2024 Fan J et al. *JAMA Network Open.*

5	133420643	133432632	2.502	1.40×10^{-3}	4.20×10^{-3}	
5	150428871	150466498	7.435	2.00×10^{-4}	1.00×10^{-3}	
5	159874235	159902313	2.723	4.00×10^{-4}	1.50×10^{-3}	
6	106538280	106613923	7.064	2.00×10^{-4}	1.10×10^{-3}	
6	138160065	138242638	17.083	2.00×10^{-4}	1.10×10^{-3}	
10	50013867	50148614	4.123	2.00×10^{-4}	1.00×10^{-3}	
10	63778606	63831928	9.906	2.00×10^{-4}	1.00×10^{-3}	
11	61543961	61619294	6.408	1.80×10^{-3}	5.40×10^{-3}	
11	118583002	118759610	14.463	2.00×10^{-4}	9.00×10^{-4}	
11	128488322	128507247	14.785	2.00×10^{-4}	9.00×10^{-4}	
12	24561938	24923805	2.343	1.20×10^{-3}	5.40×10^{-3}	
12	44035771	44109626	1.730	2.60×10^{-3}	7.80×10^{-3}	
12	56368078	56508821	-2.894	2.00×10^{-4}	1.80×10^{-3}	
12	92717914	92793867	1.806	1.16×10^{-2}	2.61×10^{-2}	
15	26041172	26063083	2.609	4.00×10^{-4}	1.40×10^{-3}	
15	38819889	38929338	4.378	2.00×10^{-4}	1.40×10^{-3}	
15	70033578	70043383	2.050	2.44×10^{-2}	4.88×10^{-2}	
17	37332036	38122686	16.398	2.00×10^{-4}	1.20×10^{-3}	
18	45640653	45680281	4.129	5.60×10^{-3}	1.68×10^{-2}	
18	46310355	46349795	3.930	8.20×10^{-3}	1.91×10^{-2}	
18	67495935	67568145	5.959	1.20×10^{-3}	7.20×10^{-3}	
19	10397131	10627814	13.842	2.00×10^{-4}	8.00×10^{-4}	
19	55735032	55783552	4.988	2.42×10^{-2}	4.03×10^{-2}	
22	21916166	22001704	18.279	2.00×10^{-4}	8.00×10^{-4}	
22	37544810	37558356	4.676	1.00×10^{-2}	1.33×10^{-2}	
22	39658958	39755773	7.114	4.00×10^{-4}	8.00×10^{-4}	
1	17650247	17687027	1.889	7.00×10^{-3}	2.38×10^{-2}	
1	38560968	38662594	2.312	1.20×10^{-3}	6.80×10^{-3}	
1	67779676	67828010	2.484	2.00×10^{-4}	1.70×10^{-3}	
1	85268243	85290497	1.444	1.18×10^{-2}	2.65×10^{-2}	
1	114125259	114395629	4.246	1.60×10^{-3}	6.80×10^{-3}	
1	167407686	167448392	1.754	1.00×10^{-2}	2.57×10^{-2}	
1	173219471	173375959	5.344	2.00×10^{-4}	1.70×10^{-3}	
2	30407284	30479927	1.504	2.00×10^{-4}	1.70×10^{-3}	RA-SSc
2	100761148	100782256	1.428	2.20×10^{-3}	9.88×10^{-3}	
2	121287412	121300205	0.812	1.78×10^{-2}	4.83×10^{-2}	
2	191512710	191558492	1.766	2.60×10^{-3}	9.88×10^{-3}	
2	191894141	191990778	3.887	2.00×10^{-4}	1.70×10^{-3}	
2	204595519	204694263	1.445	3.20×10^{-3}	1.01×10^{-2}	
3	12021902	12797563	7.044	9.00×10^{-3}	3.90×10^{-2}	

© 2024 Fan J et al. *JAMA Network Open.*

3	27996922	28097638	6.596	1.30×10^{-2}	3.90×10^{-2}	
3	58159893	58609930	7.741	3.20×10^{-3}	2.24×10^{-2}	
3	159710390	159743231	12.308	2.00×10^{-4}	2.80×10^{-3}	
3	159976271	160448598	6.161	1.20×10^{-2}	3.90×10^{-2}	
6	106559602	106604136	1.702	4.40×10^{-3}	1.76×10^{-2}	
6	138172383	138231107	4.222	2.00×10^{-4}	2.20×10^{-3}	
10	63785369	63808666	1.503	4.40×10^{-3}	4.40×10^{-2}	
11	60854841	60911440	0.726	9.40×10^{-3}	4.23×10^{-2}	
11	118575479	118655645	2.578	4.00×10^{-4}	3.60×10^{-3}	
14	49337432	49379880	-0.950	9.40×10^{-3}	3.76×10^{-2}	
14	75903728	75983051	1.270	4.60×10^{-3}	3.68×10^{-2}	
15	38822905	38921861	3.309	2.00×10^{-4}	7.00×10^{-4}	
15	70034088	70044495	-2.884	2.00×10^{-4}	7.00×10^{-4}	
19	10426422	10468097	2.482	2.58×10^{-2}	4.59×10^{-2}	
19	18167003	18392873	2.281	1.46×10^{-2}	4.59×10^{-2}	
19	52007416	52041103	2.354	3.44×10^{-2}	4.59×10^{-2}	
22	21916361	21980257	11.417	2.00×10^{-4}	4.00×10^{-4}	
22	39660829	39753003	10.580	2.00×10^{-4}	4.00×10^{-4}	
1	67781531	67831281	2.426	2.00×10^{-4}	1.70×10^{-3}	SLE-SSc
1	167407517	167445001	2.108	7.80×10^{-3}	4.42×10^{-2}	
1	173206060	173375997	7.232	2.00×10^{-4}	1.70×10^{-3}	
2	30407284	30479927	4.393	3.80×10^{-3}	2.28×10^{-2}	
2	191506773	191570072	9.249	6.00×10^{-4}	5.40×10^{-3}	
2	191887584	191985459	25.728	2.00×10^{-4}	3.40×10^{-3}	
3	58326220	58420418	4.522	1.00×10^{-2}	5.00×10^{-2}	
3	159481987	159608152	4.922	1.00×10^{-3}	7.50×10^{-3}	
3	159728987	159756451	11.716	2.00×10^{-4}	2.80×10^{-3}	
6	106549398	106597639	9.845	4.00×10^{-4}	2.20×10^{-3}	
6	138160722	138266431	14.302	2.00×10^{-4}	2.20×10^{-3}	
7	28133980	28203525	1.181	1.10×10^{-2}	4.03×10^{-2}	
7	70970374	70993604	0.908	1.04×10^{-2}	4.03×10^{-2}	
7	128568960	128743124	4.273	2.00×10^{-4}	2.20×10^{-3}	
8	9730774	12601971	8.720	6.60×10^{-3}	4.30×10^{-2}	
8	92956498	93088151	6.536	8.60×10^{-3}	4.30×10^{-2}	
11	552756	654147	14.426	2.00×10^{-4}	9.00×10^{-4}	
11	34587903	35132267	12.319	2.00×10^{-4}	9.00×10^{-4}	
11	118575180	119163319	8.208	1.22×10^{-2}	3.66×10^{-2}	
15	75034989	75609030	10.201	6.00×10^{-4}	4.20×10^{-3}	
16	31290557	31395578	24.619	2.00×10^{-4}	4.00×10^{-4}	
16	58250129	58290606	6.490	2.00×10^{-4}	4.00×10^{-4}	

© 2024 Fan J et al. *JAMA Network Open.*

16	85949473	86024734	19.606	2.00×10^{-4}	4.00×10^{-4}
17	37325271	38140524	11.677	2.80×10^{-3}	8.40×10^{-3}
17	73292911	73422219	12.281	2.00×10^{-4}	1.20×10^{-3}
17	77839025	77871667	5.834	1.98×10^{-2}	4.62×10^{-2}
20	6802370	6839006	-1.295	1.68×10^{-2}	4.20×10^{-2}
20	44667931	44742064	1.195	1.50×10^{-2}	4.20×10^{-2}
22	21798907	21973319	18.754	2.00×10^{-4}	8.00×10^{-4}
22	39667133	40036598	6.547	7.20×10^{-3}	1.44×10^{-2}

Chr: chromosome; Start and End positions are all positions on human genome build hg19; Stat: the scan statistic value, positive value means positive local genetic correlation between two traits and negative value means negative local correlation.

© 2024 Fan J et al. *JAMA Network Open.*

eTable 4. The genomic regions with shared effects among rheumatic diseases.

Chr	Start (GRCh37)	End (GRCh37)	PP.H0	PP.H1	PP.H2	PP.H3	PP.H4	Traits
1	114069670	114538653	6.99×10^{-157}	7.13×10^{-147}	1.37×10^{-13}	3.96×10^{-4}	1.00	
2	191161063	191973034	2.07×10^{-13}	1.48×10^{-8}	4.63×10^{-6}	3.30×10^{-1}	6.70×10^{-1}	
2	204676238	204794496	3.10×10^{-17}	1.92×10^{-17}	2.30×10^{-2}	1.33×10^{-2}	9.64×10^{-1}	
5	55410143	55567581	1.26×10^{-30}	8.87×10^{-17}	1.42×10^{-17}	8.99×10^{-7}	1.00	
10	6097165	6120424	5.35×10^{-12}	2.06×10^{-8}	1.32×10^{-4}	5.09×10^{-1}	4.90×10^{-1}	
14	69225494	69311424	4.28×10^{-6}	7.42×10^{-4}	3.90×10^{-4}	6.67×10^{-2}	9.32×10^{-1}	JIA-RA
16	86002467	86018861	1.14×10^{-5}	9.48×10^{-4}	7.89×10^{-4}	6.45×10^{-2}	9.34×10^{-1}	
18	12750758	12898548	2.68×10^{-9}	2.84×10^{-6}	5.88×10^{-4}	6.22×10^{-1}	3.77×10^{-1}	
19	10402131	10628548	6.60×10^{-17}	2.06×10^{-11}	2.71×10^{-8}	7.47×10^{-3}	9.93×10^{-1}	
22	21802961	21998833	1.34×10^{-3}	3.37×10^{-2}	1.07×10^{-2}	2.67×10^{-1}	6.87×10^{-1}	
22	37528362	37562111	1.62×10^{-1}	7.26×10^{-3}	2.73×10^{-1}	1.17×10^{-2}	5.46×10^{-1}	
1	114071617	114651076	7.92×10^{-22}	8.07×10^{-12}	1.76×10^{-15}	7.90×10^{-6}	9.99×10^{-1}	
1	167410391	167452610	4.01×10^{-6}	4.77×10^{-3}	1.17×10^{-7}	1.34×10^{-4}	5.09×10^{-1}	
2	191851897	192003031	1.23×10^{-67}	8.77×10^{-63}	1.09×10^{-9}	6.80×10^{-5}	9.93×10^{-1}	
2	214041359	214113781	3.75×10^{-3}	3.71×10^{-2}	1.19×10^{-2}	1.17×10^{-1}	8.30×10^{-1}	
3	159481987	159747686	7.13×10^{-6}	3.37×10^{-4}	1.24×10^{-3}	5.74×10^{-2}	9.41×10^{-1}	
5	55420280	55502476	9.26×10^{-16}	6.54×10^{-2}	1.16×10^{-16}	7.28×10^{-3}	9.27×10^{-1}	JIA-SLE
7	128550030	128585104	5.14×10^{-34}	8.41×10^{-33}	7.54×10^{-3}	1.23×10^{-1}	8.70×10^{-1}	
11	128492373	128519816	6.28×10^{-2}	3.62×10^{-3}	4.08×10^{-1}	2.30×10^{-2}	5.03×10^{-1}	
16	85959438	86028237	6.47×10^{-13}	5.73×10^{-11}	1.12×10^{-2}	9.88×10^{-1}	7.25×10^{-4}	
17	37828072	38125891	1.79×10^{-5}	2.79×10^{-3}	5.28×10^{-3}	8.24×10^{-1}	1.68×10^{-1}	
19	10393234	10630474	3.46×10^{-15}	1.17×10^{-9}	2.14×10^{-8}	6.26×10^{-3}	9.94×10^{-1}	
21	36531582	36673573	1.79×10^{-2}	2.43×10^{-1}	3.53×10^{-3}	4.71×10^{-2}	6.88×10^{-1}	
2	191293601	191989356	7.39×10^{-22}	4.50×10^{-17}	1.64×10^{-5}	9.99×10^{-1}	8.07×10^{-4}	
16	11355450	11478148	1.59×10^{-2}	2.33×10^{-1}	1.03×10^{-2}	1.50×10^{-1}	5.90×10^{-1}	JIA-SSc
16	85964828	86032099	3.51×10^{-11}	1.49×10^{-9}	2.30×10^{-2}	9.76×10^{-1}	7.72×10^{-4}	
17	37784990	38140524	7.26×10^{-6}	9.51×10^{-4}	6.59×10^{-4}	8.54×10^{-2}	9.13×10^{-1}	
1	67737775	67828688	2.68×10^{-4}	1.92×10^{-3}	2.20×10^{-3}	1.48×10^{-2}	9.81×10^{-1}	
1	92512218	92931263	1.74×10^{-2}	4.32×10^{-2}	2.93×10^{-2}	7.20×10^{-2}	8.38×10^{-1}	
1	114140889	114537522	3.62×10^{-153}	7.10×10^{-10}	8.03×10^{-147}	5.74×10^{-4}	9.99×10^{-1}	
1	161395972	161582255	2.90×10^{-11}	7.28×10^{-8}	8.88×10^{-6}	2.13×10^{-2}	9.79×10^{-1}	
1	173183951	173372105	4.73×10^{-14}	2.05×10^{-9}	2.28×10^{-5}	9.88×10^{-1}	1.15×10^{-2}	
2	30437636	30500663	2.01×10^{-3}	6.55×10^{-2}	5.81×10^{-4}	1.80×10^{-2}	9.14×10^{-1}	RA-SLE
2	65516234	65676583	3.60×10^{-10}	3.15×10^{-3}	1.10×10^{-7}	9.65×10^{-1}	3.19×10^{-2}	
2	191511939	191568838	2.13×10^{-15}	1.09×10^{-13}	4.52×10^{-3}	2.30×10^{-1}	7.65×10^{-1}	
2	191701210	191974435	1.55×10^{-66}	3.47×10^{-59}	1.43×10^{-8}	3.19×10^{-1}	6.81×10^{-1}	
5	133420643	133432632	1.75×10^{-6}	3.61×10^{-5}	1.83×10^{-2}	3.78×10^{-1}	6.04×10^{-1}	

© 2024 Fan J et al. *JAMA Network Open.*

5	150428871	150466498	5.12×10^{-14}	6.36×10^{-13}	2.84×10^{-2}	3.53×10^{-1}	6.19×10^{-1}	
6	138160065	138242638	5.24×10^{-24}	1.89×10^{-12}	2.49×10^{-14}	7.97×10^{-3}	9.92×10^{-1}	
10	50013867	50148614	7.26×10^{-6}	2.12×10^{-6}	1.07×10^{-1}	3.03×10^{-2}	8.63×10^{-1}	
10	63778606	63831928	2.05×10^{-10}	5.17×10^{-3}	3.90×10^{-8}	9.83×10^{-1}	1.15×10^{-2}	
11	118583002	118759610	7.29×10^{-13}	2.28×10^{-1}	1.84×10^{-13}	5.68×10^{-2}	7.15×10^{-1}	
11	128488322	128507247	5.51×10^{-5}	3.79×10^{-2}	3.34×10^{-4}	2.29×10^{-1}	7.32×10^{-1}	
15	38819889	38929338	4.52×10^{-9}	4.46×10^{-1}	1.58×10^{-10}	1.51×10^{-2}	5.39×10^{-1}	
15	70033578	70043383	3.35×10^{-9}	4.78×10^{-1}	6.70×10^{-11}	9.04×10^{-3}	5.13×10^{-1}	
17	37332036	38122686	6.44×10^{-9}	4.62×10^{-3}	1.30×10^{-6}	9.30×10^{-1}	6.55×10^{-2}	
19	10397131	10627814	2.47×10^{-16}	1.01×10^{-7}	1.52×10^{-9}	6.26×10^{-1}	3.74×10^{-1}	
22	21916166	22001704	7.03×10^{-11}	6.24×10^{-10}	1.27×10^{-2}	1.12×10^{-1}	8.76×10^{-1}	
1	38560968	38662594	2.47×10^{-4}	3.00×10^{-2}	4.19×10^{-5}	4.11×10^{-3}	9.66×10^{-1}	
1	67779676	67828010	2.08×10^{-5}	2.40×10^{-5}	1.70×10^{-1}	1.95×10^{-1}	6.35×10^{-1}	
1	173219471	173375959	6.77×10^{-10}	2.92×10^{-5}	2.01×10^{-6}	8.60×10^{-2}	9.14×10^{-1}	
2	100761148	100782256	5.03×10^{-7}	3.79×10^{-1}	3.76×10^{-8}	2.78×10^{-2}	5.93×10^{-1}	
2	191512710	191558492	2.67×10^{-7}	1.05×10^{-5}	5.28×10^{-4}	1.98×10^{-2}	9.80×10^{-1}	
2	191894141	191990778	1.48×10^{-26}	2.39×10^{-19}	3.29×10^{-10}	4.32×10^{-3}	9.96×10^{-1}	RA-SSc
3	58159893	58609930	1.45×10^{-6}	4.48×10^{-7}	1.07×10^{-1}	3.22×10^{-2}	8.61×10^{-1}	
6	106559602	106604136	1.59×10^{-1}	8.52×10^{-2}	6.64×10^{-2}	3.49×10^{-2}	6.54×10^{-1}	
6	138172383	138231107	5.88×10^{-13}	1.18×10^{-1}	1.67×10^{-12}	3.35×10^{-1}	5.47×10^{-1}	
11	118575479	118655645	4.11×10^{-19}	5.31×10^{-8}	2.03×10^{-13}	2.53×10^{-2}	9.75×10^{-1}	
22	21916361	21980257	1.70×10^{-3}	1.15×10^{-2}	8.17×10^{-3}	5.46×10^{-2}	9.24×10^{-1}	
22	39660829	39753003	3.23×10^{-4}	7.78×10^{-2}	2.78×10^{-3}	6.69×10^{-1}	2.50×10^{-1}	
1	67781531	67831281	5.26×10^{-6}	5.89×10^{-6}	4.29×10^{-2}	4.71×10^{-2}	9.10×10^{-1}	
1	173206060	173375997	2.95×10^{-12}	1.26×10^{-4}	8.76×10^{-9}	3.75×10^{-1}	6.25×10^{-1}	
2	191506773	191570072	2.19×10^{-16}	4.57×10^{-4}	4.63×10^{-13}	9.64×10^{-1}	3.59×10^{-2}	
2	191887584	191985459	5.36×10^{-75}	4.50×10^{-17}	1.19×10^{-58}	9.99×10^{-1}	1.01×10^{-3}	
3	58326220	58420418	2.21×10^{-8}	1.50×10^{-5}	1.36×10^{-3}	9.23×10^{-1}	7.55×10^{-2}	
3	159481987	159608152	4.64×10^{-4}	1.98×10^{-2}	8.14×10^{-4}	3.39×10^{-2}	9.45×10^{-1}	
3	159728987	159756451	2.64×10^{-8}	3.10×10^{-6}	3.35×10^{-4}	3.84×10^{-2}	9.61×10^{-1}	
6	106549398	106597639	7.37×10^{-8}	4.01×10^{-2}	2.93×10^{-8}	1.50×10^{-2}	9.45×10^{-1}	
6	138160722	138266431	1.28×10^{-11}	9.00×10^{-2}	3.69×10^{-11}	2.58×10^{-1}	6.52×10^{-1}	SLE-SSc
7	28133980	28203525	6.59×10^{-3}	6.23×10^{-4}	1.03×10^{-1}	8.82×10^{-3}	8.81×10^{-1}	
7	128568960	128743124	1.20×10^{-55}	1.47×10^{-17}	2.09×10^{-40}	2.45×10^{-2}	9.75×10^{-1}	
8	9730774	12601971	2.05×10^{-27}	1.64×10^{-17}	3.27×10^{-13}	1.62×10^{-3}	9.98×10^{-1}	
11	552756	654147	8.47×10^{-10}	2.24×10^{-4}	2.27×10^{-7}	5.92×10^{-2}	9.41×10^{-1}	
11	34587903	35132267	1.40×10^{-7}	5.99×10^{-2}	2.02×10^{-7}	8.56×10^{-2}	8.55×10^{-1}	
11	118575180	119163319	7.22×10^{-7}	1.70×10^{-7}	3.78×10^{-1}	8.85×10^{-2}	5.34×10^{-1}	
15	75034989	75609030	1.71×10^{-10}	2.89×10^{-10}	8.87×10^{-3}	1.41×10^{-2}	9.77×10^{-1}	
16	31290557	31395578	4.27×10^{-43}	2.09×10^{-3}	8.61×10^{-42}	4.12×10^{-2}	9.57×10^{-1}	

© 2024 Fan J et al. *JAMA Network Open.*

16	58250129	58290606	1.90×10^{-3}	6.79×10^{-3}	1.35×10^{-2}	4.75×10^{-2}	9.30×10^{-1}
16	85949473	86024734	4.72×10^{-21}	1.41×10^{-11}	3.10×10^{-12}	8.22×10^{-3}	9.92×10^{-1}
17	37325271	38140524	2.94×10^{-5}	8.83×10^{-3}	3.01×10^{-3}	9.04×10^{-1}	8.43×10^{-2}
17	73292911	73422219	6.50×10^{-4}	2.99×10^{-2}	3.37×10^{-3}	1.55×10^{-1}	8.11×10^{-1}
17	77839025	77871667	3.16×10^{-1}	6.92×10^{-2}	2.38×10^{-2}	4.62×10^{-3}	5.86×10^{-1}
22	21798907	21973319	1.92×10^{-10}	2.52×10^{-2}	9.26×10^{-10}	1.21×10^{-1}	8.54×10^{-1}
22	39667133	40036598	2.60×10^{-2}	4.21×10^{-3}	2.24×10^{-1}	3.57×10^{-2}	7.10×10^{-1}

© 2024 Fan J et al. *JAMA Network Open.*

eFigure 1. Tissue enrichment of genes in the genomic regions having shared associations with pairs of rheumatic diseases. P-values are shown on the y-axis with a scale of − log10. The bars in red represent tissues with significant enrichment surpassing Bonferroni adjustment threshold for multiple testing.

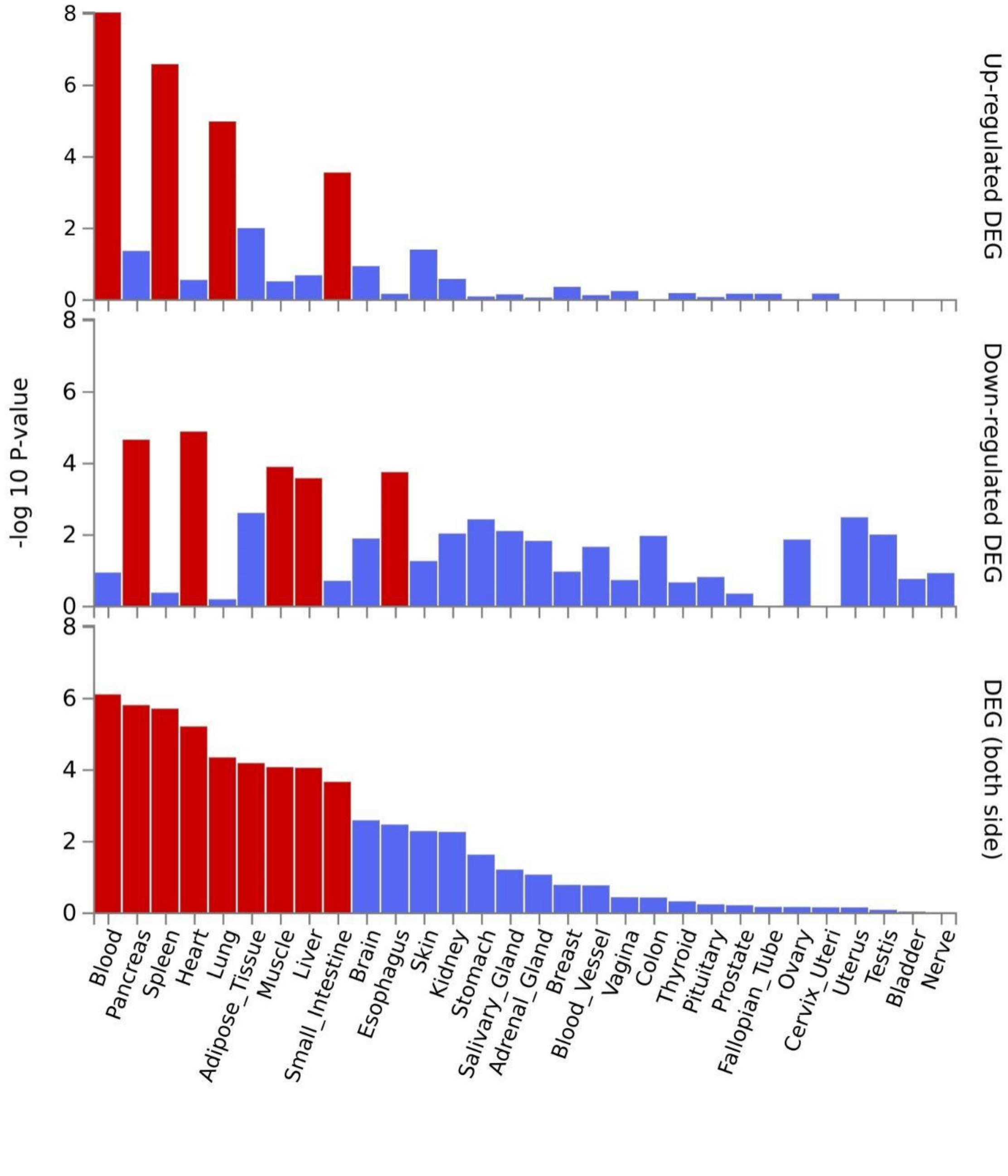

© 2024 Fan J et al. *JAMA Network Open.*

eFigure 2. Significantly enriched KEGG pathways for genes in the colocalized genetic regions associated with pairwise diseases.

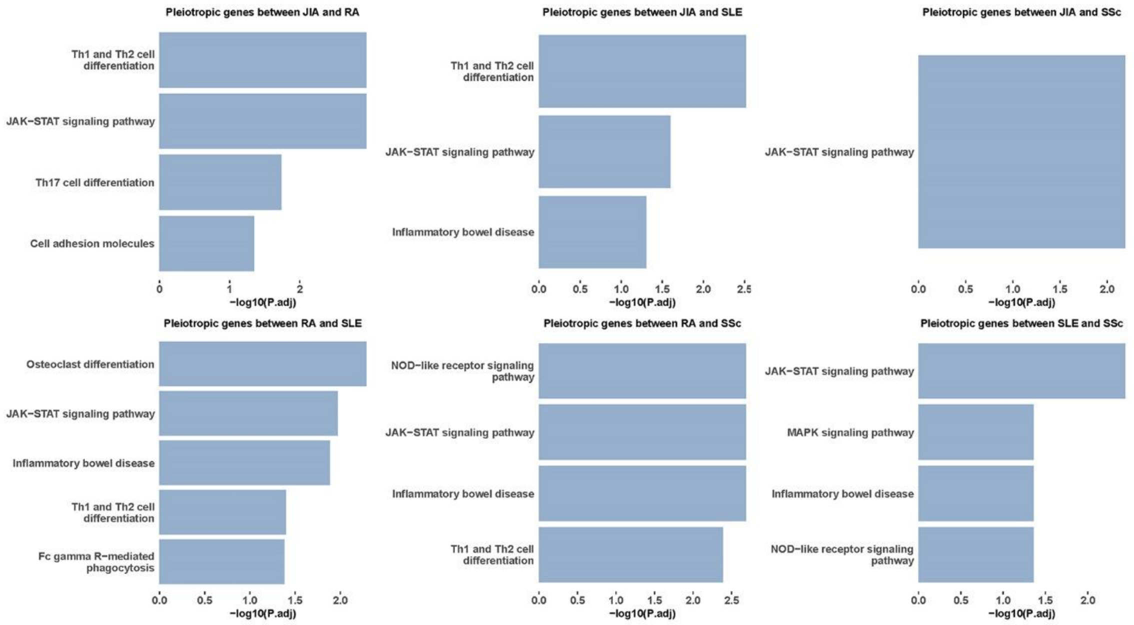

© 2024 Fan J et al. *JAMA Network Open.*

eTable 5. KEGG pathways enrichment for genes in the genomic regions with shared associations among rheumatic diseases.

ID	Description	GeneRatio	BgRatio	pvalue	p.adjust	geneID	Traits
hsa04658	Th1 and Th2 cell differentiation	5/31	92/8644	1.67×10^{-5}	1.08×10^{-3}	*IL2RA/IL2RB/STAT1/STAT4/TYK2*	JIA-RA
hsa04630	JAK-STAT signaling pathway	6/31	166/8644	2.27×10^{-5}	1.08×10^{-3}	*IL2RA/IL2RB/PTPN2/STAT1/STAT4/TYK2*	JIA-RA
hsa04659	Th17 cell differentiation	4/31	108/8644	5.59×10^{-4}	1.77×10^{-2}	*IL2RA/IL2RB/STAT1/TYK2*	JIA-RA
hsa05162	Measles	4/31	138/8644	1.40×10^{-3}	3.33×10^{-2}	*IL2RA/IL2RB/STAT1/TYK2*	JIA-RA
hsa04514	Cell adhesion molecules	4/31	158/8644	2.30×10^{-3}	4.37×10^{-2}	*CTLA4/ICAM1/ICAM3/ICOS*	JIA-RA
hsa04658	Th1 and Th2 cell differentiation	5/33	92/8644	2.30×10^{-5}	2.94×10^{-3}	*CD247/IL12A/STAT1/STAT4/TYK2*	JIA-SLE
hsa04630	JAK-STAT signaling pathway	5/33	166/8644	3.78×10^{-4}	2.37×10^{-2}	*CSF3/IL12A/STAT1/STAT4/TYK2*	JIA-SLE
hsa04620	Toll-like receptor signaling pathway	4/33	108/8644	7.13×10^{-4}	2.37×10^{-2}	*IL12A/IRF5/STAT1/TYK2*	JIA-SLE
hsa05144	Malaria	3/33	50/8644	8.79×10^{-4}	2.37×10^{-2}	*CSF3/ICAM1/IL12A*	JIA-SLE
hsa05169	Epstein-Barr virus infection	5/33	202/8644	9.24×10^{-4}	2.37×10^{-2}	*CD247/ICAM1/PSMD3/STAT1/TYK2*	JIA-SLE
hsa05321	Inflammatory bowel disease	3/33	65/8644	1.88×10^{-3}	4.02×10^{-2}	*IL12A/STAT1/STAT4*	JIA-SLE
hsa04630	JAK-STAT signaling pathway	4/13	166/8644	8.19×10^{-5}	6.23×10^{-3}	*CSF3/SOCS1/STAT1/STAT4*	JIA-SSc
hsa05140	Leishmaniasis	5/48	77/8644	6.25×10^{-5}	6.65×10^{-3}	*FCGR2A/FCGR2C/FCGR3A/FCGR3B/STAT1*	RA-SLE
hsa04380	Osteoclast differentiation	6/48	135/8644	9.30×10^{-5}	6.65×10^{-3}	*FCGR2A/FCGR2C/FCGR3A/FCGR3B/STAT1/TYK2*	RA-SLE
hsa05150	Staphylococcus aureus infection	5/48	96/8644	1.79×10^{-4}	8.51×10^{-3}	*FCGR2A/FCGR2C/FCGR3A/FCGR3B/ICAM1*	RA-SLE
hsa04630	JAK-STAT signaling pathway	6/48	166/8644	2.88×10^{-4}	1.03×10^{-2}	*CSF3/IL12RB2/IL23R/STAT1/STAT4/TYK2*	RA-SLE
hsa05321	Inflammatory bowel disease	4/48	65/8644	4.42×10^{-4}	1.26×10^{-2}	*IL12RB2/IL23R/STAT1/STAT4*	RA-SLE

© 2024 Fan J et al. *JAMA Network Open.*

hsa04658	Th1 and Th2 cell differentiation	4/48	92/8644	1.64×10^{-3}	3.90×10^{-2}	*IL12RB2/STAT1/STAT4/TYK2*	RA-SLE
hsa04666	Fc gamma R-mediated phagocytosis	4/48	97/8644	1.99×10^{-3}	4.06×10^{-2}	*ACTR2/FCGR2A/FCGR3A/FCGR3B*	RA-SLE
hsa04630	JAK-STAT signaling pathway	4/12	166/8644	5.76×10^{-5}	1.97×10^{-3}	*IL12RB2/PDGFB/STAT1/STAT4*	RA-SSc
hsa05321	Inflammatory bowel disease	3/12	65/8644	8.51×10^{-5}	1.97×10^{-3}	*IL12RB2/STAT1/STAT4*	RA-SSc
hsa04621	NOD-like receptor signaling pathway	4/12	186/8644	8.98×10^{-5}	1.97×10^{-3}	*ATG5/STAT1/TAB1/TNFAIP3*	RA-SSc
hsa04658	Th1 and Th2 cell differentiation	3/12	92/8644	2.39×10^{-4}	3.95×10^{-3}	*IL12RB2/STAT1/STAT4*	RA-SSc
hsa04120	Ubiquitin mediated proteolysis	3/12	142/8644	8.57×10^{-4}	1.13×10^{-2}	*FANCL/PPIL2/UBE2L3*	RA-SSc
hsa04217	Necroptosis	3/12	159/8644	1.19×10^{-3}	1.18×10^{-2}	*STAT1/STAT4/TNFAIP3*	RA-SSc
hsa05161	Hepatitis B	3/12	162/8644	1.26×10^{-3}	1.18×10^{-2}	*STAT1/STAT4/TAB1*	RA-SSc
hsa05169	Epstein-Barr virus infection	3/12	202/8644	2.37×10^{-3}	1.95×10^{-2}	*STAT1/TAB1/TNFAIP3*	RA-SSc
hsa05140	Leishmaniasis	2/12	77/8644	4.88×10^{-3}	3.58×10^{-2}	*STAT1/TAB1*	RA-SSc
hsa04630	JAK-STAT signaling pathway	8/61	166/8644	1.94×10^{-5}	3.89×10^{-3}	*CSF3/GRB2/HRAS/IL12A/IL12RB2/PDGFB/STAT1/STAT4*	SLE-SSc
hsa05161	Hepatitis B	7/61	162/8644	1.33×10^{-4}	1.34×10^{-2}	*ATF4/GRB2/HRAS/IRF7/STAT1/STAT4/TAB1*	SLE-SSc
hsa05215	Prostate cancer	5/61	97/8644	5.81×10^{-4}	3.89×10^{-2}	*ATF4/ERBB2/GRB2/HRAS/PDGFB*	SLE-SSc
hsa05321	Inflammatory bowel disease	4/61	65/8644	1.10×10^{-3}	4.68×10^{-2}	*IL12A/IL12RB2/STAT1/STAT4*	SLE-SSc
hsa04010	MAPK signaling pathway	8/61	301/8644	1.16×10^{-3}	4.68×10^{-2}	*ATF4/CACNA1I/CACNB1/ERBB2/GRB2/HRAS/PDGFB/TAB1*	SLE-SSc
hsa04621	NOD-like receptor signaling pathway	6/61	186/8644	1.80×10^{-3}	4.98×10^{-2}	*ATG5/CTSB/IRF7/STAT1/TAB1/TNFAIP3*	SLE-SSc

© 2024 Fan J et al. *JAMA Network Open.*

eFigure 3. Manhattan plots summarizing results from bivariate MTAG analyses between JIA and adult rheumatic diseases. The SNPs reaching genome-wide significant threshold ($P < 5x10^{-8}$) were indicated by yellow. Lead SNPs at novel loci were indicated by red. Results from bivariate MTAG analyses between JIA and RA (A), JIA and SLE (B), JIA and SSc (C), SSc and SLE (D).

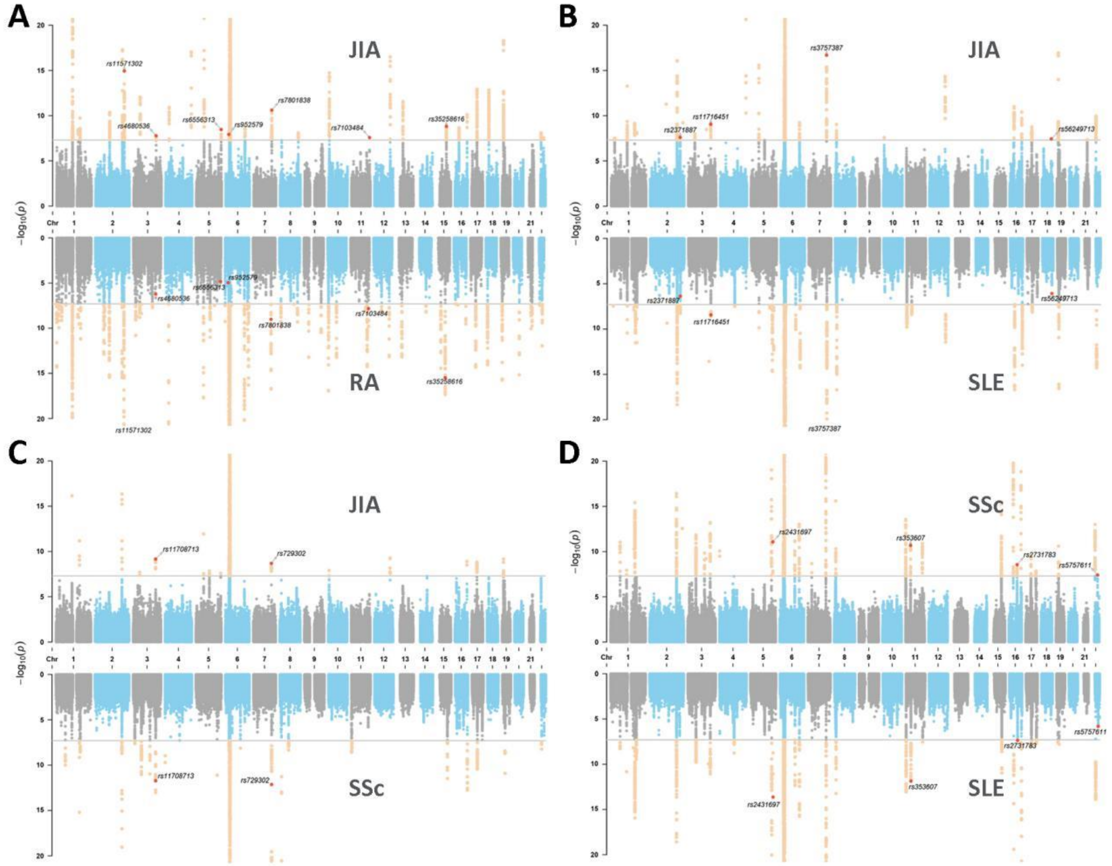

© 2024 Fan J et al. *JAMA Network Open.*

eFigure 4. Regional association plots of the 7 novel JIA loci from bivariate MTAG between JIA and RA.

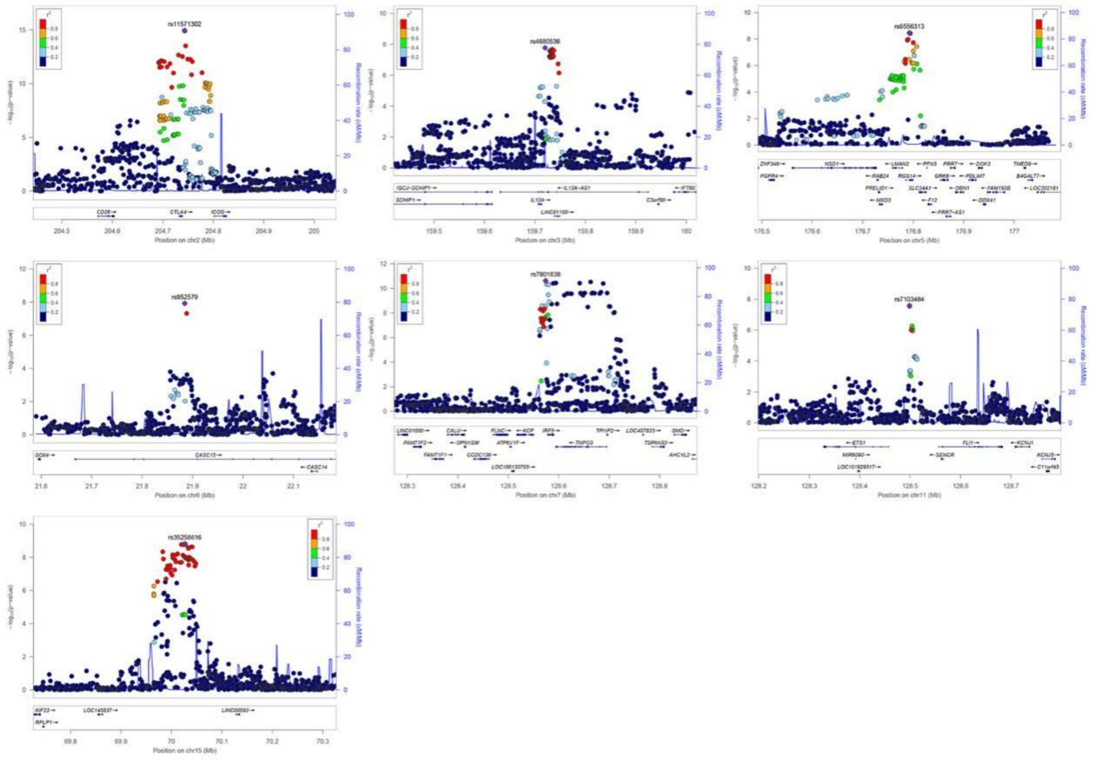

© 2024 Fan J et al. *JAMA Network Open.*

eFigure 5. Regional association plots of the 4 novel JIA loci from bivariate MTAG between JIA and SLE.

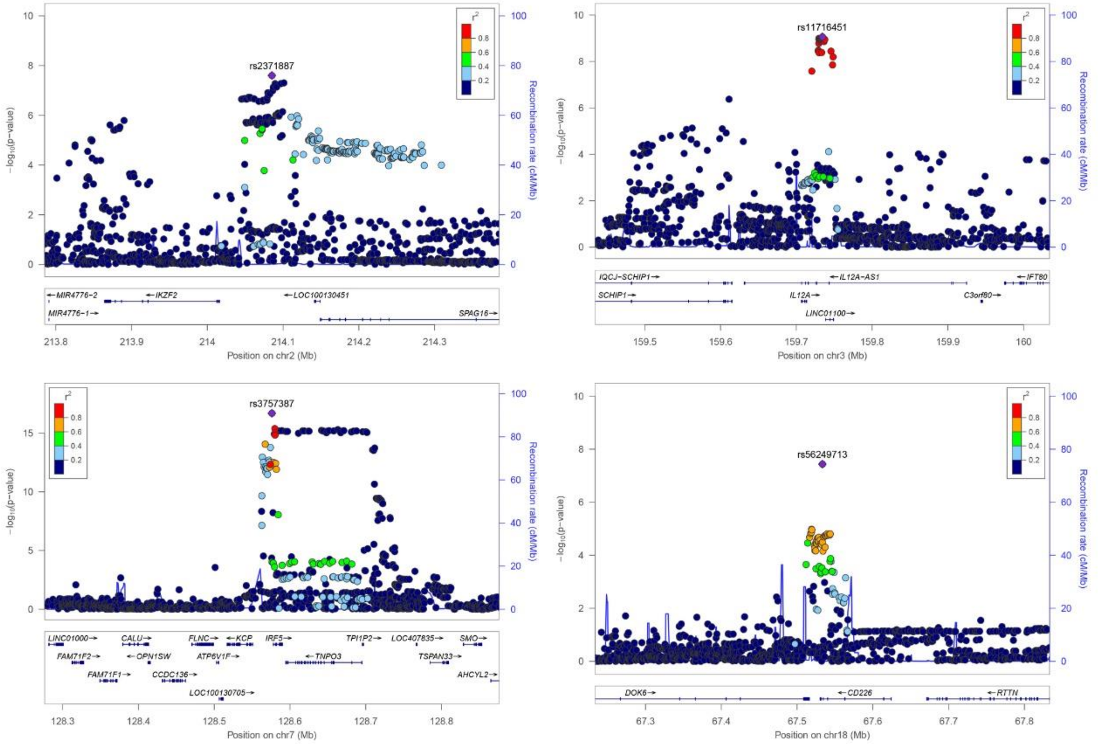

© 2024 Fan J et al. *JAMA Network Open.*

eFigure 6. Regional association plots of the 2 novel JIA loci from bivariate MTAG between JIA and SSc.

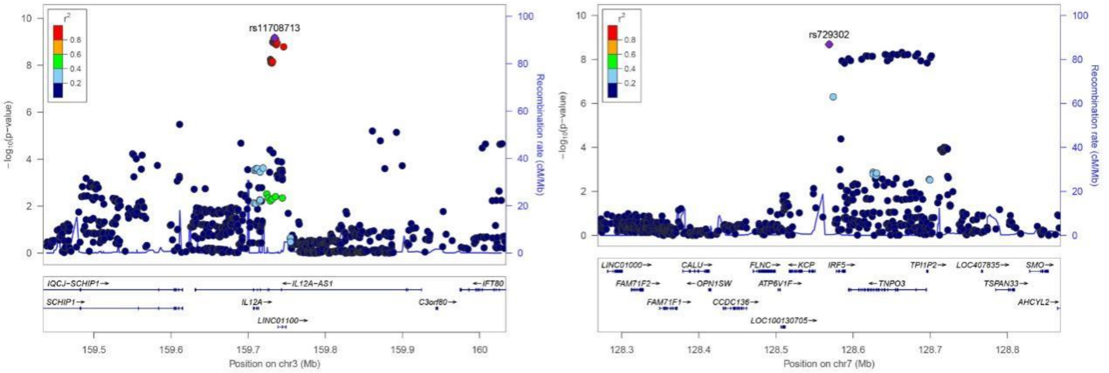

© 2024 Fan J et al. *JAMA Network Open.*

eFigure 7. Regional association plots of the 4 novel SSc loci from bivariate MTAG between SSc and SLE.

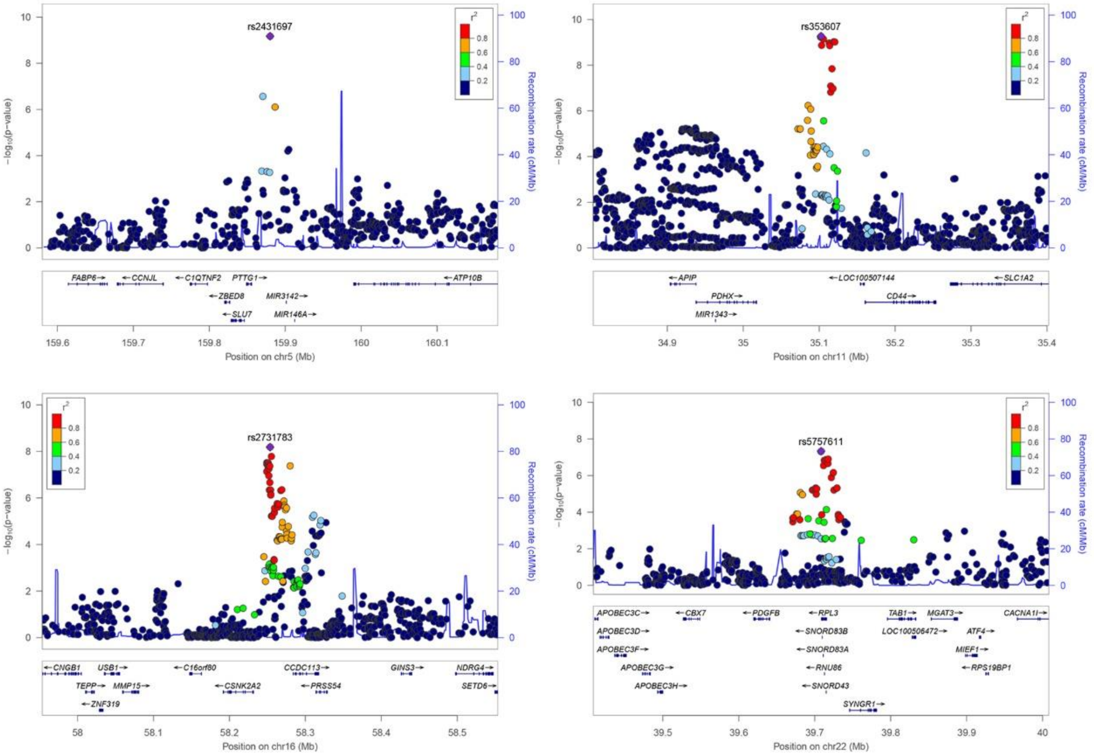

© 2024 Fan J et al. *JAMA Network Open.*

eTable 6. Lead SNPs (P < 5×10^{-8} and r2 < 0.1) from MTAG analyses.

a. Lead SNPs of JIA from MTAG analysis on disease-pair JIA and RA

SNP	CHR	BP (GRCh37)	A1	FRQ	MTAG_beta	P.MTAG	JIA_beta	P.JIA	RA_beta	P.RA	Nearest Gene	Novel?
rs36064592	1	114462662	A	0.330	-0.049	1.65×10^{-8}	-0.093	4.86×10^{-4}	-0.094	1.40×10^{-7}	*PTPN22*	No
rs9651036	1	154360492	A	0.104	0.079	3.58×10^{-9}	0.211	1.03×10^{-7}	0.086	2.00×10^{-3}	*ATP8B2*	No
rs840012	1	167414872	T	0.405	-0.053	1.81×10^{-10}	-0.148	5.62×10^{-9}	-0.051	3.50×10^{-3}	*POU2F1*	No
rs4851252	2	100757280	T	0.356	-0.056	4.34×10^{-11}	-0.103	6.43×10^{-5}	-0.094	1.70×10^{-8}	*AFF3*	No
rs7574865	2	191964633	T	0.228	0.084	5.14×10^{-18}	0.188	1.04×10^{-10}	0.122	7.10×10^{-12}	*STAT4*	No
**rs11571302**	**2**	**204742934**	**T**	**0.471**	**-0.065**	1.14×10^{-15}	**-0.101**	4.58×10^{-5}	**-0.139**	2.90×10^{-18}	***CD28***	**Yes**
rs11574435	3	46447972	T	0.120	-0.090	8.73×10^{-13}	-0.232	2.48×10^{-9}	-0.117	6.60×10^{-6}	*CCR5*	No
**rs4680536**	**3**	**159720271**	**A**	**0.571**	**0.046**	1.68×10^{-8}	**0.111**	8.85×10^{-6}	**0.058**	3.70×10^{-4}	***IL12A-AS1***	**Yes**
rs34185821	4	26085480	A	0.697	-0.060	1.26×10^{-11}	-0.070	9.70×10^{-3}	-0.139	2.20×10^{-16}	*RBPJ*	No
rs7731626	5	55444683	A	0.365	-0.103	2.29×10^{-34}	-0.266	6.90×10^{-21}	-0.198	7.90×10^{-23}	*IL6ST*	No
**rs6556313**	**5**	**176792491**	**A**	**0.663**	**-0.051**	3.41×10^{-9}	**-0.138**	1.38×10^{-7}	**-0.051**	2.70×10^{-3}	***RGS14***	**Yes**
**rs952579**	**6**	**21884440**	**A**	**0.154**	**0.064**	1.19×10^{-8}	**0.175**	8.62×10^{-7}	**0.086**	2.80×10^{-3}	***CASC15***	**Yes**
rs13190937	6	28411244	A	0.334	-0.097	3.68×10^{-29}	-0.216	9.83×10^{-17}	-0.128	2.70×10^{-13}	*GPX6*	No
rs1002658	6	137981584	T	0.182	-0.064	1.55×10^{-9}	-0.143	8.61×10^{-6}	-0.094	2.20×10^{-5}	*LOC100130476*	No
**rs7801838**	**7**	**128573994**	**T**	**0.295**	**-0.060**	2.40×10^{-11}	**-0.138**	3.12×10^{-7}	**-0.083**	4.80×10^{-6}	***IRF5***	**Yes**
rs1516971	8	129542100	T	0.872	0.070	8.27×10^{-9}	0.140	1.56×10^{-4}	0.122	5.30×10^{-7}	*LINC00824*	No
rs706778	10	6098949	T	0.410	0.066	1.83×10^{-15}	0.144	7.54×10^{-9}	0.104	7.10×10^{-12}	*IL2RA*	No
**rs7103484**	**11**	**128498760**	**A**	**0.789**	**-0.055**	2.63×10^{-8}	**-0.118**	1.02×10^{-4}	**-0.094**	5.10×10^{-6}	***FLI1***	**Yes**
rs7137828	12	111932800	T	0.513	-0.069	3.12×10^{-17}	-0.165	2.95×10^{-11}	-0.083	5.30×10^{-7}	*FAM109A*	No
rs9603608	13	40318819	A	0.661	0.060	2.77×10^{-12}	0.120	3.82×10^{-6}	0.104	7.60×10^{-11}	*MIR4305*	No
**rs35258616**	**15**	**70026514**	**T**	**0.100**	**-0.082**	1.53×10^{-9}	**-0.118**	4.28×10^{-3}	**-0.186**	6.40×10^{-12}	***TLE3***	**Yes**
rs9933582	16	86016026	T	0.767	-0.063	7.63×10^{-11}	-0.145	1.90×10^{-6}	-0.105	1.10×10^{-7}	*IRF8*	No
rs35736272	17	38032680	T	0.529	-0.060	1.18×10^{-13}	-0.123	6.40×10^{-7}	-0.094	4.20×10^{-9}	*ZPBP2*	No
rs559406	18	12857002	T	0.541	-0.060	1.44×10^{-13}	-0.132	7.59×10^{-8}	-0.083	3.30×10^{-7}	*SEH1L*	No
rs2278442	19	10444826	A	0.654	0.055	1.38×10^{-10}	0.116	1.73×10^{-5}	0.095	2.30×10^{-8}	*ICAM5*	No
rs5998509	22	21917479	T	0.191	0.060	8.09×10^{-9}	0.138	2.09×10^{-5}	0.086	1.70×10^{-5}	*UBE2L3*	No
rs2069235	22	39747780	A	0.299	0.049	2.85×10^{-8}	0.078	3.88×10^{-3}	0.104	3.00×10^{-10}	*TAB1*	No

b. Lead SNPs of JIA from MTAG analysis on disease-pair JIA and SLE

© 2024 Fan J et al. *JAMA Network Open.*

SNP	CHR	BP (GRCh37)	A1	FRQ	MTAG_beta	P.MTAG	JIA_beta	P.JIA	SLE_beta	P.SLE	Nearest Gene	Novel?
rs6679677	1	114303808	A	0.104	0.143	1.15×10^{-23}	0.341	5.93×10^{-17}	0.336	4.55×10^{-13}	*PHTF1*	No
rs1214598	1	167426424	A	0.375	-0.055	7.30×10^{-10}	-0.147	1.21×10^{-8}	-0.094	2.15×10^{-3}	*CD247*	No
rs7574865	2	191964633	T	0.228	0.123	1.53×10^{-32}	0.188	1.04×10^{-10}	0.560	9.80×10^{-66}	*STAT4*	No
**rs2371887**	**2**	**214085179**	**A**	**0.555**	**-0.049**	**2.49×10^{-8}**	**-0.117**	**3.72×10^{-6}**	**-0.122**	**4.48×10^{-5}**	***IKZF2***	**Yes**
**rs11716451**	**3**	**159734349**	**A**	**0.583**	**0.054**	**8.61×10^{-10}**	**0.121**	**1.04×10^{-6}**	**0.151**	**6.78×10^{-7}**	***IL12A-AS1***	**Yes**
rs7731626	5	55444683	A	0.365	-0.088	3.59×10^{-22}	-0.266	6.90×10^{-21}	-0.117	1.84×10^{-4}	*ANKRD55*	No
rs9393830	6	27587027	T	0.372	0.065	4.03×10^{-13}	0.142	2.52×10^{-8}	0.191	1.48×10^{-10}	*ZNF184*	No
rs2614258	6	135677202	A	0.384	0.055	5.88×10^{-10}	0.143	2.06×10^{-8}	0.104	7.38×10^{-4}	*AHI1*	No
**rs3757387**	**7**	**128576086**	**T**	**0.540**	**-0.074**	**2.07×10^{-17}**	**-0.107**	**1.57×10^{-5}**	**-0.372**	**1.12×10^{-38}**	***IRF5***	**Yes**
rs4840568	8	11351019	A	0.265	0.060	9.20×10^{-10}	0.096	6.65×10^{-4}	0.270	5.18×10^{-18}	*BLK*	No
rs597808	12	111973358	A	0.484	0.068	4.72×10^{-15}	0.165	4.32×10^{-11}	0.163	3.51×10^{-8}	*ATXN2*	No
rs2280381	16	86018633	T	0.625	0.059	3.95×10^{-11}	0.145	3.79×10^{-8}	0.151	1.91×10^{-6}	*LOC124903741*	No
rs8067378	17	38051348	A	0.485	-0.052	1.73×10^{-9}	-0.123	6.41×10^{-7}	-0.128	8.92×10^{-6}	*EPDR1*	No
**rs56249713**	**18**	**67533332**	**T**	**0.587**	**0.049**	**3.66×10^{-8}**	**0.121**	**4.25×10^{-6}**	**0.117**	**8.45×10^{-5}**	***CD226***	**Yes**
rs2304256	19	10475652	A	0.283	-0.083	1.14×10^{-17}	-0.186	1.17×10^{-11}	-0.236	1.54×10^{-12}	*TYK2*	No
rs909121	21	36659684	T	0.278	0.053	4.53×10^{-8}	0.138	2.09×10^{-6}	0.113	5.31×10^{-4}	*RUNX1*	No
rs140492	22	21923144	A	0.806	-0.071	1.08×10^{-10}	-0.136	1.91×10^{-5}	-0.247	1.24×10^{-13}	*UBE2L3*	No

c. Lead SNPs of JIA from MTAG analysis on disease-pair JIA and SSc

SNP	CHR	BP(GRCh37)	A1	FRQ	MTAG_beta	P.MTAG	JIA_beta	P.JIA	SSc_beta	P.SSc	Nearest Gene	Novel?
rs2056626	1	167420425	T	0.586	0.064	6.79×10^{-12}	0.133	8.66×10^{-8}	0.212	1.31×10^{-11}	*CD247*	No
rs4853458	2	191959489	A	0.228	0.091	4.34×10^{-17}	0.187	1.23×10^{-10}	0.298	4.86×10^{-18}	*STAT4*	No
**rs11708713**	**3**	**159734286**	**A**	**0.417**	**-0.057**	**6.80×10^{-10}**	**-0.121**	**1.03×10^{-6}**	**-0.120**	**7.07×10^{-9}**	***IL12A-AS1***	**Yes**
rs7748520	6	27864908	T	0.965	0.162	6.90×10^{-11}	0.428	1.15×10^{-9}	0.600	2.42×10^{-3}	*OR2B2*	No
**rs729302**	**7**	**128568960**	**A**	**0.680**	**0.059**	**2.07×10^{-9}**	**0.121**	**3.82×10^{-6}**	**0.154**	**1.41×10^{-9}**	***IRF5***	**Yes**
rs2280381	16	86018633	T	0.625	0.057	1.29×10^{-9}	0.145	3.79×10^{-8}	0.102	7.78×10^{-4}	*LOC124903741*	No
rs4795397	17	38023745	A	0.522	-0.055	1.49×10^{-9}	-0.127	2.47×10^{-7}	-0.100	7.92×10^{-6}	*ZPBP2*	No

d. Lead SNPs of SSc from MTAG analysis on disease-pair SSc and SLE

SNP	CHR	BP(GRCh37)	ALT	FRQ	MTAG_beta	P.MTAG	SSc_beta	P.SSc	SLE_beta	P.SLE	Nearest Gene	Novel?
rs3790566	1	67814440	T	0.244	0.055	9.14×10^{-12}	0.152	3.84×10^{-10}	0.131	1.19×10^{-4}	*IL12RB2*	No
rs10776773	1	114220738	G	0.275	-0.043	3.54×10^{-8}	-0.111	4.51×10^{-6}	-0.117	1.69×10^{-4}	*LRIG2*	No

© 2024 Fan J et al. *JAMA Network Open.*

rs2056626	1	167420425	G	0.390	-0.047	5.18 x 10^{-11}	-0.212	1.31 x 10^{-11}	-0.083	7.09 x 10^{-3}	*POU2F1*	No
rs1857066	1	173332629	A	0.255	-0.063	2.97 x 10^{-15}	-0.139	5.02 x 10^{-9}	-0.211	1.90 x 10^{-10}	*TNFSF4*	No
rs4853458	2	191959489	A	0.231	0.131	5.44 x 10^{-57}	0.298	4.86 x 10^{-18}	0.560	9.88 x 10^{-66}	*STAT4*	No
rs4681845	3	58332496	T	0.261	0.056	1.42 x 10^{-12}	0.149	1.61 x 10^{-10}	0.131	3.41 x 10^{-5}	*PDHB*	No
rs9884090	3	119116150	A	0.155	-0.061	1.45 x 10^{-10}	-0.181	1.89 x 10^{-10}	-0.105	3.14 x 10^{-3}	*POGLUT1*	No
rs485499	3	159745863	C	0.349	-0.055	6.09 x 10^{-14}	-0.170	3.29 x 10^{-10}	-0.151	4.06 x 10^{-7}	*IL12A-AS1*	No
rs11724804	4	965779	A	0.441	0.045	8.73 x 10^{-11}	0.155	5.31 x 10^{-11}	0.077	4.63 x 10^{-3}	*DGKQ*	No
rs3792783	5	150455732	G	0.158	0.086	9.50 x 10^{-20}	0.186	2.42 x 10^{-12}	0.262	3.20 x 10^{-12}	*TNIP1*	No
**rs2431697**	**5**	**159879978**	**C**	**0.431**	**-0.048**	**8.51 x 10^{-12}**	**-0.110**	**4.29 x 10^{-4}**	**-0.223**	**2.60 x 10^{-14}**	***PTTG1***	**Yes**
rs1012894	6	106597639	G	0.393	0.049	6.86 x 10^{-12}	0.109	2.73 x 10^{-5}	0.199	1.46 x 10^{-11}	*PRDM1*	No
rs5029924	6	138187498	T	0.024	0.169	9.51 x 10^{-14}	0.334	5.71 x 10^{-5}	0.548	8.02 x 10^{-16}	*TNFAIP3*	No
rs849139	7	28179396	C	0.389	0.043	2.11 x 10^{-9}	0.124	2.60 x 10^{-7}	0.113	1.15 x 10^{-4}	*JAZF1*	No
rs13238352	7	128647942	T	0.100	0.171	5.84 x 10^{-50}	0.338	3.52 x 10^{-21}	0.588	2.33 x 10^{-45}	*TNPO3*	No
rs2736340	8	11343973	T	0.244	0.094	3.44 x 10^{-31}	0.217	3.33 x 10^{-21}	0.262	2.14 x 10^{-16}	*BLK*	No
rs6598008	11	618172	A	0.440	-0.053	2.74 x 10^{-14}	-0.221	1.97 x 10^{-8}	-0.174	6.74 x 10^{-10}	*RASSF7*	No
**rs353607**	**11**	**35102366**	**T**	**0.452**	**-0.047**	**2.04 x 10^{-11}**	**-0.084**	**4.62 x 10^{-5}**	**-0.186**	**3.37 x 10^{-11}**	***CD44***	**Yes**
rs74676029	11	118628373	T	0.201	-0.059	1.22 x 10^{-11}	-0.170	3.73 x 10^{-11}	-0.117	1.01 x 10^{-3}	*TREH*	No
rs1378942	15	75077367	C	0.387	0.058	5.45 x 10^{-16}	0.165	1.84 x 10^{-14}	0.131	2.00 x 10^{-5}	*CSK*	No
rs9940397	16	31303201	C	0.141	0.114	3.03 x 10^{-30}	0.150	1.98 x 10^{-6}	0.548	2.10 x 10^{-46}	*PYCARD*	No
**rs2731783**	**16**	**58253460**	**A**	**0.140**	**0.059**	**2.81 x 10^{-9}**	**0.140**	**1.22 x 10^{-6}**	**0.157**	**2.94 x 10^{-5}**	***CCDC113***	**Yes**
rs11644034	16	85972612	A	0.194	-0.089	2.56 x 10^{-24}	-0.212	1.12 x 10^{-14}	-0.288	1.78 x 10^{-15}	*IRF8*	No
rs2952144	17	37960017	T	0.484	-0.044	2.58 x 10^{-10}	-0.114	1.20 x 10^{-7}	-0.117	1.99 x 10^{-5}	*GRB7*	No
rs7207506	17	73409410	T	0.172	-0.052	1.48 x 10^{-8}	-0.135	2.28 x 10^{-5}	-0.174	6.27 x 10^{-6}	*GRB2*	No
rs2305743	19	18193191	A	0.199	-0.058	2.50 x 10^{-11}	-0.187	4.64 x 10^{-10}	-0.128	2.91 x 10^{-4}	*MAST3*	No
rs5754344	22	21963786	G	0.182	0.067	9.95 x 10^{-14}	0.123	1.75 x 10^{-6}	0.239	2.41 x 10^{-12}	*UBE2L3*	No
**rs5757611**	**22**	**39708357**	**T**	**0.204**	**0.047**	**3.89 x 10^{-8}**	**0.136**	**1.01 x 10^{-6}**	**0.113**	**9.22 x 10^{-4}**	***SYNGR1***	**Yes**

© 2024 Fan J et al. *JAMA Network Open.*

eFigure 8. Conditional analyses on the three JIA novel loci and one SSc novel locus from MTAG analysis. Locus-zoom plots of the novel JIA locus emerged from all three MTAG analyses and the novel SSc locus (on the left of each panel). The independent association signals at each locus are shown on the right of each panel.

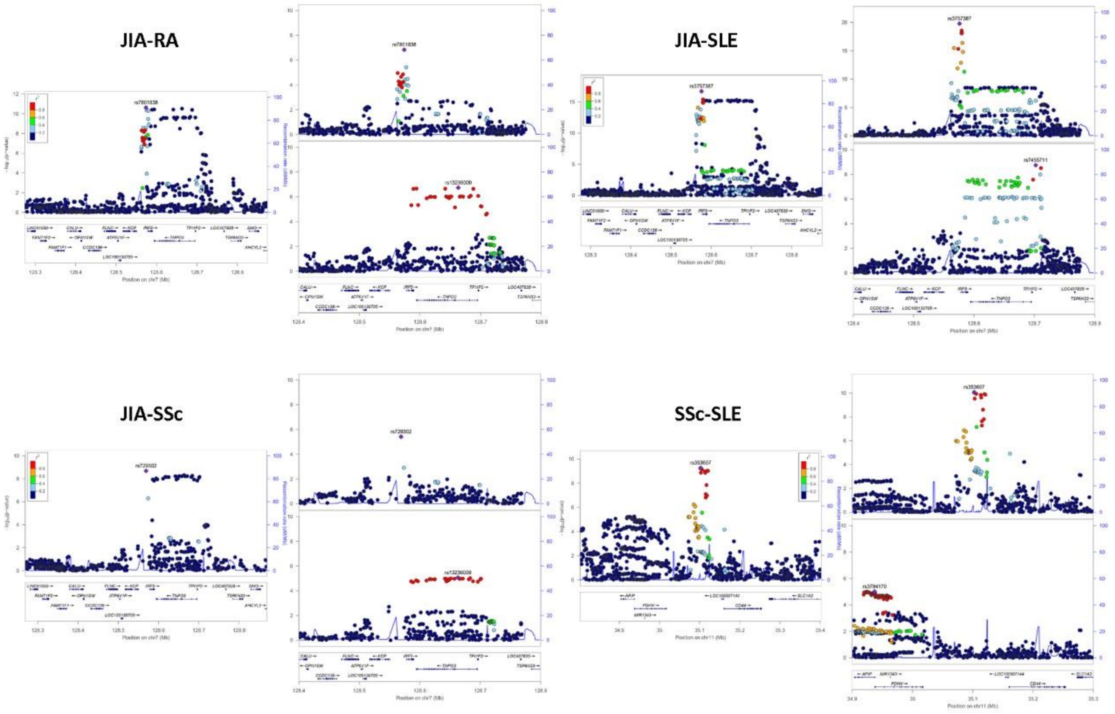

© 2024 Fan J et al. *JAMA Network Open.*

eTable 7. Conditional analysis yielded additional association signals at the novel loci for JIA and SSc.

a. Additional association signal from conditional analysis for novel JIA locus on chr7 from MTAG of JIA-RA.

Chr	SNP	bp(GRCh37)	A1	b	se	p	bJ	bJ_se	pJ	LD_r
7	rs7801838	128573994	T	-0.060	0.009	2.40×10^{-11}	-0.049	0.009	7.48×10^{-8}	0.214
7	rs13236009	128663173	T	-0.084	0.013	3.03×10^{-11}	-0.069	0.013	9.44×10^{-8}	0.000

b. Additional association signal from conditional analysis for novel JIA locus on chr7 from MTAG of JIA-SLE.

Chr	SNP	bp(GRCh37)	A1	b	se	p	bJ	bJ_se	pJ	LD_r
7	rs3757387	128576086	T	-0.074	0.009	2.07×10^{-17}	-0.084	0.009	3.98×10^{-21}	-0.179
7	rs7455711	128702426	A	-0.041	0.009	6.78×10^{-6}	-0.057	0.009	9.79×10^{-10}	0.000

c. Additional association signal from conditional analysis for novel JIA locus on chr7 from MTAG of JIA-SSc

Chr	SNP	bp(GRCh37)	A1	b	se	p	bJ	bJ_se	pJ	LD_r
7	rs729302	128568960	A	0.059	0.010	2.07×10^{-9}	0.048	0.010	2.01×10^{-6}	-0.234
7	rs13236009	128663173	T	-0.083	0.014	4.83×10^{-9}	-0.067	0.015	4.72×10^{-6}	0.000

d. Additional association signal from conditional analysis for novel SSc locus on chr11 from MTAG of SSc-SLE

Chr	SNP	bp(GRCh37)	A1	b	se	p	bJ	bJ_se	pJ	LD_r
11	rs3794170	34937138	C	0.051	0.011	2.37×10^{-6}	0.048	0.011	9.47×10^{-6}	-0.044
11	rs353607	35102366	T	-0.047	0.007	2.04×10^{-11}	-0.045	0.007	8.15×10^{-11}	0.000

© 2024 Fan J et al. *JAMA Network Open.*

eFigure 9. Circos plots of chromatin interactions and eQTLs at the 7 novel JIA loci from bivariate MTAG between JIA and RA. Links colored orange indicate chromatin interactions and those colored in green represent eQTLs. When a gene was mapped by both, it was colored red. The GWAS loci were highlighted in blue in the second layer.

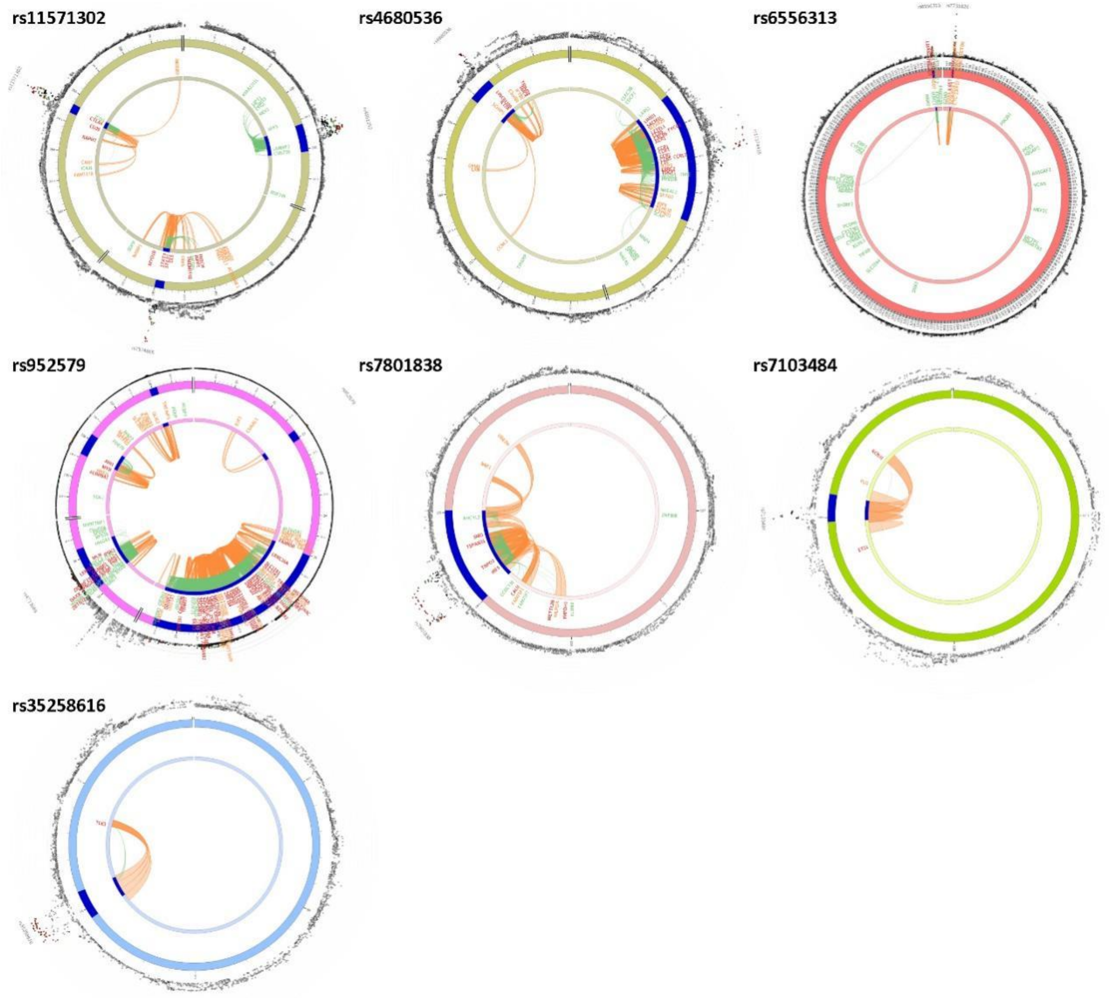

© 2024 Fan J et al. *JAMA Network Open.*

eFigure 10. Circos plots of chromatin interactions and eQTLs at the 4 novel JIA loci from bivariate MTAG between JIA and SLE. Links colored orange indicate chromatin interactions and those colored in green represent eQTLs. When a gene was mapped by both, it was colored red. The GWAS loci were highlighted in blue in the second layer.

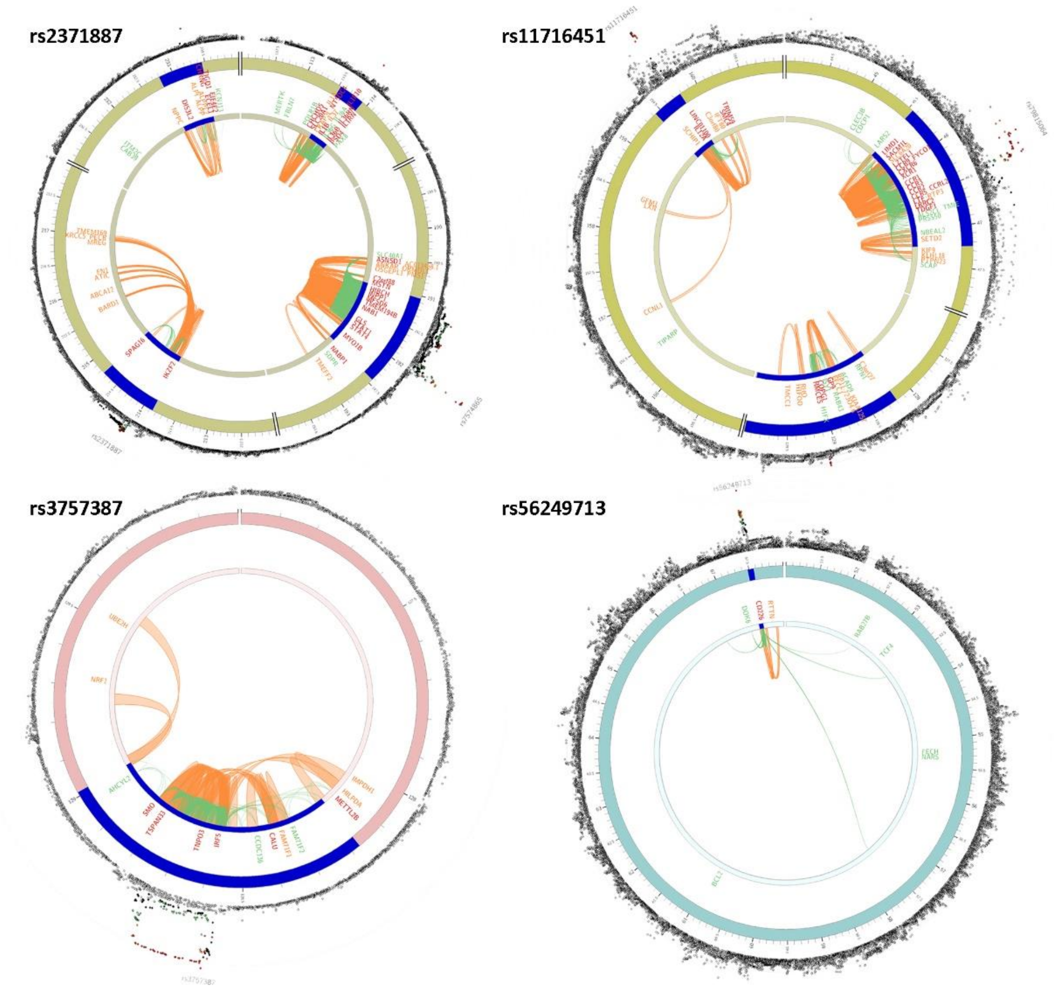

© 2024 Fan J et al. *JAMA Network Open.*

eFigure 11. Circos plots of chromatin interactions and eQTLs at the 2 novel JIA loci from bivariate MTAG between JIA and SSc.Links colored orange indicate chromatin interactions and those colored in green represent eQTLs. When a gene was mapped by both, it was colored red. The GWAS loci were highlighted in blue in the second layer.

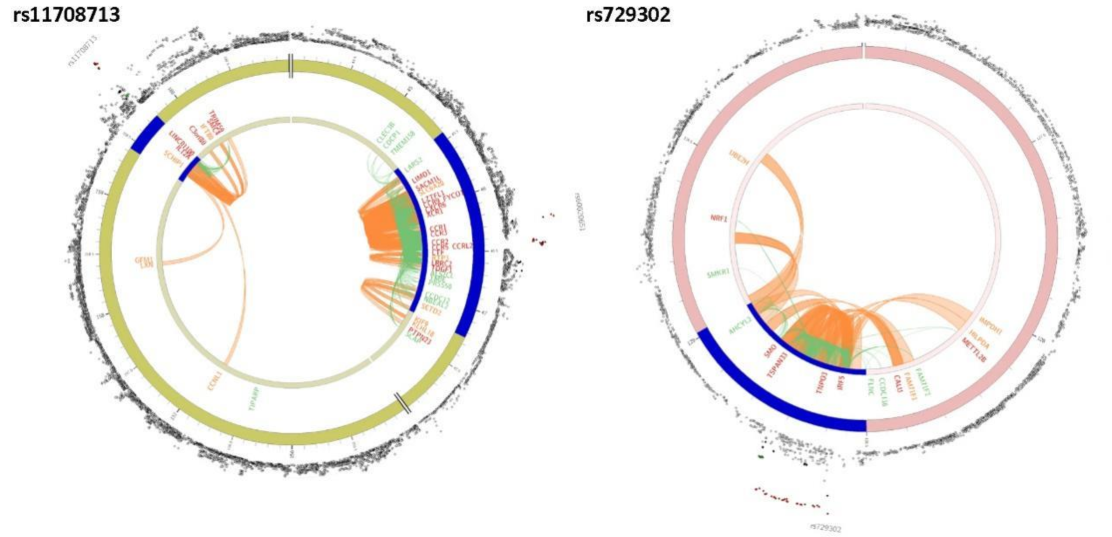

eFigure 12. Circos plots of chromatin interactions and eQTLs at the 4 novel SSc loci from bivariate MTAG between SSc and SLE. Links colored orange indicate chromatin interactions and those colored in green represent eQTLs. When a gene was mapped by both, it was colored red. The GWAS loci were highlighted in blue in the second layer.

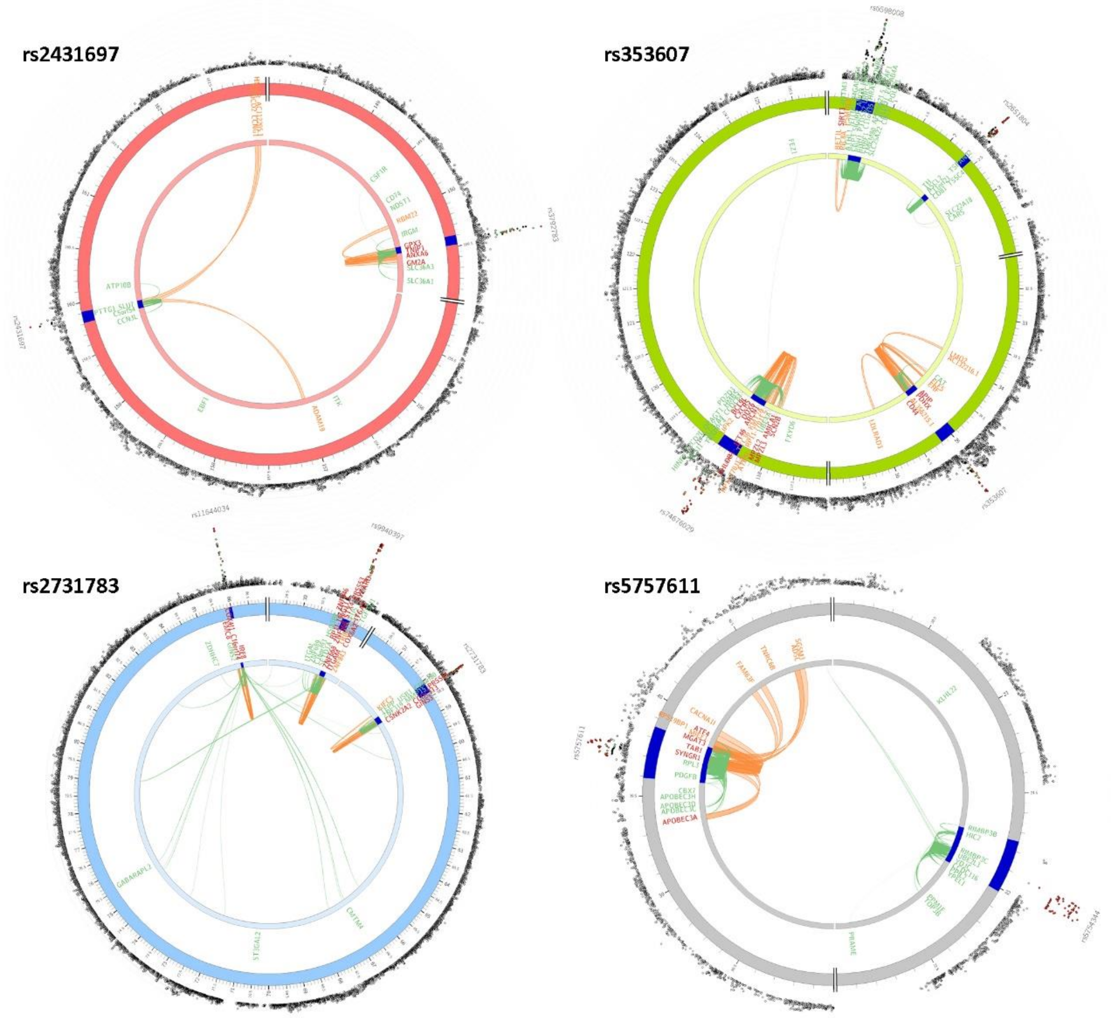

© 2024 Fan J et al. *JAMA Network Open.*

eTable 8. Loci With Opposite Directions of Effect

SNP	chr	Bp (GRCh37)	A1	P-asset	OR.1	OR. 2	Subset. 1	Subset .2	Nearest Gene
rs1801274	1	161479745	A	1.73×10^{-17}	1.09	0.83	RA	SLE	*FCGR2A*
rs2393997	6	27670697	T	1.12×10^{-10}	1.13	0.81	RA	SLE	*LINC010 112*
rs17457484	13	42952499	T	3.00×10^{-10}	1.08	0.89	RA,SLE, SSc	JIA	*FABP3P 2*
rs2236262	14	69261472	A	1.09×10^{-10}	1.14	0.94	JIA	RA	*ZFP36L1*
rs9074	20	44688665	A	2.56×10^{-13}	1.10	0.89	SLE,SS c	RA	*SLC12A5*

© 2024 Fan J et al. *JAMA Network Open.*

eFigure 13. The five independent loci with effects in opposite directions across the four diseases. The radius of each wedge corresponds to the -log10(Pvalue) obtained from original GWAS summary statistics of the SNP for four disorders. The color indicates whether the examined SNP confers risk (red) or protective (green) effects for each disorder. The dotted line represents the suggestive significance threshold (P = 1 x 10^{-5}). The position of each SNP on human genome build GRCh37 is indicated in the figure.

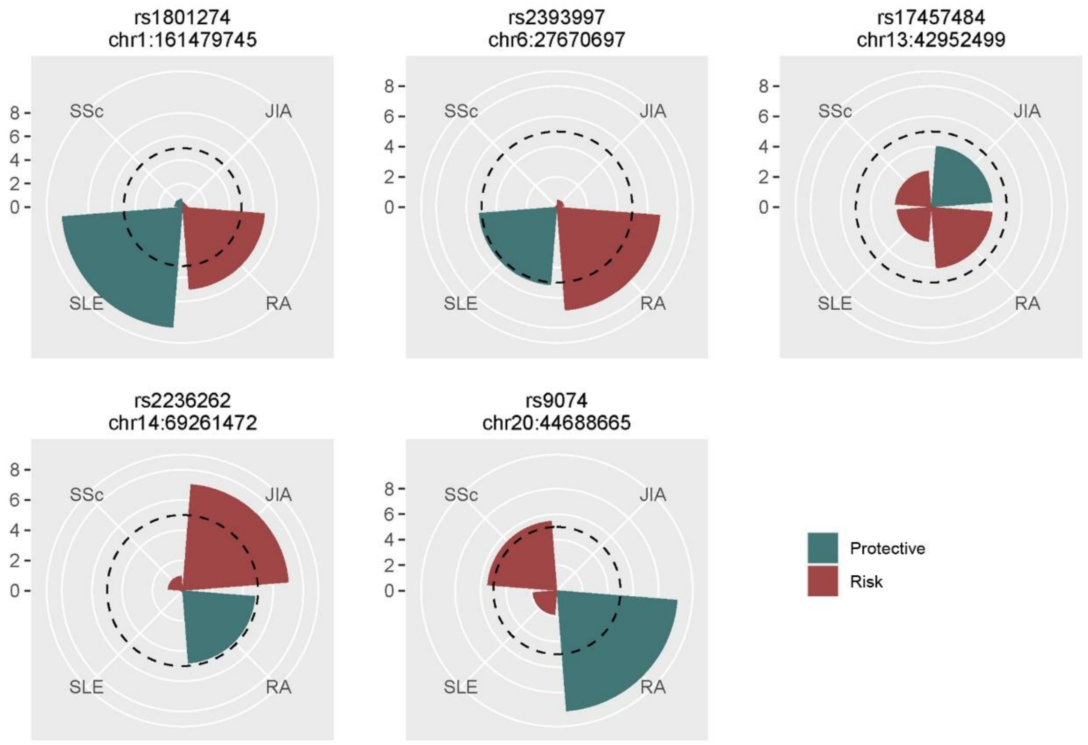

© 2024 Fan J et al. *JAMA Network Open.*

eTable 9. Candidate genes identified by SMR with FDR < 0.05.

a. SMR JIA

probeID	ProbeChr	Gene	Probe_bp	p_SMR	p_HEIDI	nsnp_HEIDI	Database
ILMN_1676924	1	*CD247*	167400008	1.69×10^{-7}	0.126	20	CAGE
ILMN_2086143	3	*CCR4*	32996340	5.35×10^{-5}	0.332	20	CAGE
ILMN_1743145	5	*ERAP2*	96249089	8.25×10^{-8}	0.618	20	CAGE
ILMN_1814737	5	*LNPEP*	96364618	1.37×10^{-6}	0.161	20	CAGE
ILMN_1875342	5	*AK124143*	96372005	2.66×10^{-6}	0.188	20	CAGE
ILMN_1671703	10	*ACTA2*	90694953	5.30×10^{-5}	0.156	20	CAGE
ILMN_1667519	11	*RRAS2*	14300848	1.62×10^{-4}	0.233	20	CAGE
ILMN_1678490	12	*RILPL2*	123900360	1.09×10^{-4}	0.084	20	CAGE
ILMN_1669281	16	*CLN3*	28488860	2.14×10^{-5}	0.099	20	CAGE
ILMN_1701477	16	*CCDC101*	28602159	1.01×10^{-7}	0.327	20	CAGE
ILMN_1684789	16	*CCDC101*	28603040	3.75×10^{-8}	0.262	20	CAGE
ILMN_2336133	16	*SULT1A1*	28617525	2.80×10^{-5}	0.179	20	CAGE
ILMN_1657302	16	*SULT1A1*	28634462	3.85×10^{-6}	0.391	20	CAGE
ILMN_2300695	17	*IKZF3*	37921755	1.84×10^{-6}	0.104	20	CAGE
ILMN_1666206	17	*GSDMB*	38060997	9.32×10^{-7}	0.239	20	CAGE
ILMN_2347193	17	*GSDMB*	38062136	1.08×10^{-6}	0.170	20	CAGE
ILMN_2260756	17	*GSDMB*	38073727	3.65×10^{-5}	0.256	20	CAGE
ILMN_1662174	17	*ORMDL3*	38077572	1.03×10^{-6}	0.180	20	CAGE
ILMN_1676955	19	*TYK2*	10461262	6.70×10^{-8}	0.098	20	CAGE
ILMN_1668369	19	*CDC37*	10501885	1.04×10^{-4}	0.111	20	CAGE
ILMN_2158003	19	*KIAA1683*	18367952	1.70×10^{-5}	0.249	20	CAGE
ENSG00000198821	1	*CD247*	167443862	2.52×10^{-8}	0.231	20	eQTLGen
ENSG00000114988	2	*LMAN2L*	97388733	6.21×10^{-5}	0.728	20	eQTLGen
ENSG00000183813	3	*CCR4*	32995453	1.65×10^{-6}	0.383	20	eQTLGen
ENSG00000138688	4	*KIAA1109*	123178700	3.75×10^{-5}	0.222	20	eQTLGen
ENSG00000164308	5	*ERAP2*	96233531	6.65×10^{-8}	0.399	20	eQTLGen
ENSG00000113441	5	*LNPEP*	96322158	7.19×10^{-7}	0.744	20	eQTLGen
ENSG00000179428	7	*IL6-AS1*	22766126	3.50×10^{-5}	0.208	20	eQTLGen
ENSG00000107796	10	*ACTA2*	90722989	4.20×10^{-5}	0.299	20	eQTLGen
ENSG00000026103	10	*FAS*	90762978	9.69×10^{-5}	0.164	20	eQTLGen
ENSG00000188603	16	*CLN3*	28492439	5.06×10^{-8}	0.207	20	eQTLGen
ENSG00000176476	16	*SGF29*	28584173	1.43×10^{-8}	0.786	20	eQTLGen
ENSG00000161405	17	*IKZF3*	37970819	1.06×10^{-6}	0.173	20	eQTLGen

© 2024 Fan J et al. *JAMA Network Open.*

ENSG00000073605	17	*GSDMB*	38068477	7.36×10^{-7}	0.333	20	eQTLGen
ENSG00000172057	17	*ORMDL3*	38080574	7.42×10^{-7}	0.208	20	eQTLGen
ENSG00000105397	19	*TYK2*	10476280	5.09×10^{-11}	0.207	20	eQTLGen
ENSG00000130518	19	*IQCN*	18376612	1.27×10^{-5}	0.397	20	eQTLGen
ENSG00000134352	5	*IL6ST*	55230923	3.70×10^{-7}	0.332	7	GTEx
ENSG00000164308	5	*ERAP2*	96211643	7.61×10^{-8}	0.928	20	GTEx
ENSG00000113441	5	*LNPEP*	96271098	3.10×10^{-5}	0.327	20	GTEx
ENSG00000146109	6	*ABT1*	26597180	4.75×10^{-4}	0.127	20	GTEx
ENSG00000181315	6	*ZNF322*	26636518	1.80×10^{-4}	0.168	20	GTEx
ENSG00000135541	6	*AHI1*	135604670	1.86×10^{-5}	0.054	20	GTEx
ENSG00000107796	10	*ACTA2*	90694831	8.44×10^{-5}	0.149	20	GTEx
ENSG00000233232	16	*NPIPB7*	28467693	2.87×10^{-5}	0.061	20	GTEx
ENSG00000188603	16	*CLN3*	28477983	4.30×10^{-6}	0.135	20	GTEx
ENSG00000176476	16	*CCDC101*	28565236	2.93×10^{-7}	0.208	20	GTEx
ENSG00000197165	16	*SULT1A2*	28603264	5.38×10^{-6}	0.116	20	GTEx
ENSG00000196502	16	*SULT1A1*	28616903	1.95×10^{-5}	0.093	20	GTEx
ENSG00000161405	17	*IKZF3*	37921198	5.20×10^{-5}	0.307	20	GTEx
ENSG00000073605	17	*GSDMB*	38060848	1.44×10^{-6}	0.467	20	GTEx
ENSG00000172057	17	*ORMDL3*	38077294	2.04×10^{-6}	0.499	20	GTEx
ENSG00000125457	17	*MIF4GD*	73262309	4.53×10^{-4}	0.897	20	GTEx
ENSG00000130518	19	*KIAA1683*	18367908	5.09×10^{-5}	0.369	20	GTEx

b.SMR RA

probeID	ProbeChr	Gene	Probe_bp	p_SMR	p_HEIDI	nsnp_HEIDI	Database
ILMN_1761921	1	*IL12RB2*	67862175	1.38×10^{-4}	0.283	5	CAGE
ILMN_1657754	1	*RPAP2*	92853524	9.95×10^{-5}	0.103	20	CAGE
ILMN_1691693	1	*FCRH3*	157646477	2.91×10^{-6}	0.623	20	CAGE
ILMN_1797428	1	*FCRL3*	157648068	3.08×10^{-6}	0.421	20	CAGE
ILMN_1699599	1	*FCRL3*	157648482	3.55×10^{-6}	0.559	20	CAGE
ILMN_1791329	1	*FCRL2*	157715845	3.47×10^{-4}	0.087	20	CAGE
ILMN_1766085	2	*REL*	61149525	3.87×10^{-6}	0.989	20	CAGE
ILMN_2154115	2	*PSD4*	113960411	6.97×10^{-5}	0.296	20	CAGE
ILMN_1799604	4	*OCIAD1*	48862844	2.89×10^{-4}	0.124	20	CAGE
ILMN_1814622	5	*GIN1*	102432382	2.65×10^{-4}	0.097	20	CAGE
ILMN_2333774	6	*TAGAP*	159460207	1.76×10^{-4}	0.109	16	CAGE
ILMN_1869943	7	*AK022839*	27138323	1.72×10^{-4}	0.283	20	CAGE

© 2024 Fan J et al. *JAMA Network Open.*

ILMN_1687213	8	*FAM167A*	11279135	2.77×10^{-7}	0.185	20	CAGE
ILMN_1746819	9	*C5*	123714740	1.58×10^{-4}	0.116	20	CAGE
ILMN_1754538	10	*C10orf58*	82191879	1.36×10^{-4}	0.323	20	CAGE
ILMN_1873620	10	*TSPAN14*	82281079	2.89×10^{-4}	0.104	20	CAGE
ILMN_1786759	11	*C11orf10*	61557309	2.26×10^{-4}	0.062	20	CAGE
ILMN_1670134	11	*FADS1*	61567437	5.05×10^{-7}	0.722	20	CAGE
ILMN_2075065	11	*FADS2*	61633933	1.10×10^{-6}	0.486	20	CAGE
ILMN_2176955	11	*CUL5*	107978157	1.24×10^{-5}	0.196	20	CAGE
ILMN_1725079	12	*TSPAN31*	58141897	3.32×10^{-4}	0.299	20	CAGE
ILMN_1723846	12	*METTL21B*	58176212	4.49×10^{-6}	0.078	20	CAGE
ILMN_2097954	12	*TSFM*	58190221	1.74×10^{-4}	0.061	20	CAGE
ILMN_1688098	13	*TBC1D4*	75859374	8.90×10^{-5}	0.618	20	CAGE
ILMN_1738369	16	*TUFM*	28854373	1.60×10^{-4}	0.135	20	CAGE
ILMN_1681016	16	*SPNS1*	28995825	5.38×10^{-5}	0.871	20	CAGE
ILMN_1805636	17	*PGAP3*	37827537	1.76×10^{-6}	0.077	20	CAGE
ILMN_1679476	21	*GART*	34876491	2.09×10^{-4}	0.109	20	CAGE
ILMN_1810875	22	*SYNGR1*	39772125	8.41×10^{-9}	0.473	20	CAGE
ILMN_1721712	22	*SYNGR1*	39774214	7.12×10^{-10}	0.097	20	CAGE
ILMN_1727805	22	*SYNGR1*	39781479	5.77×10^{-9}	0.389	20	CAGE
ENSG00000204084	1	*INPP5B*	38369549	2.36×10^{-4}	0.194	20	eQTLGen
ENSG00000122484	1	*RPAP2*	92809126	1.73×10^{-4}	0.409	20	eQTLGen
ENSG00000162676	1	*GFI1*	92946376	1.56×10^{-4}	0.426	20	eQTLGen
ENSG00000170989	1	*S1PR1*	101704759	2.89×10^{-4}	0.420	20	eQTLGen
ENSG00000160856	1	*FCRL3*	157658459	2.32×10^{-6}	0.321	20	eQTLGen
ENSG00000152284	2	*TCF7L1*	85449022	2.22×10^{-4}	0.810	20	eQTLGen
ENSG00000144218	2	*AFF3*	100461459	5.79×10^{-9}	0.207	20	eQTLGen
ENSG00000125637	2	*PSD4*	113937858	4.76×10^{-5}	0.368	20	eQTLGen
ENSG00000115896	2	*PLCL1*	199053365	1.50×10^{-4}	0.080	20	eQTLGen
ENSG00000233252	3	*CRIP1P1*	12715290	6.08×10^{-6}	0.515	20	eQTLGen
ENSG00000181789	3	*COPG1*	128982531	3.13×10^{-4}	0.500	20	eQTLGen
ENSG00000168811	3	*IL12A*	159710171	3.89×10^{-4}	0.283	12	eQTLGen
ENSG00000073282	3	*TP63*	189482136	2.49×10^{-4}	0.549	20	eQTLGen
ENSG00000112977	5	*DAP*	10720363	1.12×10^{-5}	0.431	20	eQTLGen
ENSG00000081059	5	*TCF7*	133468979	1.57×10^{-4}	0.283	20	eQTLGen
ENSG00000154319	8	*FAM167A*	11305598	4.34×10^{-6}	0.397	20	eQTLGen

© 2024 Fan J et al. *JAMA Network Open.*

ENSG00000205189	8	*ZBTB10*	81418177	3.73×10^{-5}	0.454	20	eQTLGen
ENSG00000119403	9	*PHF19*	123628791	7.71×10^{-7}	0.093	20	eQTLGen
ENSG00000134825	11	*TMEM258*	61558354	1.59×10^{-4}	0.346	20	eQTLGen
ENSG00000149485	11	*FADS1*	61581944	6.16×10^{-6}	0.705	20	eQTLGen
ENSG00000134824	11	*FADS2*	61597639	9.83×10^{-8}	0.525	20	eQTLGen
ENSG00000160683	11	*CXCR5*	118761491	7.67×10^{-6}	0.078	20	eQTLGen
ENSG00000139613	12	*SMARCC2*	56570059	6.19×10^{-5}	0.601	20	eQTLGen
ENSG00000166908	12	*PIP4K2C*	57991077	2.29×10^{-4}	0.093	20	eQTLGen
ENSG00000135506	12	*OS9*	58101539	5.67×10^{-5}	0.146	20	eQTLGen
ENSG00000139266	12	*MARCHF9*	58151535	2.56×10^{-4}	0.173	20	eQTLGen
ENSG00000037897	12	*METTL1*	58164415	1.54×10^{-5}	0.111	20	eQTLGen
ENSG00000123427	12	*EEF1AKMT3*	58170799	3.69×10^{-6}	0.086	20	eQTLGen
ENSG00000123297	12	*TSFM*	58189113	1.25×10^{-5}	0.051	20	eQTLGen
ENSG00000132964	13	*CDK8*	26903825	3.20×10^{-4}	0.177	20	eQTLGen
ENSG00000178952	16	*TUFM*	28855730	1.46×10^{-4}	0.168	20	eQTLGen
ENSG00000169682	16	*SPNS1*	28990705	4.80×10^{-5}	0.710	20	eQTLGen
ENSG00000156858	16	*PRR14*	30664899	1.07×10^{-5}	0.659	20	eQTLGen
ENSG00000180626	17	*ZNF594*	5089004	2.09×10^{-4}	0.142	20	eQTLGen
ENSG00000141744	17	*PNMT*	37825481	3.31×10^{-4}	0.107	20	eQTLGen
ENSG00000161395	17	*PGAP3*	37840212	4.91×10^{-7}	0.077	20	eQTLGen
ENSG00000141736	17	*ERBB2*	37865423	1.06×10^{-5}	0.804	20	eQTLGen
ENSG00000220201	19	*ZGLP1*	10418017	5.10×10^{-5}	0.095	17	eQTLGen
ENSG00000105397	19	*TYK2*	10476280	1.31×10^{-9}	0.073	20	eQTLGen
ENSG00000079999	19	*KEAP1*	10605606	3.73×10^{-4}	0.225	4	eQTLGen
ENSG00000127528	19	*KLF2*	16437156	3.76×10^{-4}	0.065	20	eQTLGen
ENSG00000186010	19	*NDUFA13*	19635415	5.18×10^{-5}	0.197	20	eQTLGen
ENSG00000124205	20	*EDN3*	57888264	6.43×10^{-5}	0.077	20	eQTLGen
ENSG00000159110	21	*IFNAR2*	34620087	1.55×10^{-4}	0.286	17	eQTLGen
ENSG00000159128	21	*IFNGR2*	34813428	3.10×10^{-7}	0.417	20	eQTLGen
ENSG00000142188	21	*TMEM50B*	34828555	7.79×10^{-5}	0.058	20	eQTLGen
ENSG00000159131	21	*GART*	34896017	8.93×10^{-5}	0.669	20	eQTLGen
ENSG00000100321	22	*SYNGR1*	39763761	8.60×10^{-11}	0.131	20	eQTLGen
ENSG00000100324	22	*TAB1*	39814405	2.67×10^{-4}	0.212	20	eQTLGen

ENSG00000204084	1	*INPP5B*	38326369	3.03×10^{-4}	0.188	20	GTEx
ENSG00000160856	1	*FCRL3*	157644111	7.01×10^{-6}	0.174	20	GTEx
ENSG00000163534	1	*FCRL1*	157764193	3.92×10^{-5}	0.086	20	GTEx
ENSG00000236278	1	*PEBP1P3*	198648268	7.90×10^{-4}	0.232	17	GTEx
ENSG00000152284	2	*TCF7L1*	85360533	4.72×10^{-4}	0.738	20	GTEx
ENSG00000246575	2	*AC093162.5*	85542164	3.76×10^{-5}	0.954	20	GTEx
ENSG00000042445	2	*RETSAT*	85569211	2.18×10^{-4}	0.453	20	GTEx
ENSG00000144218	2	*AFF3*	100162323	3.10×10^{-6}	0.061	20	GTEx
ENSG00000125637	2	*PSD4*	113914902	5.82×10^{-5}	0.291	20	GTEx
ENSG00000115896	2	*PLCL1*	198669426	9.17×10^{-5}	0.065	20	GTEx
ENSG00000134352	5	*IL6ST*	55230923	2.62×10^{-7}	0.092	6	GTEx
ENSG00000181315	6	*ZNF322*	26636518	3.42×10^{-4}	0.068	20	GTEx
ENSG00000112486	6	*CCR6*	167525295	3.79×10^{-5}	0.149	20	GTEx
ENSG00000005020	7	*SKAP2*	26706681	9.30×10^{-4}	0.102	20	GTEx
ENSG00000233429	7	*HOTAIRM1*	27135266	1.53×10^{-4}	0.116	20	GTEx
ENSG00000105997	7	*HOXA3*	27145803	1.00×10^{-3}	0.072	20	GTEx
ENSG00000154319	8	*FAM167A*	11278972	4.26×10^{-8}	0.112	20	GTEx
ENSG00000056558	9	*TRAF1*	123664671	4.66×10^{-6}	0.095	17	GTEx
ENSG00000148396	9	*SEC16A*	139334549	1.04×10^{-3}	0.942	20	GTEx
ENSG00000134825	11	*TMEM258*	61535973	1.59×10^{-4}	0.755	20	GTEx
ENSG00000134824	11	*FADS2*	61560452	1.72×10^{-7}	0.720	20	GTEx
ENSG00000149485	11	*FADS1*	61567099	1.00×10^{-4}	0.710	20	GTEx
ENSG00000236935	11	*AP003774.1*	64092522	7.59×10^{-5}	0.163	20	GTEx
ENSG00000139531	12	*SUOX*	56390964	2.28×10^{-6}	0.204	20	GTEx
ENSG00000123427	12	*METTL21B*	58165275	1.90×10^{-5}	0.158	20	GTEx
ENSG00000178952	16	*TUFM*	28853732	1.58×10^{-4}	0.153	20	GTEx
ENSG00000169682	16	*SPNS1*	28985542	2.93×10^{-5}	0.609	20	GTEx
ENSG00000103549	16	*RNF40*	30773066	2.38×10^{-5}	0.188	19	GTEx
ENSG00000161405	17	*IKZF3*	37921198	4.07×10^{-5}	0.295	20	GTEx
ENSG00000172057	17	*ORMDL3*	38077294	1.79×10^{-6}	0.090	20	GTEx
ENSG00000182566	19	*CLEC4G*	7793843	5.21×10^{-4}	0.681	20	GTEx
ENSG00000105397	19	*TYK2*	10461209	6.40×10^{-6}	0.101	20	GTEx
ENSG00000267100	19	*ILF3-AS1*	10762538	1.01×10^{-3}	0.072	20	GTEx

ENSG00000178093	19	*TSSK6*	19623227	2.51 x 10^{-4}	0.145	20	GTEx
ENSG00000124205	20	*EDN3*	57875482	3.93 x 10^{-4}	0.073	19	GTEx
ENSG00000125510	20	*OPRL1*	62711526	1.04 x 10^{-4}	0.763	20	GTEx
ENSG00000159128	21	*IFNGR2*	34775202	1.88 x 10^{-5}	0.169	20	GTEx
ENSG00000160223	21	*ICOSLG*	45642874	5.93 x 10^{-4}	0.293	11	GTEx
ENSG00000100321	22	*SYNGR1*	39745930	1.40 x 10^{-6}	0.136	20	GTEx

c.SMR SLE

probeID	ProbeChr	Gene	Probe_bp	p_SMR	p_HEIDI	nsnp_HEIDI	Database
ILMN_1657754	1	*RPAP2*	92853524	1.19 x 10^{-4}	0.732	20	CAGE
ILMN_1742026	1	*PYHIN1*	158914720	2.20 x 10^{-4}	0.486	20	CAGE
ILMN_2395981	1	*PYHIN1*	158914787	1.32 x 10^{-4}	0.561	20	CAGE
ILMN_1669447	1	*PYHIN1*	158946565	1.39 x 10^{-4}	0.542	20	CAGE
ILMN_1804010	2	*AK130848*	135040410	2.48 x 10^{-5}	0.136	20	CAGE
ILMN_1896967	5	*AK055485*	100241145	3.96 x 10^{-6}	0.462	20	CAGE
ILMN_1657129	7	*SKAP2*	26707336	1.38 x 10^{-4}	0.058	20	CAGE
ILMN_1753613	7	*HOXA5*	27181360	2.47 x 10^{-4}	0.200	20	CAGE
ILMN_1725338	8	*CLDN23*	8561488	1.84 x 10^{-4}	0.788	20	CAGE
ILMN_2349061	11	*IRF7*	613231	3.40 x 10^{-8}	0.394	20	CAGE
ILMN_2112915	11	*DRD4*	640560	2.27 x 10^{-6}	0.376	20	CAGE
ILMN_1689868	11	*TMEM80*	703833	2.98 x 10^{-7}	0.166	20	CAGE
ILMN_1669692	17	*IKZF3*	37921254	7.81 x 10^{-5}	0.184	19	CAGE
ILMN_2347193	17	*GSDMB*	38062136	2.28 x 10^{-5}	0.052	20	CAGE
ILMN_1748797	17	*GRB2*	73322031	2.70 x 10^{-5}	0.093	20	CAGE
ILMN_1687825	18	*CD226*	67530636	1.29 x 10^{-4}	0.675	20	CAGE
ILMN_1699908	19	*IL12RB1*	18183009	1.53 x 10^{-4}	0.453	20	CAGE
ILMN_1759436	19	*NOSIP*	50059807	1.77 x 10^{-4}	0.392	20	CAGE
ILMN_1765649	19	*IRF3*	50163024	1.47 x 10^{-4}	0.244	20	CAGE
ILMN_1677877	22	*UBE2L3*	21978213	8.89 x 10^{-13}	0.437	20	CAGE
ENSG00000223823	1	*LINC01342*	1075916	1.69 x 10^{-4}	0.247	20	eQTLGen
ENSG00000081985	1	*IL12RB2*	67817815	3.09 x 10^{-6}	0.074	20	eQTLGen
ENSG00000122484	1	*RPAP2*	92809126	1.92 x 10^{-4}	0.493	20	eQTLGen
ENSG00000131781	1	*FMO5*	146680815	2.07 x 10^{-4}	0.199	15	eQTLGen
ENSG00000030419	2	*IKZF2*	213940790	4.29 x 10^{-6}	0.260	20	eQTLGen
ENSG00000168297	3	*PXK*	58365177	6.30 x 10^{-6}	0.286	20	eQTLGen
ENSG00000168811	3	*IL12A*	159710171	1.14 x 10^{-4}	0.072	11	eQTLGen
ENSG00000164512	5	*ANKRD55*	55462346	1.88 x 10^{-4}	0.422	20	eQTLGen

© 2024 Fan J et al. *JAMA Network Open.*

ENSG00000065029	6	*ZNF76*	35245224	1.74×10^{-4}	0.051	20	eQTLGen
ENSG00000105991	7	*HOXA1*	27134113	1.91×10^{-4}	0.500	20	eQTLGen
ENSG00000233429	7	*HOTAIRM1*	27137575	1.31×10^{-4}	0.102	20	eQTLGen
ENSG00000105996	7	*HOXA2*	27141075	1.35×10^{-4}	0.070	20	eQTLGen
ENSG00000106004	7	*HOXA5*	27181979	1.49×10^{-4}	0.347	20	eQTLGen
ENSG00000253981	8	*ALG1L13P*	8097857	1.02×10^{-5}	0.461	20	eQTLGen
ENSG00000154319	8	*FAM167A*	11305598	7.52×10^{-18}	0.307	20	eQTLGen
ENSG00000136573	8	*BLK*	11386811	1.44×10^{-17}	0.052	20	eQTLGen
ENSG00000174915	11	*PTDSS2*	469830	1.67×10^{-5}	0.183	20	eQTLGen
ENSG00000254815	11	*LMNTD2-AS1*	558851	5.84×10^{-7}	0.084	20	eQTLGen
ENSG00000177042	11	*TMEM80*	700322	1.05×10^{-7}	0.071	20	eQTLGen
ENSG00000149289	11	*ZC3H12C*	110003326	9.53×10^{-5}	0.382	20	eQTLGen
ENSG00000223648	14	*IGHV3-64*	107114007	1.96×10^{-4}	0.163	20	eQTLGen
ENSG00000072818	17	*ACAP1*	7247322	3.33×10^{-6}	0.180	20	eQTLGen
ENSG00000213859	17	*KCTD11*	7256733	4.30×10^{-6}	0.118	20	eQTLGen
ENSG00000161405	17	*IKZF3*	37970819	1.18×10^{-4}	0.224	20	eQTLGen
ENSG00000177885	17	*GRB2*	73357973	1.87×10^{-5}	0.323	20	eQTLGen
ENSG00000206052	18	*DOK6*	67292307	8.47×10^{-5}	0.841	20	eQTLGen
ENSG00000150637	18	*CD226*	67563716	7.93×10^{-5}	0.747	20	eQTLGen
ENSG00000104886	19	*PLEKHJ1*	2233898	1.63×10^{-5}	0.170	20	eQTLGen
ENSG00000086544	19	*ITPKC*	41234886	5.57×10^{-5}	0.143	20	eQTLGen
ENSG00000126456	19	*IRF3*	50165979	9.12×10^{-5}	0.162	20	eQTLGen
ENSG00000105063	19	*PPP6R1*	55755755	2.81×10^{-7}	0.198	20	eQTLGen
ENSG00000185651	22	*UBE2L3*	21941029	1.85×10^{-13}	0.261	20	eQTLGen
ENSG00000081985	1	*IL12RB2*	67773047	9.56×10^{-6}	0.112	20	GTEx
ENSG00000163684	3	*RPP14*	58291974	7.95×10^{-5}	0.342	20	GTEx
ENSG00000168297	3	*PXK*	58318607	8.27×10^{-5}	0.303	20	GTEx
ENSG00000065029	6	*ZNF76*	35226686	4.06×10^{-4}	0.144	20	GTEx
ENSG00000023892	6	*DEF6*	35265595	5.45×10^{-5}	0.306	20	GTEx
ENSG00000005020	7	*SKAP2*	26706681	1.58×10^{-4}	0.144	20	GTEx
ENSG00000105991	7	*HOXA1*	27132612	3.67×10^{-4}	0.353	20	GTEx
ENSG00000233429	7	*HOTAIRM1*	27135266	1.60×10^{-4}	0.284	20	GTEx
ENSG00000105996	7	*HOXA2*	27139721	3.14×10^{-4}	0.522	20	GTEx

© 2024 Fan J et al. *JAMA Network Open.*

ENSG00000253552	7	*HOXA-AS2*	27147396	2.81×10^{-4}	0.430	20	GTEx
ENSG00000253981	8	*ALG1L13P*	8093525	3.32×10^{-4}	0.438	20	GTEx
ENSG00000269918	8	*AF131215.9*	10962201	1.83×10^{-5}	0.091	20	GTEx
ENSG00000255310	8	*AF131215.2*	10965298	2.92×10^{-5}	0.140	20	GTEx
ENSG00000154319	8	*FAM167A*	11278972	4.72×10^{-13}	0.066	20	GTEx
ENSG00000196072	10	*BLOC1S2*	102033713	2.51×10^{-4}	0.724	20	GTEx
ENSG00000149089	11	*APIP*	34874641	5.71×10^{-4}	0.488	20	GTEx
ENSG00000254682	11	*RP11-660L16.2*	71159720	3.33×10^{-4}	0.285	20	GTEx
ENSG00000172890	11	*NADSYN1*	71164155	2.44×10^{-4}	0.050	20	GTEx
ENSG00000149289	11	*ZC3H12C*	109964087	4.95×10^{-4}	0.260	19	GTEx
ENSG00000139899	14	*CBLN3*	24895738	2.82×10^{-4}	0.980	20	GTEx
ENSG00000197119	14	*SLC25A29*	100757448	4.07×10^{-4}	0.389	20	GTEx
ENSG00000211974	14	*IGHV2-70*	107178820	8.52×10^{-4}	0.222	20	GTEx
ENSG00000206190	15	*ATP10A*	25922420	8.23×10^{-4}	0.085	20	GTEx
ENSG00000072818	17	*ACAP1*	7239848	8.84×10^{-6}	0.333	20	GTEx
ENSG00000161405	17	*IKZF3*	37921198	6.77×10^{-4}	0.148	20	GTEx
ENSG00000262500	17	*RP11-259G18.2*	44320972	6.37×10^{-4}	0.507	3	GTEx
ENSG00000262539	17	*RP11-259G18.3*	44336917	3.30×10^{-4}	0.221	4	GTEx
ENSG00000125457	17	*MIF4GD*	73262309	1.89×10^{-4}	0.080	20	GTEx
ENSG00000177885	17	*GRB2*	73314157	4.58×10^{-4}	0.142	20	GTEx
ENSG00000206052	18	*DOK6*	67068291	5.19×10^{-4}	0.924	20	GTEx
ENSG00000105376	19	*ICAM5*	10400657	5.67×10^{-5}	0.057	13	GTEx
ENSG00000105397	19	*TYK2*	10461209	7.48×10^{-7}	0.467	20	GTEx
ENSG00000096996	19	*IL12RB1*	18169805	3.22×10^{-4}	0.766	20	GTEx
ENSG00000175489	19	*LRRC25*	18501954	7.06×10^{-4}	0.270	20	GTEx
ENSG00000268535	19	*RP11-420K14.3*	21892324	4.42×10^{-4}	0.056	20	GTEx
ENSG00000197020	19	*ZNF100*	21905568	5.29×10^{-4}	0.269	20	GTEx
ENSG00000241837	21	*ATP5O*	35275757	7.31×10^{-4}	0.905	20	GTEx
ENSG00000185651	22	*UBE2L3*	21903736	3.15×10^{-9}	0.112	15	GTEx

d.SMR SSc

probeID	ProbeChr	Gene	Probe_bp	p_SMR	p_HEIDI	nsnp_HEIDI	Database
ILMN_1703041	4	*IDUA*	997408	4.08×10^{-9}	0.139	20	CAGE
ILMN_1687213	8	*FAM167A*	11279135	3.48×10^{-18}	0.838	20	CAGE

© 2024 Fan J et al. *JAMA Network Open.*

ILMN_1668277	8	*BLK*	11421917	9.56×10^{-12}	0.385	20	CAGE
ILMN_1741404	8	*MSC*	72754186	4.18×10^{-5}	0.498	18	CAGE
ILMN_2112915	11	*DRD4*	640560	1.10×10^{-4}	0.055	20	CAGE
ILMN_1666206	17	*GSDMB*	38060997	4.14×10^{-6}	0.092	20	CAGE
ILMN_2347193	17	*GSDMB*	38062136	4.64×10^{-6}	0.204	20	CAGE
ILMN_2260756	17	*GSDMB*	38073727	4.08×10^{-5}	0.297	20	CAGE
ILMN_1662174	17	*ORMDL3*	38077572	4.49×10^{-6}	0.058	20	CAGE
ILMN_1815890	19	*IL12RB1*	18181449	5.81×10^{-7}	0.708	20	CAGE
ILMN_1810875	22	*SYNGR1*	39772125	6.51×10^{-5}	0.464	19	CAGE
ILMN_1727805	22	*SYNGR1*	39781479	2.11×10^{-5}	0.841	19	CAGE
ENSG00000168811	3	*IL12A*	159710171	5.21×10^{-6}	0.160	9	eQTLGen
ENSG00000154319	8	*FAM167A*	11305598	5.92×10^{-21}	0.783	20	eQTLGen
ENSG00000136573	8	*BLK*	11386811	1.43×10^{-20}	0.346	20	eQTLGen
ENSG00000178860	8	*MSC*	72755243	2.08×10^{-5}	0.262	20	eQTLGen
ENSG00000235531	8	*MSC-AS1*	72885515	4.41×10^{-5}	0.224	18	eQTLGen
ENSG00000103653	15	*CSK*	75084968	2.18×10^{-8}	0.585	20	eQTLGen
ENSG00000073605	17	*GSDMB*	38068477	3.44×10^{-6}	0.090	20	eQTLGen
ENSG00000172057	17	*ORMDL3*	38080574	3.47×10^{-6}	0.079	20	eQTLGen
ENSG00000099308	19	*MAST3*	18235548	6.07×10^{-5}	0.458	20	eQTLGen
ENSG00000185651	22	*UBE2L3*	21941029	8.87×10^{-5}	0.285	12	eQTLGen
ENSG00000100321	22	*SYNGR1*	39763761	1.47×10^{-5}	0.566	20	eQTLGen
ENSG00000168291	3	*PDHB*	58413357	6.45×10^{-7}	0.154	20	GTEx
ENSG00000127415	4	*IDUA*	980785	2.66×10^{-7}	0.073	20	GTEx
ENSG00000154319	8	*FAM167A*	11278972	5.52×10^{-11}	0.777	20	GTEx
ENSG00000136573	8	*BLK*	11351510	2.18×10^{-9}	0.181	20	GTEx
ENSG00000255354	8	*RP11-148O21.2*	11415975	7.24×10^{-8}	0.146	15	GTEx
ENSG00000073605	17	*GSDMB*	38060848	5.70×10^{-6}	0.319	20	GTEx
ENSG00000172057	17	*ORMDL3*	38077294	7.53×10^{-6}	0.135	20	GTEx
ENSG00000100321	22	*SYNGR1*	39745930	4.60×10^{-5}	0.292	19	GTEx

© 2024 Fan J et al. *JAMA Network Open.*

eTable 10. Candidate genes identified by TWAS with FDR <0.05.

a. TWAS JIA

gene	gene_name	zscore	pvalue	var_g	n_snps_used	n_snps_in_cov	n_snps_in_model	best_gwas_p	largest_weight
ENSG00000164512.17	*ANKRD55*	9.52	1.77×10^{-21}	0.010	2	2	2	7.03×10^{-21}	0.137
ENSG00000134352.19	*IL6ST*	-8.23	1.85×10^{-16}	0.005	2	2	2	1.36×10^{-15}	0.138
ENSG00000105397.13	*TYK2*	-6.73	1.65×10^{-11}	0.004	2	3	3	2.96×10^{-11}	0.103
ENSG00000164308.16	*ERAP2*	5.44	5.40×10^{-8}	0.552	2	2	2	6.69×10^{-8}	1.004
ENSG00000146109.4	*ABT1*	-5.08	3.74×10^{-7}	0.034	3	3	3	1.18×10^{-4}	0.495
ENSG00000184730.10	*APOBR*	-4.97	6.80×10^{-7}	0.001	2	2	2	1.92×10^{-6}	0.050
ENSG00000161405.16	*IKZF3*	4.92	8.61×10^{-7}	0.003	1	1	1	8.61×10^{-7}	0.074
ENSG00000233232.6	*NPIPB7*	-4.69	2.78×10^{-6}	0.090	1	1	1	2.78×10^{-6}	0.429
ENSG00000197165.10	*SULT1A2*	4.65	3.29×10^{-6}	1.708	3	4	4	3.55×10^{-6}	0.943
ENSG00000198821.10	*CD247*	-4.59	4.35×10^{-6}	0.001	3	3	3	8.27×10^{-7}	0.036
ENSG00000073605.18	*GSDMB*	-4.49	7.25×10^{-6}	0.098	3	3	3	6.37×10^{-7}	0.339
ENSG00000113441.15	*LNPEP*	4.47	7.66×10^{-6}	0.012	2	2	2	3.91×10^{-7}	0.154
ENSG00000188603.18	*CLN3*	-4.44	8.89×10^{-6}	0.026	3	3	3	1.82×10^{-8}	0.154
ENSG00000188603.18	*CLN3*	-4.44	8.89×10^{-6}	0.026	3	3	3	1.82×10^{-8}	0.154
ENSG00000196502.11	*SULT1A1*	-4.30	1.74×10^{-5}	0.167	5	7	7	2.17×10^{-7}	0.595
ENSG00000118655.4	*DCLRE1B*	-4.26	2.09×10^{-5}	0.003	1	1	1	2.09×10^{-5}	0.099
ENSG00000185651.14	*UBE2L3*	4.18	2.88×10^{-5}	0.007	1	1	1	2.88×10^{-5}	0.153
ENSG00000185347.17	*C14orf80*	4.18	2.91×10^{-5}	0.002	2	2	2	2.87×10^{-5}	0.080
ENSG00000168488.18	*ATXN2L*	-4.16	3.12×10^{-5}	0.004	1	1	1	3.12×10^{-5}	0.091
ENSG00000196296.13	*ATP2A1*	4.15	3.35×10^{-5}	0.007	1	1	1	3.35×10^{-5}	0.121
ENSG00000107796.13	*ACTA2*	-4.10	4.10×10^{-5}	0.097	1	1	1	4.10×10^{-5}	0.436
ENSG00000114988.11	*LMAN2L*	4.05	5.13×10^{-5}	0.013	2	2	2	8.44×10^{-5}	0.141
ENSG00000115652.14	*UXS1*	4.03	5.64×10^{-5}	0.006	2	2	2	1.18×10^{-4}	0.139
ENSG00000130518.16	*KIAA1683*	-4.03	5.68×10^{-5}	0.098	3	3	3	1.39×10^{-5}	0.245
ENSG00000161180.10	*CCDC116*	-4.00	6.25×10^{-5}	0.031	1	1	1	6.25×10^{-5}	0.329
ENSG00000258366.7	*RTEL1*	3.99	6.73×10^{-5}	0.004	2	2	2	1.42×10^{-4}	0.100
ENSG00000096996.15	*IL12RB1*	3.96	7.50×10^{-5}	0.034	2	2	2	6.78×10^{-5}	0.304
ENSG00000137338.5	*PGBD1*	-3.95	7.68×10^{-5}	0.018	2	2	2	1.16×10^{-3}	0.208
ENSG00000113810.15	*SMC4*	-3.93	8.59×10^{-5}	0.004	2	2	2	8.79×10^{-5}	0.093
ENSG00000181315.10	*ZNF322*	4.32	1.56×10^{-5}	0.116	2	2	2	5.72×10^{-5}	0.302

© 2024 Fan J et al. *JAMA Network Open.*

ENSG00000164047.4	*CAMP*	3.87	1.08×10^{-4}	0.000	1	1	1	1.08×10^{-4}	0.062
ENSG00000185338.4	*SOCS1*	-3.84	1.21×10^{-4}	0.003	2	2	2	1.09×10^{-4}	0.081
ENSG00000260302.1	*RP11-973H7.1*	3.79	1.50×10^{-4}	0.148	4	4	4	1.93×10^{-4}	0.352
ENSG00000172057.9	*ORMDL3*	-3.78	1.57×10^{-4}	0.137	2	2	2	5.07×10^{-5}	0.398
ENSG00000110321.16	*EIF4G2*	-3.78	1.58×10^{-4}	0.001	1	1	1	1.58×10^{-4}	0.048
ENSG00000186207.4	*LCE5A*	3.73	1.95×10^{-4}	0.013	2	2	2	8.35×10^{-4}	0.122
ENSG00000184986.10	*TMEM121*	3.71	2.10×10^{-4}	0.449	2	2	2	2.15×10^{-4}	0.553
ENSG00000135541.20	*AHI1*	-3.68	2.34×10^{-4}	0.132	2	2	2	7.30×10^{-6}	0.446
ENSG00000165916.8	*PSMC3*	-3.66	2.56×10^{-4}	0.003	2	2	2	1.05×10^{-3}	0.057
ENSG00000141741.11	*MIEN1*	3.64	2.77×10^{-4}	0.000	1	1	1	2.77×10^{-4}	0.010
ENSG00000144218.18	*AFF3*	3.63	2.83×10^{-4}	0.009	2	2	2	5.29×10^{-5}	0.096
ENSG00000128604.19	*IRF5*	3.61	3.09×10^{-4}	0.089	2	2	2	2.88×10^{-5}	0.387
ENSG00000213398.7	*LCAT*	3.60	3.17×10^{-4}	0.017	1	1	1	3.17×10^{-4}	0.260
ENSG00000213658.11	*LAT*	3.55	3.85×10^{-4}	0.001	2	2	2	4.57×10^{-6}	0.144
ENSG00000143093.14	*STRIP1*	3.55	3.86×10^{-4}	0.009	2	2	2	2.55×10^{-4}	0.136
ENSG00000197857.13	*ZNF44*	3.55	3.87×10^{-4}	0.007	1	1	1	3.87×10^{-4}	0.158
ENSG00000254858.9	*MPV17L2*	-3.54	3.94×10^{-4}	0.042	2	3	3	4.10×10^{-4}	0.168
ENSG00000168071.21	*CCDC88B*	3.54	4.01×10^{-4}	0.003	1	1	1	4.01×10^{-4}	0.083
ENSG00000161395.13	*PGAP3*	-3.53	4.18×10^{-4}	0.002	1	2	2	4.18×10^{-4}	0.069
ENSG00000149177.12	*PTPRJ*	3.50	4.65×10^{-4}	0.018	2	2	2	1.55×10^{-4}	0.118

b. TWAS RA

gene	gene_name	zscore	pvalue	var_g	n_snps_used	n_snps_in_cov	n_snps_in_model	best_gwas_p	largest_weight
ENSG00000118655.4	*DCLRE1B*	-15.77	4.72×10^{-56}	0.003	1	1	1	4.72×10^{-56}	0.099
ENSG00000164512.17	*ANKRD55*	9.11	8.01×10^{-20}	0.010	2	2	2	5.05×10^{-20}	0.137
ENSG00000110367.11	*DDX6*	8.47	2.44×10^{-17}	0.004	1	2	2	2.44×10^{-17}	0.099
ENSG00000134352.19	*IL6ST*	-8.13	4.16×10^{-16}	0.005	2	2	2	1.61×10^{-16}	0.138
ENSG00000105397.13	*TYK2*	-7.08	1.43×10^{-12}	0.004	2	3	3	1.26×10^{-11}	0.103
ENSG00000144218.18	*AFF3*	6.85	7.30×10^{-12}	0.009	2	2	2	1.68×10^{-11}	0.096
ENSG00000137338.5	*PGBD1*	-6.83	8.45×10^{-12}	0.018	2	2	2	5.91×10^{-10}	0.208
ENSG00000128604.19	*IRF5*	6.03	1.60×10^{-9}	0.089	2	2	2	7.31×10^{-11}	0.387
ENSG00000081026.18	*MAGI3*	-5.85	4.87×10^{-9}	0.001	1	1	1	4.87×10^{-9}	0.075

© 2024 Fan J et al. *JAMA Network Open.*

ENSG00000160185.14	*UBASH3A*	-5.67	1.40×10^{-8}	0.027	1	1	1	1.40×10^{-8}	0.250
ENSG00000161405.16	*IKZF3*	5.64	1.74×10^{-8}	0.003	1	1	1	1.74×10^{-8}	0.074
ENSG00000139531.12	*SUOX*	5.61	2.07×10^{-8}	0.036	3	3	3	1.66×10^{-7}	0.246
ENSG00000112486.15	*CCR6*	5.55	2.83×10^{-8}	0.016	2	2	2	2.08×10^{-8}	0.164
ENSG00000100321.14	*SYNGR1*	5.48	4.19×10^{-8}	0.061	2	2	2	2.82×10^{-7}	0.427
ENSG00000260302.1	*RP11-973H7.1*	5.37	7.72×10^{-8}	0.148	4	4	4	5.67×10^{-8}	0.352
ENSG00000056558.10	*TRAF1*	5.35	8.89×10^{-8}	0.022	2	3	3	8.49×10^{-10}	0.205
ENSG00000100316.15	*RPL3*	-5.32	1.05×10^{-7}	0.000	1	2	2	1.05×10^{-7}	0.026
ENSG00000065361.14	*ERBB3*	5.26	1.43×10^{-7}	0.000	1	1	1	1.43×10^{-7}	0.032
ENSG00000161395.13	*PGAP3*	-5.23	1.66×10^{-7}	0.002	1	2	2	1.66×10^{-7}	0.069
ENSG00000160856.20	*FCRL3*	5.12	3.09×10^{-7}	0.091	2	2	2	1.05×10^{-7}	0.304
ENSG00000204084.12	*INPP5B*	5.09	3.66×10^{-7}	0.645	4	4	4	4.24×10^{-7}	0.505
ENSG00000159128.14	*IFNGR2*	-5.08	3.80×10^{-7}	0.004	1	1	1	3.80×10^{-7}	0.139
ENSG00000134824.13	*FADS2*	-5.08	3.87×10^{-7}	1.066	2	2	2	9.45×10^{-8}	1.302
ENSG00000141741.11	*MIEN1*	5.07	4.00×10^{-7}	0.000	1	1	1	4.00×10^{-7}	0.010
ENSG00000181315.10	*ZNF322*	3.40	6.79×10^{-4}	0.116	2	2	2	4.20×10^{-4}	0.302
ENSG00000197982.13	*C1orf122*	-5.06	4.25×10^{-7}	0.000	2	2	2	2.64×10^{-5}	0.013
ENSG00000189134.3	*NKAPL*	-5.03	4.94×10^{-7}	0.006	2	2	2	5.18×10^{-14}	0.184
ENSG00000163508.12	*EOMES*	5.01	5.44×10^{-7}	0.000	1	1	1	5.44×10^{-7}	0.020
ENSG00000101017.13	*CD40*	4.94	7.80×10^{-7}	0.143	3	3	3	9.09×10^{-12}	0.412
ENSG00000137077.7	*CCL21*	4.94	7.83×10^{-7}	0.000	1	1	1	7.83×10^{-7}	0.005
ENSG00000235109.7	*ZSCAN31*	4.86	1.16×10^{-6}	0.001	1	1	1	1.16×10^{-6}	0.110
ENSG00000164691.16	*TAGAP*	4.85	1.22×10^{-6}	0.002	2	2	2	5.66×10^{-5}	0.097
ENSG00000157870.14	*FAM213B*	4.83	1.36×10^{-6}	0.046	2	3	3	5.14×10^{-9}	0.417
ENSG00000163534.14	*FCRL1*	-4.80	1.63×10^{-6}	0.098	2	2	2	1.58×10^{-5}	0.337
ENSG00000277157.1	*HIST1H4D*	-4.79	1.71×10^{-6}	0.000	1	1	1	1.71×10^{-6}	0.012
ENSG00000073605.18	*GSDMB*	-4.74	2.13×10^{-6}	0.098	3	3	3	5.44×10^{-7}	0.339
ENSG00000136573.12	*BLK*	-4.73	2.20×10^{-6}	0.106	3	3	3	1.27×10^{-7}	0.243
ENSG00000237232.7	*ZNF295-AS1*	4.69	2.76×10^{-6}	0.012	3	3	3	3.92×10^{-4}	0.109
ENSG00000102870.5	*ZNF629*	-4.67	2.95×10^{-6}	0.000	1	1	1	2.95×10^{-6}	0.015
ENSG00000123297.17	*TSFM*	4.63	3.63×10^{-6}	0.004	1	1	1	3.63×10^{-6}	0.097
ENSG00000197728.9	*RPS26*	-4.63	3.63×10^{-6}	0.564	1	1	1	3.63×10^{-6}	1.094
ENSG00000123427.16	*METTL21B*	-4.63	3.63×10^{-6}	0.195	1	1	1	3.63×10^{-6}	0.701

© 2024 Fan J et al. *JAMA Network Open.*

ENSG00000037897.16	*METTL1*	-4.63	3.63 x 10^{-6}	0.000	1	1	1	3.63 x 10^{-6}	0.000
ENSG00000152284.4	*TCF7L1*	-4.57	4.81 x 10^{-6}	0.044	2	2	2	4.58 x 10^{-6}	0.309
ENSG00000172057.9	*ORMDL3*	-4.52	6.05 x 10^{-6}	0.137	2	2	2	2.26 x 10^{-6}	0.398
ENSG00000103549.21	*RNF40*	4.46	8.21 x 10^{-6}	0.009	2	2	2	8.78 x 10^{-6}	0.160
ENSG00000135506.15	*OS9*	4.41	1.04 x 10^{-5}	0.001	1	1	1	1.04 x 10^{-5}	0.059
ENSG00000142606.15	*MMEL1*	4.41	1.05 x 10^{-5}	0.327	2	2	2	2.08 x 10^{-8}	0.522
ENSG00000141736.13	*ERBB2*	-4.38	1.19 x 10^{-5}	0.000	2	2	2	2.95 x 10^{-6}	0.019
ENSG00000134825.15	*TMEM258*	-4.37	1.26 x 10^{-5}	0.010	2	2	2	1.45 x 10^{-4}	0.268
ENSG00000081985.10	*IL12RB2*	4.35	1.39 x 10^{-5}	0.037	2	2	2	1.40 x 10^{-5}	0.351
ENSG00000161180.10	*CCDC116*	-4.32	1.58 x 10^{-5}	0.031	1	1	1	1.58 x 10^{-5}	0.329
ENSG00000256812.1	*CAPNS2*	-4.32	1.58 x 10^{-5}	0.000	1	1	1	1.58 x 10^{-5}	0.002
ENSG00000167491.17	*GATAD2A*	4.32	1.58 x 10^{-5}	0.014	1	1	1	1.58 x 10^{-5}	0.177
ENSG00000162927.13	*PUS10*	4.32	1.58 x 10^{-5}	0.013	1	1	1	1.58 x 10^{-5}	0.213
ENSG00000119403.13	*PHF19*	-4.15	3.32 x 10^{-5}	0.004	2	2	2	1.45 x 10^{-4}	0.082
ENSG00000169682.17	*SPNS1*	4.15	3.34 x 10^{-5}	0.049	2	2	2	5.23 x 10^{-5}	0.330
ENSG00000166323.12	*C11orf65*	-4.14	3.44 x 10^{-5}	0.002	1	1	1	3.44 x 10^{-5}	0.087
ENSG00000125510.15	*OPRL1*	-4.11	4.01 x 10^{-5}	0.019	1	1	1	4.01 x 10^{-5}	0.198
ENSG00000177548.12	*RABEP2*	4.07	4.68 x 10^{-5}	0.001	1	1	1	4.68 x 10^{-5}	0.041
ENSG00000185651.14	*UBE2L3*	4.07	4.68 x 10^{-5}	0.007	1	1	1	4.68 x 10^{-5}	0.153
ENSG00000162928.8	*PEX13*	4.07	4.68 x 10^{-5}	0.000	1	1	1	4.68 x 10^{-5}	0.026
ENSG00000125637.15	*PSD4*	-4.07	4.68 x 10^{-5}	0.084	1	1	1	4.68 x 10^{-5}	0.416
ENSG00000141744.3	*PNMT*	-4.03	5.49 x 10^{-5}	0.014	1	1	1	5.49 x 10^{-5}	0.170
ENSG00000064419.13	*TNPO3*	-4.03	5.53 x 10^{-5}	0.046	2	2	2	3.92 x 10^{-4}	0.339
ENSG00000142748.12	*FCN3*	4.02	5.75 x 10^{-5}	0.000	1	1	1	5.75 x 10^{-5}	0.010
ENSG00000145725.19	*PPIP5K2*	3.98	6.84 x 10^{-5}	0.124	2	2	2	1.04 x 10^{-5}	0.279
ENSG00000213066.11	*FGFR1OP*	3.96	7.58 x 10^{-5}	0.016	2	2	2	5.48 x 10^{-5}	0.178
ENSG00000277224.2	*HIST1H2BF*	3.88	1.04 x 10^{-4}	0.011	3	3	3	6.63 x 10^{-6}	0.202
ENSG00000254206.5	*NPIPB11*	3.80	1.45 x 10^{-4}	0.018	1	1	1	1.45 x 10^{-4}	0.190
ENSG00000156853.12	*ZNF689*	3.80	1.45 x 10^{-4}	0.000	1	1	1	1.45 x 10^{-4}	0.004
ENSG00000168071.21	*CCDC88B*	3.80	1.45 x 10^{-4}	0.003	1	1	1	1.45 x 10^{-4}	0.083
ENSG00000175606.10	*TMEM70*	3.80	1.45 x 10^{-4}	0.001	1	1	1	1.45 x 10^{-4}	0.035
ENSG00000142188.16	*TMEM50B*	3.80	1.45 x 10^{-4}	0.047	1	1	1	1.45 x 10^{-4}	0.315
ENSG00000115896.15	*PLCL1*	-3.80	1.46 x 10^{-4}	0.069	1	1	1	1.46 x 10^{-4}	0.394

© 2024 Fan J et al. *JAMA Network Open.*

ENSG00000132155.11	*RAF1*	3.80	1.46×10^{-4}	0.001	1	1	1	1.46×10^{-4}	0.048
ENSG00000182004.12	*SNRPE*	-3.79	1.53×10^{-4}	0.005	2	2	2	2.06×10^{-4}	0.147
ENSG00000160161.9	*CILP2*	-3.78	1.57×10^{-4}	0.001	1	1	1	1.57×10^{-4}	0.064
ENSG00000159363.17	*ATP13A2*	3.70	2.12×10^{-4}	0.014	2	2	2	2.04×10^{-4}	0.104
ENSG00000104853.15	*CLPTM1*	-3.70	2.18×10^{-4}	0.001	2	2	2	1.42×10^{-3}	0.168
ENSG00000178093.13	*TSSK6*	3.68	2.34×10^{-4}	0.013	1	1	1	2.34×10^{-4}	0.210
ENSG00000169291.9	*SHE*	-3.63	2.78×10^{-4}	0.001	1	1	1	2.78×10^{-4}	0.068
ENSG00000198369.9	*SPRED2*	-3.63	2.80×10^{-4}	0.003	2	2	2	1.50×10^{-5}	0.070
ENSG00000102882.11	*MAPK3*	-3.58	3.42×10^{-4}	0.063	2	2	2	3.63×10^{-6}	0.189
ENSG00000196296.13	*ATP2A1*	3.58	3.50×10^{-4}	0.007	1	1	1	3.50×10^{-4}	0.121
ENSG00000121406.8	*ZNF549*	-3.55	3.92×10^{-4}	0.001	1	1	1	3.92×10^{-4}	0.037
ENSG00000088298.12	*EDEM2*	3.54	3.96×10^{-4}	0.021	2	2	2	3.92×10^{-4}	0.150
ENSG00000135744.7	*AGT*	-3.51	4.46×10^{-4}	0.012	2	2	2	3.68×10^{-5}	0.331
ENSG00000170515.13	*PA2G4*	-3.51	4.55×10^{-4}	0.000	2	2	2	2.64×10^{-5}	0.013
ENSG00000183621.15	*ZNF438*	-3.49	4.78×10^{-4}	0.009	3	3	3	1.20×10^{-3}	0.090
ENSG00000182952.4	*HMGN4*	-3.49	4.81×10^{-4}	0.139	3	3	3	9.22×10^{-5}	0.523
ENSG00000161847.13	*RAVER1*	-3.49	4.91×10^{-4}	0.003	2	2	2	1.47×10^{-6}	0.108
ENSG00000111087.9	*GLI1*	3.48	4.93×10^{-4}	0.001	2	2	2	3.42×10^{-3}	0.093
ENSG00000215912.12	*TTC34*	-3.48	5.06×10^{-4}	0.028	2	2	2	9.50×10^{-4}	0.250
ENSG00000145723.16	*GIN1*	3.47	5.13×10^{-4}	0.002	1	1	1	5.13×10^{-4}	0.071
ENSG00000010704.18	*HFE*	3.44	5.79×10^{-4}	0.002	1	1	1	5.79×10^{-4}	0.067
ENSG00000168488.18	*ATXN2L*	-3.44	5.79×10^{-4}	0.004	1	1	1	5.79×10^{-4}	0.091
ENSG00000108219.14	*TSPAN14*	3.44	5.79×10^{-4}	0.004	1	1	1	5.79×10^{-4}	0.089
ENSG00000145730.20	*PAM*	-3.44	5.79×10^{-4}	0.191	1	1	1	5.79×10^{-4}	0.666
ENSG00000167419.10	*LPO*	3.44	5.80×10^{-4}	0.000	2	2	2	9.50×10^{-4}	0.031
ENSG00000137700.17	*SLC37A4*	-3.43	6.11×10^{-4}	0.003	1	1	1	6.11×10^{-4}	0.102
ENSG00000170128.4	*GPR25*	3.42	6.30×10^{-4}	0.060	2	2	2	5.13×10^{-4}	0.225
ENSG00000153094.22	*BCL2L11*	3.39	6.89×10^{-4}	0.002	1	2	2	6.89×10^{-4}	0.097
ENSG00000184897.5	*H1FX*	-3.38	7.30×10^{-4}	0.002	2	2	2	4.68×10^{-5}	0.065
ENSG00000166908.17	*PIP4K2C*	3.38	7.34×10^{-4}	0.006	2	2	2	1.45×10^{-4}	0.103
ENSG00000176293.19	*ZNF135*	3.36	7.67×10^{-4}	0.018	2	2	2	1.46×10^{-3}	0.207
ENSG00000123411.14	*IKZF4*	-3.36	7.77×10^{-4}	0.005	1	1	1	7.77×10^{-4}	0.193
ENSG00000105991.7	*HOXA1*	-3.35	8.20×10^{-4}	0.067	1	1	1	8.20×10^{-4}	0.359

© 2024 Fan J et al. *JAMA Network Open.*

ENSG00000277075.2	*HIST1H2AE*	-3.34	8.33×10^{-4}	0.011	2	2	2	4.40×10^{-3}	0.206

c. TWAS SLE

gene	gene_name	zscore	pvalue	var_g	n_snps_used	n_snps_in_cov	n_snps_in_model	best_gwas_p	largest_weight
ENSG00000128604.19	*IRF5*	12.45	1.42×10^{-35}	0.089	2	2	2	1.59×10^{-36}	0.387
ENSG00000187626.8	*ZKSCAN4*	12.29	1.05×10^{-34}	0.006	1	1	1	1.05×10^{-34}	0.189
ENSG00000158691.14	*ZSCAN12*	-11.32	1.09×10^{-29}	0.000	2	2	2	3.19×10^{-34}	0.035
ENSG00000186470.13	*BTN3A2*	-9.90	4.15×10^{-23}	0.962	3	3	3	4.16×10^{-23}	1.054
ENSG00000064419.13	*TNPO3*	-9.28	1.63×10^{-20}	0.046	2	2	2	1.46×10^{-17}	0.339
ENSG00000197279.3	*ZNF165*	8.40	4.46×10^{-17}	0.013	2	2	2	2.08×10^{-16}	0.175
ENSG00000136573.12	*BLK*	-7.31	2.65×10^{-13}	0.106	3	3	3	7.23×10^{-15}	0.243
ENSG00000105397.13	*TYK2*	-7.31	2.72×10^{-13}	0.004	2	3	3	9.60×10^{-13}	0.103
ENSG00000261353.1	*CTA-14H9.5*	-7.16	8.05×10^{-13}	0.010	2	2	2	1.63×10^{-22}	0.498
ENSG00000185651.14	*UBE2L3*	6.98	2.97×10^{-12}	0.007	1	1	1	2.97×10^{-12}	0.153
ENSG00000161180.10	*CCDC116*	-6.86	7.04×10^{-12}	0.031	1	1	1	7.04×10^{-12}	0.329
ENSG00000189298.13	*ZKSCAN3*	6.80	1.07×10^{-11}	0.013	3	3	3	3.99×10^{-34}	0.541
ENSG00000140968.10	*IRF8*	-6.73	1.68×10^{-11}	0.004	2	2	2	1.80×10^{-11}	0.130
ENSG00000272462.2	*U91328.19*	6.28	3.32×10^{-10}	0.051	3	3	3	9.12×10^{-21}	0.427
ENSG00000272468.1	*RP1-86C11.7*	-6.07	1.30×10^{-9}	0.000	1	1	1	1.30×10^{-9}	0.026
ENSG00000069696.6	*DRD4*	5.83	5.53×10^{-9}	0.011	1	2	2	5.53×10^{-9}	0.168
ENSG00000233822.4	*HIST1H2BN*	-5.72	1.04×10^{-8}	0.011	2	2	2	1.08×10^{-32}	0.389
ENSG00000247095.2	*MIR210HG*	-5.52	3.44×10^{-8}	0.001	1	1	1	3.44×10^{-8}	0.052
ENSG00000168297.15	*PXK*	5.42	6.12×10^{-8}	0.010	3	3	3	1.29×10^{-7}	0.154
ENSG00000137338.5	*PGBD1*	5.19	2.16×10^{-7}	0.018	2	2	2	7.84×10^{-10}	0.208
ENSG00000124508.16	*BTN2A2*	5.14	2.78×10^{-7}	0.006	3	3	3	2.09×10^{-7}	0.116
ENSG00000198369.9	*SPRED2*	-5.13	2.91×10^{-7}	0.003	2	2	2	3.42×10^{-6}	0.070
ENSG00000104885.17	*DOT1L*	4.91	9.20×10^{-7}	0.012	4	4	4	2.29×10^{-5}	0.288
ENSG00000158373.8	*HIST1H2BD*	-4.84	1.29×10^{-6}	0.028	1	1	1	1.29×10^{-6}	0.251
ENSG00000168291.12	*PDHB*	-4.78	1.74×10^{-6}	0.020	2	2	2	3.14×10^{-7}	0.183
ENSG00000161847.13	*RAVER1*	-4.68	2.81×10^{-6}	0.003	2	2	2	5.10×10^{-9}	0.108
ENSG00000177042.14	*TMEM80*	-4.68	2.81×10^{-6}	0.188	3	3	3	1.69×10^{-7}	0.336
ENSG00000177030.16	*DEAF1*	-4.63	3.58×10^{-6}	0.016	1	1	1	3.58×10^{-6}	0.185

© 2024 Fan J et al. *JAMA Network Open.*

ENSG00000072818.11	*ACAP1*	-4.62	3.78×10^{-6}	0.025	2	2	2	2.95×10^{-6}	0.264
ENSG00000081985.10	*IL12RB2*	4.51	6.58×10^{-6}	0.037	2	2	2	6.68×10^{-6}	0.351
ENSG00000125454.11	*SLC25A19*	4.40	1.10×10^{-5}	0.008	1	1	1	1.10×10^{-5}	0.143
ENSG00000133265.10	*HSPBP1*	-4.39	1.13×10^{-5}	0.001	2	2	2	6.49×10^{-3}	0.081
ENSG00000163684.11	*RPP14*	-4.34	1.44×10^{-5}	0.029	2	2	2	2.49×10^{-6}	0.172
ENSG00000163684.11	*RPP14*	-4.34	1.44×10^{-5}	0.029	2	2	2	2.49×10^{-6}	0.172
ENSG00000184378.2	*ACTRT3*	4.31	1.60×10^{-5}	0.002	1	1	1	1.60×10^{-5}	0.083
ENSG00000118655.4	*DCLRE1B*	-4.28	1.87×10^{-5}	0.003	1	1	1	1.87×10^{-5}	0.099
ENSG00000161405.16	*IKZF3*	4.28	1.87×10^{-5}	0.003	1	1	1	1.87×10^{-5}	0.074
ENSG00000086544.2	*ITPKC*	-4.26	2.03×10^{-5}	0.002	2	2	2	1.36×10^{-5}	0.095
ENSG00000023892.10	*DEF6*	-4.26	2.03×10^{-5}	0.027	2	2	2	1.94×10^{-5}	0.272
ENSG00000103653.16	*CSK*	4.22	2.49×10^{-5}	0.001	1	1	1	2.49×10^{-5}	0.049
ENSG00000131591.17	*C1orf159*	-4.21	2.60×10^{-5}	0.007	2	2	2	1.46×10^{-3}	0.131
ENSG00000082641.15	*NFE2L1*	-4.19	2.77×10^{-5}	0.001	1	1	1	2.77×10^{-5}	0.083
ENSG00000065029.14	*ZNF76*	-4.18	2.90×10^{-5}	0.008	1	1	1	2.90×10^{-5}	0.150
ENSG00000149089.12	*APIP*	-4.10	4.17×10^{-5}	0.076	3	3	3	2.96×10^{-4}	0.476
ENSG00000173660.11	*UQCRH*	4.07	4.64×10^{-5}	0.000	1	1	1	4.64×10^{-5}	0.009
ENSG00000137185.11	*ZSCAN9*	-3.99	6.66×10^{-5}	0.095	3	3	3	6.60×10^{-33}	1.047
ENSG00000161328.10	*LRRC56*	3.99	6.68×10^{-5}	0.064	2	2	2	8.58×10^{-6}	0.413
ENSG00000149289.10	*ZC3H12C*	-3.99	6.72×10^{-5}	0.025	2	2	2	6.42×10^{-5}	0.255
ENSG00000132128.16	*LRRC41*	3.98	6.84×10^{-5}	0.003	2	2	2	4.64×10^{-5}	0.251
ENSG00000273542.1	*HIST1H4K*	-3.96	7.42×10^{-5}	0.011	2	2	2	3.97×10^{-23}	0.366
ENSG00000096968.13	*JAK2*	3.94	8.29×10^{-5}	0.000	1	1	1	8.29×10^{-5}	0.025
ENSG00000241837.6	*ATP5O*	-3.93	8.52×10^{-5}	0.004	1	1	1	8.52×10^{-5}	0.098
ENSG00000105245.9	*NUMBL*	3.92	9.03×10^{-5}	0.021	3	3	3	4.43×10^{-6}	0.160
ENSG00000005020.12	*SKAP2*	-3.91	9.24×10^{-5}	0.121	2	2	2	1.28×10^{-4}	0.335
ENSG00000128815.19	*WDFY4*	3.91	9.37×10^{-5}	0.035	2	2	2	1.34×10^{-3}	0.408
ENSG00000115419.12	*GLS*	3.90	9.69×10^{-5}	0.006	2	2	2	6.61×10^{-7}	0.092
ENSG00000150637.8	*CD226*	-3.90	9.70×10^{-5}	0.003	3	3	3	1.77×10^{-4}	0.081
ENSG00000146197.8	*SCUBE3*	3.88	1.02×10^{-4}	0.008	1	1	1	1.02×10^{-4}	0.154
ENSG00000105996.6	*HOXA2*	-3.84	1.23×10^{-4}	0.088	2	2	2	1.28×10^{-4}	0.421
ENSG00000140265.12	*ZSCAN29*	-3.82	1.31×10^{-4}	0.010	1	2	2	1.31×10^{-4}	0.150
ENSG00000099849.14	*RASSF7*	-3.82	1.34×10^{-4}	0.004	2	2	2	1.03×10^{-6}	0.112

© 2024 Fan J et al. *JAMA Network Open.*

ENSG00000164512.17	*ANKRD55*	3.80	1.47 x 10^{-4}	0.010	2	2	2	1.84 x 10^{-4}	0.137
ENSG00000079459.12	*FDFT1*	3.77	1.65 x 10^{-4}	0.028	2	2	2	4.55 x 10^{-4}	0.193
ENSG00000204084.12	*INPP5B*	3.75	1.78 x 10^{-4}	0.645	4	4	4	2.98 x 10^{-4}	0.505
ENSG00000010704.18	*HFE*	-3.74	1.84 x 10^{-4}	0.002	1	1	1	1.84 x 10^{-4}	0.067
ENSG00000125457.13	*MIF4GD*	3.74	1.87 x 10^{-4}	0.074	4	4	4	9.62 x 10^{-4}	0.300
ENSG00000159592.10	*GPBP1L1*	3.73	1.91 x 10^{-4}	0.000	1	1	1	1.91 x 10^{-4}	0.004
ENSG00000159596.6	*TMEM69*	3.73	1.91 x 10^{-4}	0.003	1	1	1	1.91 x 10^{-4}	0.261
ENSG00000104886.11	*PLEKHJ1*	3.73	1.91 x 10^{-4}	0.004	3	3	3	7.38 x 10^{-6}	0.223
ENSG00000073605.18	*GSDMB*	-3.68	2.35 x 10^{-4}	0.098	3	3	3	8.92 x 10^{-6}	0.339
ENSG00000113558.18	*SKP1*	3.66	2.51 x 10^{-4}	0.008	2	2	2	5.65 x 10^{-4}	0.219
ENSG00000172057.9	*ORMDL3*	-3.65	2.59 x 10^{-4}	0.137	2	2	2	8.04 x 10^{-6}	0.398
ENSG00000172890.11	*NADSYN1*	-3.64	2.68 x 10^{-4}	0.057	1	2	2	2.68 x 10^{-4}	0.392
ENSG00000172893.15	*DHCR7*	-3.62	2.90 x 10^{-4}	0.000	1	2	2	2.90 x 10^{-4}	0.033
ENSG00000108523.15	*RNF167*	-3.62	2.99 x 10^{-4}	0.006	2	2	2	3.11 x 10^{-3}	0.273
ENSG00000159314.11	*ARHGAP27*	3.61	3.01 x 10^{-4}	0.001	1	1	1	3.01 x 10^{-4}	0.044
ENSG00000120937.8	*NPPB*	-3.60	3.16 x 10^{-4}	0.001	1	1	1	3.16 x 10^{-4}	0.050
ENSG00000139899.10	*CBLN3*	-3.60	3.22 x 10^{-4}	0.748	3	3	3	2.23 x 10^{-4}	0.879
ENSG00000006837.11	*CDKL3*	-3.60	3.24 x 10^{-4}	0.004	1	2	2	3.24 x 10^{-4}	0.174
ENSG00000106004.4	*HOXA5*	-3.57	3.54 x 10^{-4}	0.012	2	2	2	3.24 x 10^{-4}	0.151
ENSG00000140829.11	*DHX38*	3.57	3.56 x 10^{-4}	0.003	1	1	1	3.56 x 10^{-4}	0.220
ENSG00000107625.12	*DDX50*	3.56	3.74 x 10^{-4}	0.002	2	2	2	1.05 x 10^{-3}	0.174
ENSG00000141744.3	*PNMT*	-3.56	3.76 x 10^{-4}	0.014	1	1	1	3.76 x 10^{-4}	0.170
ENSG00000277791.4	*PSMB3*	-3.55	3.83 x 10^{-4}	0.004	2	3	3	1.20 x 10^{-3}	0.087
ENSG00000105997.22	*HOXA3*	-3.55	3.84 x 10^{-4}	0.056	2	2	2	3.24 x 10^{-4}	0.211
ENSG00000175206.10	*NPPA*	-3.55	3.87 x 10^{-4}	0.008	1	1	1	3.87 x 10^{-4}	0.126
ENSG00000181458.10	*TMEM45A*	-3.52	4.27 x 10^{-4}	0.293	2	3	3	3.99 x 10^{-4}	0.540
ENSG00000141741.11	*MIEN1*	3.51	4.52 x 10^{-4}	0.000	1	1	1	4.52 x 10^{-4}	0.010
ENSG00000166963.12	*MAP1A*	3.50	4.64 x 10^{-4}	0.001	1	1	1	4.64 x 10^{-4}	0.038
ENSG00000197020.10	*ZNF100*	3.49	4.86 x 10^{-4}	0.173	2	2	2	3.75 x 10^{-4}	0.513
ENSG00000198453.12	*ZNF568*	-3.48	5.03 x 10^{-4}	0.007	1	1	1	5.03 x 10^{-4}	0.235
ENSG00000105991.7	*HOXA1*	-3.47	5.17 x 10^{-4}	0.067	1	1	1	5.17 x 10^{-4}	0.359
ENSG00000119048.7	*UBE2B*	3.44	5.72 x 10^{-4}	0.000	1	2	2	5.72 x 10^{-4}	0.002
ENSG00000198130.15	*HIBCH*	-3.44	5.73 x 10^{-4}	0.047	2	2	2	8.08 x 10^{-4}	0.311

© 2024 Fan J et al. *JAMA Network Open.*

ENSG00000198483.12	*ANKRD35*	3.43	5.95×10^{-4}	0.177	2	2	2	3.17×10^{-3}	0.496
ENSG00000229809.8	*ZNF688*	3.43	6.06×10^{-4}	0.001	1	1	1	6.06×10^{-4}	0.053
ENSG00000169951.9	*ZNF764*	3.43	6.07×10^{-4}	0.000	2	2	2	6.06×10^{-4}	0.043
ENSG00000140506.16	*LMAN1L*	-3.42	6.19×10^{-4}	0.002	1	1	1	6.19×10^{-4}	0.076
ENSG00000125485.17	*DDX31*	-3.42	6.29×10^{-4}	0.028	1	2	2	6.29×10^{-4}	0.290
ENSG00000142599.17	*RERE*	3.41	6.42×10^{-4}	0.021	1	1	1	6.42×10^{-4}	0.202
ENSG00000105063.18	*PPP6R1*	3.41	6.52×10^{-4}	0.003	1	1	1	6.52×10^{-4}	0.083
ENSG00000172594.12	*SMPDL3A*	-3.41	6.53×10^{-4}	0.016	3	3	3	9.36×10^{-3}	0.174
ENSG00000188227.12	*ZNF793*	-3.40	6.68×10^{-4}	0.038	1	1	1	6.68×10^{-4}	0.384

d. TWAS SSc

gene	gene_name	zscore	pvalue	var_g	n_snps_used	n_snps_in_cov	n_snps_in_model	best_gwas_p	largest_weight
ENSG00000168291.12	*PDHB*	-6.41	1.46×10^{-10}	0.020	2	2	2	6.31×10^{-9}	0.183
ENSG00000178802.17	*MPI*	-5.77	7.75×10^{-9}	0.058	2	2	2	1.31×10^{-9}	0.193
ENSG00000103653.16	*CSK*	5.65	1.63×10^{-8}	0.001	1	1	1	1.63×10^{-8}	0.049
ENSG00000128604.19	*IRF5*	5.49	4.05×10^{-8}	0.089	2	2	2	1.75×10^{-9}	0.387
ENSG00000136573.12	*BLK*	-5.12	3.03×10^{-7}	0.106	3	3	3	1.16×10^{-7}	0.243
ENSG00000161405.16	*IKZF3*	5.08	3.80×10^{-7}	0.003	1	1	1	3.80×10^{-7}	0.074
ENSG00000096996.15	*IL12RB1*	5.00	5.76×10^{-7}	0.034	2	2	2	3.79×10^{-8}	0.304
ENSG00000140497.16	*SCAMP2*	-4.94	7.71×10^{-7}	0.048	3	3	3	1.35×10^{-10}	0.179
ENSG00000144161.12	*ZC3H8*	4.83	1.36×10^{-6}	0.007	1	1	1	1.36×10^{-6}	0.149
ENSG00000081985.10	*IL12RB2*	4.83	1.37×10^{-6}	0.037	2	2	2	1.40×10^{-6}	0.351
ENSG00000168297.15	*PXK*	4.82	1.45×10^{-6}	0.010	3	3	3	2.36×10^{-6}	0.154
ENSG00000178741.11	*COX5A*	-4.53	5.94×10^{-6}	0.001	2	2	2	1.67×10^{-7}	0.034
ENSG00000213578.5	*CPLX3*	-4.46	8.27×10^{-6}	0.000	1	1	1	8.27×10^{-6}	0.001
ENSG00000110367.11	*DDX6*	4.36	1.33×10^{-5}	0.004	1	2	2	1.33×10^{-5}	0.099
ENSG00000064419.13	*TNPO3*	-4.20	2.67×10^{-5}	0.046	2	2	2	9.03×10^{-5}	0.339
ENSG00000073605.18	*GSDMB*	-4.12	3.84×10^{-5}	0.098	3	3	3	3.01×10^{-6}	0.339
ENSG00000100321.14	*SYNGR1*	4.08	4.44×10^{-5}	0.061	2	2	2	1.38×10^{-4}	0.427
ENSG00000154511.11	*FAM69A*	4.04	5.36×10^{-5}	0.010	1	1	1	5.36×10^{-5}	0.331
ENSG00000129422.14	*MTUS1*	-4.00	6.21×10^{-5}	0.162	2	2	2	6.70×10^{-4}	1.300
ENSG00000175110.11	*MRPS22*	3.86	1.12×10^{-4}	0.000	1	1	1	1.12×10^{-4}	0.006
ENSG00000140506.16	*LMAN1L*	-3.84	1.23×10^{-4}	0.002	1	1	1	1.23×10^{-4}	0.076

© 2024 Fan J et al. *JAMA Network Open.*

eTable 11. Genes having common significant associations with two or more rheumatic diseases.

Ensembl ID	Gene	Chr	Start(GRCh37)	End(GRCh37)	Associated Traits	Colocolized region?
ENSG00000134352	*IL6ST*	5	55230923	55290821	JIA, RA	No
ENSG00000181315	*ZNF322*	6	26636518	26659980	JIA, RA	No
ENSG00000168488	*ATXN2L*	16	28834356	28848558	JIA, RA	No
ENSG00000196296	*ATP2A1*	16	28889726	28915830	JIA, RA	No
ENSG00000260302	*RP11-973H7.1*	18	12774650	12775922	JIA, RA	Yes
ENSG00000144218	*AFF3*	2	100162323	100759201	JIA, RA	No
ENSG00000168071	*CCDC88B*	11	64107695	64125006	JIA, RA	No
ENSG00000161395	*PGAP3*	17	37827375	37853050	JIA, RA	Yes
ENSG00000125457	*MIF4GD*	17	73262309	73267308	JIA, SLE	No
ENSG00000204084	*INPP5B*	1	38326369	38412729	RA, SLE	No
ENSG00000141744	*PNMT*	17	37824234	37826728	RA, SLE	Yes
ENSG00000198369	*SPRED2*	2	65537985	65659771	RA, SLE	Yes
ENSG00000161847	*RAVER1*	19	10426888	10444316	RA, SLE	Yes
ENSG00000010704	*HFE*	6	26087509	26098571	RA, SLE	No
ENSG00000105991	*HOXA1*	7	27132612	27135615	RA, SLE	No
ENSG00000122484	*RPAP2*	1	92764522	92867613	RA, SLE	Yes
ENSG00000005020	*SKAP2*	7	26706681	27034858	RA, SLE	No
ENSG00000233429	*HOTAIRM1*	7	27135266	27139884	RA, SLE	No
ENSG00000105997	*HOXA3*	7	27145803	27192200	RA, SLE	No
ENSG00000110367	*DDX6*	11	118620034	118661858	RA, SSc	Yes
ENSG00000100321	*SYNGR1*	22	39745930	39781593	RA, SSc	Yes
ENSG00000069696	*DRD4*	11	637293	640706	SLE, SSc	Yes
ENSG00000168297	*PXK*	3	58318607	58411748	SLE, SSc	Yes
ENSG00000168291	*PDHB*	3	58413357	58419584	SLE, SSc	Yes

© 2024 Fan J et al. *JAMA Network Open.*

ENSG00000103653	*CSK*	15	75074398	75095539	SLE, SSc	Yes
ENSG00000140506	*LMAN1L*	15	75105057	75118099	SLE, SSc	Yes
ENSG00000105397	*TYK2*	19	10461209	10491352	JIA, RA, SLE	Yes
ENSG00000164512	*ANKRD55*	5	55395507	55529186	JIA, RA, SLE	Yes
ENSG00000118655	*DCLRE1B*	1	114447763	114456708	JIA, RA, SLE	Yes
ENSG00000161180	*CCDC116*	22	21987005	21991616	JIA, RA, SLE	Yes
ENSG00000137338	*PGBD1*	6	28249314	28270326	JIA, RA, SLE	No
ENSG00000141741	*MIEN1*	17	37884749	37887040	JIA, RA, SLE	Yes
ENSG00000096996	*IL12RB1*	19	18169805	18209754	JIA, SLE, SSc	Yes
ENSG00000136573	*BLK*	8	11351510	11422113	RA, SLE, SSc	No
ENSG00000081985	*IL12RB2*	1	67773047	67862583	RA, SLE, SSc	Yes
ENSG00000064419	*TNPO3*	7	128594948	128695198	RA, SLE, SSc	Yes
ENSG00000154319	*FAM167A*	8	11278972	11332224	RA, SLE, SSc	Yes
ENSG00000168811	*IL12A*	3	159706537	159713806	RA, SLE, SSc	Yes
ENSG00000161405	*IKZF3*	17	37921198	38020441	JIA, RA, SLE, SSc	Yes
ENSG00000073605	*GSDMB*	17	38060848	38076107	JIA, RA, SLE, SSc	Yes
ENSG00000172057	*ORMDL3*	17	38077294	38083854	JIA, RA, SLE, SSc	Yes
ENSG00000185651	*UBE2L3*	22	21903736	21978323	JIA, RA, SLE, SSc	Yes
ENSG00000128604	*IRF5*	7	128577666	128590089	JIA, RA, SLE, SSc	Yes

© 2024 Fan J et al. *JAMA Network Open.*

eFigure 14. Venn diagram of genes which are significantly associated with two or more autoimmune rheumatic diseases identified by SMR and TWAS (FDR ≤ 0.05).

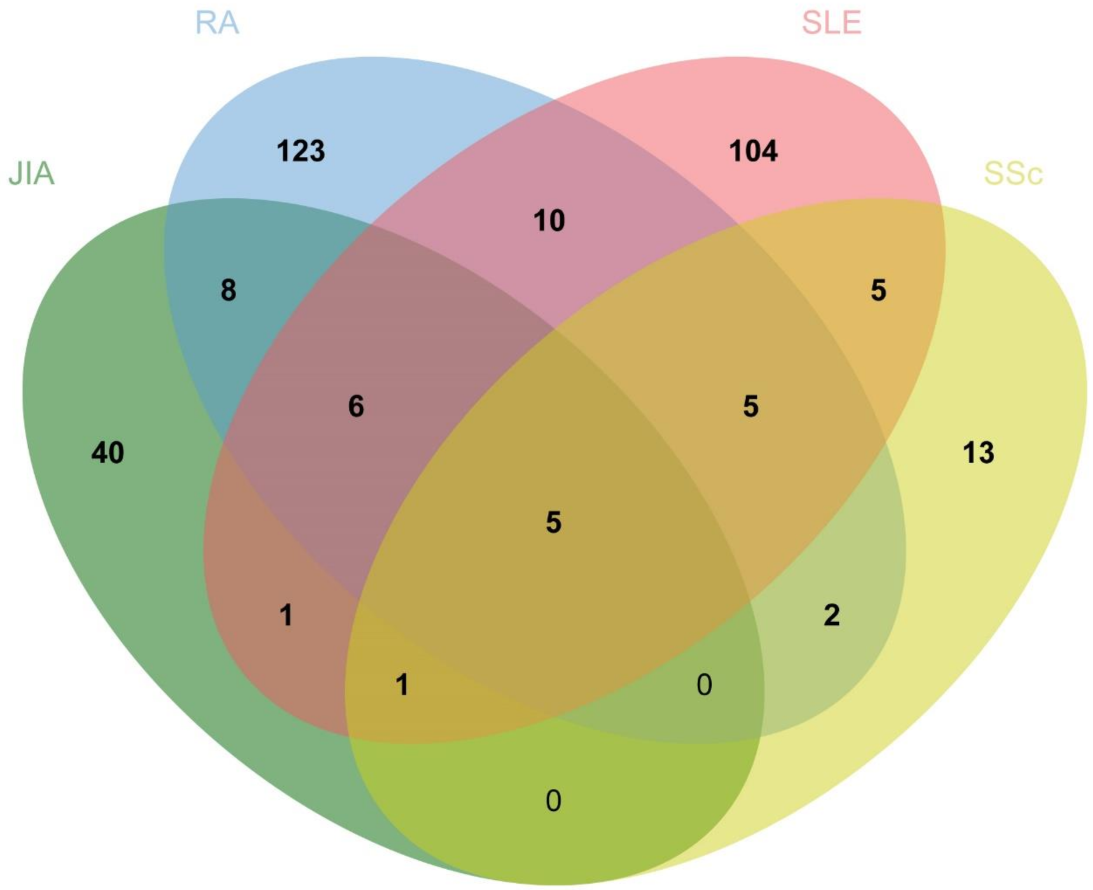

© 2024 Fan J et al. *JAMA Network Open.*

eTable 12. Drug-gene interactions among genes at the common loci associated with rheumatic diseases.

gene	interaction claim source	interaction types	drug claim name	drug claim primary name
CSK	GuideToPharmacology	inhibitor	223365983	RIVOCERANIB
CSK	TTD	/	936563-93-8	936563-93-8
CSK	PharmGKB	/	hydrochlorothiazide	hydrochlorothiazide
CSK	GuideToPharmacology	inhibitor	252827493	PP1
CSK	GuideToPharmacology	inhibitor	249565754	NG-25
CSK	TTD	/	BBI503	BBI503
CSK	DTC	inhibitor	HESPERADIN	HESPERADIN
DRD4	ChemblInteractions	antagonist	CHEMBL1201155	LOXAPINE SUCCINATE
DRD4	GuideToPharmacology	antagonist	135651092	(+)-SULPIRIDE
DRD4	GuideToPharmacology	agonist	135651480	DOPAMINE
DRD4	TEND	/	PROPIOMAZINE	PROPIOMAZINE
DRD4	ChemblInteractions	agonist	CHEMBL1303	ROTIGOTINE
DRD4	ChemblInteractions	antagonist	CHEMBL1764	LEVOMEPROMAZINE
DRD4	GuideToPharmacology	antagonist	223365892	PEROSPIRONE
DRD4	GuideToPharmacology	antagonist	135650345	HALOPERIDOL
DRD4	ChemblInteractions	antagonist	CHEMBL1113	AMOXAPINE
DRD4	TEND	/	ZIPRASIDONE	ZIPRASIDONE
DRD4	TdgClinicalTrial	/	ETILEVODOPA	ETILEVODOPA
DRD4	ChemblInteractions	antagonist	CHEMBL71	CHLORPROMAZINE
DRD4	ChemblInteractions	inverse agonist	CHEMBL2096643	HALOPERIDOL LACTATE
DRD4	ChemblInteractions	antagonist	CHEMBL715	OLANZAPINE
DRD4	TdgClinicalTrial	/	ROTIGOTINE	ROTIGOTINE
DRD4	TdgClinicalTrial	/	PROPIOMAZINE	PROPIOMAZINE
DRD4	TdgClinicalTrial	/	LU 02-750	LU 02-750
DRD4	GuideToPharmacology	antagonist	135651093	(-)-SULPIRIDE
DRD4	TEND	/	PROMAZINE	PROMAZINE
DRD4	ChemblInteractions	agonist	CHEMBL1200503	BROMOCRIPTINE MESYLATE
DRD4	ChemblInteractions	antagonist	CHEMBL479	THIORIDAZINE
DRD4	TdgClinicalTrial	/	ZICRONAPINE	ZICRONAPINE
DRD4	GuideToPharmacology	antagonist	178100332	[125I]L750667
DRD4	ChemblInteractions	antagonist	CHEMBL1710	TRIFLUOPERAZINE HYDROCHLORIDE
DRD4	GuideToPharmacology	agonist	135651054	FENOLDOPAM
DRD4	GuideToPharmacology	agonist	135650874	QUINPIROLE

© 2024 Fan J et al. *JAMA Network Open.*

DRD4	ChemblInteractions	agonist	CHEMBL542257	PRAMIPEXOLE DIHYDROCHLORIDE
DRD4	GuideToPharmacology	antagonist	135650833	PIRIBEDIL
DRD4	GuideToPharmacology	antagonist	135650533	LOXAPINE
DRD4	TdgClinicalTrial	/	PROMAZINE	PROMAZINE
DRD4	ChemblInteractions	antagonist	CHEMBL1201060	LOXAPINE HYDROCHLORIDE
DRD4	TEND	/	OLANZAPINE	OLANZAPINE
DRD4	GuideToPharmacology	antagonist	135650088	CHLORPROMAZINE
DRD4	TdgClinicalTrial	/	APOMORPHINE	APOMORPHINE
DRD4	GuideToPharmacology	antagonist	178100339	L741742
DRD4	GuideToPharmacology	antagonist	178100340	L745870
DRD4	ChemblInteractions	agonist	CHEMBL2146110	SARIZOTAN HYDROCHLORIDE
DRD4	ChemblInteractions	antagonist	CHEMBL1423	PIMOZIDE
DRD4	ChemblInteractions	antagonist	CHEMBL1108	DROPERIDOL
DRD4	TTD	/	NGD 94-1	NGD 94-1
DRD4	GuideToPharmacology	agonist	135652674	CABERGOLINE
DRD4	TdgClinicalTrial	/	CLOZAPINE	CLOZAPINE
DRD4	TdgClinicalTrial	/	LU AE04621	LU AE04621
DRD4	GuideToPharmacology	agonist	135650809	PERGOLIDE
DRD4	TTD	/	Phenyltoloxamine	Phenyltoloxamine
DRD4	TdgClinicalTrial	/	DEXPRAMIPEXOLE	DEXPRAMIPEXOLE
DRD4	GuideToPharmacology	antagonist	135650243	ETICLOPRIDE
DRD4	GuideToPharmacology	antagonist	178103853	PROCHLORPERAZINE
DRD4	GuideToPharmacology	antagonist	135650036	(+)-BUTACLAMOL
DRD4	GuideToPharmacology	agonist	135650929	ROTIGOTINE
DRD4	TdgClinicalTrial	/	ROPINIROLE	ROPINIROLE
DRD4	GuideToPharmacology	agonist	252166649	ABT-670
DRD4	ChemblInteractions	agonist	CHEMBL1616	APOMORPHINE HYDROCHLORIDE
DRD4	GuideToPharmacology	agonist	223366018	PF-592379
DRD4	TEND	/	THIETHYLPERAZINE	THIETHYLPERAZINE
DRD4	TTD	/	Intranasal apomorphine	Intranasal apomorphine
DRD4	GuideToPharmacology	agonist	135649950	APOMORPHINE
DRD4	TdgClinicalTrial	/	XP21279	XP21279
DRD4	GuideToPharmacology	antagonist	135651367	ZOTEPINE
DRD4	GuideToPharmacology	agonist	178100338	A412997
DRD4	ChemblInteractions	agonist	CHEMBL1200411	ROPINIROLE HYDROCHLORIDE

© 2024 Fan J et al. *JAMA Network Open.*

DRD4	TdgClinicalTrial	/	LEVODOPA	LEVODOPA
DRD4	TdgClinicalTrial	/	MELEVODOPA	MELEVODOPA
DRD4	GuideToPharmacology	antagonist	252166651	A-381393
DRD4	GuideToPharmacology	antagonist	315661291	BENPERIDOL
DRD4	TdgClinicalTrial	/	PARDOPRUNOX	PARDOPRUNOX
DRD4	GuideToPharmacology	antagonist	252166650	ML398
DRD4	TEND	/	PRAMIPEXOLE	PRAMIPEXOLE
DRD4	TEND	/	CLOZAPINE	CLOZAPINE
DRD4	TdgClinicalTrial	/	PRAMIPEXOLE	PRAMIPEXOLE
DRD4	TTD	/	CM-2395	CM-2395
DRD4	TEND	/	APOMORPHINE	APOMORPHINE
DRD4	TTD	/	PD-165167	PD-165167
DRD4	ChemblInteractions	agonist	CHEMBL2311030	ERGOLOID MESYLATES
DRD4	ChemblInteractions	inverse agonist	CHEMBL54	HALOPERIDOL
DRD4	ChemblInteractions	inverse agonist	CHEMBL1200986	HALOPERIDOL DECANOATE
DRD4	TTD	/	SPI-376	SPI-376
DRD4	TTD	/	PD-332991	PD-332991
DRD4	TTD	/	L-745,870	L-745,870
DRD4	TdgClinicalTrial	/	THIETHYLPERAZINE	THIETHYLPERAZINE
DRD4	GuideToPharmacology	antagonist	135651037	SERTINDOLE
DRD4	GuideToPharmacology	agonist	135650525	LISURIDE
DRD4	GuideToPharmacology	antagonist	135650115	CLOZAPINE
DRD4	TTD	/	U-99363E	U-99363E
DRD4	GuideToPharmacology	antagonist	135651279	TRIFLUOPERAZINE
DRD4	ChemblInteractions	antagonist	CHEMBL1200916	THIORIDAZINE HYDROCHLORIDE
DRD4	ChemblInteractions	antagonist	CHEMBL831	LOXAPINE
DRD4	ChemblInteractions	antagonist	CHEMBL1201741	OLANZAPINE PAMOATE
DRD4	TTD	/	Clozapine	Clozapine
DRD4	GuideToPharmacology	antagonist	178100334	[3H]NGD941
DRD4	GuideToPharmacology	antagonist	135651246	TERGURIDE
DRD4	TdgClinicalTrial	/	OLANZAPINE	OLANZAPINE
DRD4	ChemblInteractions	agonist	CHEMBL1275	PERGOLIDE MESYLATE
DRD4	TdgClinicalTrial	/	ASENAPINE	ASENAPINE
DRD4	ChemblInteractions	antagonist	CHEMBL1200469	PROMAZINE HYDROCHLORIDE
DRD4	GuideToPharmacology	antagonist	178102406	U101958
DRD4	GuideToPharmacology	antagonist	135650707	NEMONAPRIDE

© 2024 Fan J et al. *JAMA Network Open.*

DRD4	GuideToPharmacology	agonist	135651321	WAY-100635
DRD4	GuideToPharmacology	antagonist	135651064	SPIPERONE
DRD4	GuideToPharmacology	agonist	135650930	ROXINDOLE
DRD4	ChemblInteractions	antagonist	CHEMBL1713	CHLORPROMAZINE HYDROCHLORIDE
DRD4	GuideToPharmacology	antagonist	135650842	SONEPIPRAZOLE
DRD4	TTD	/	RP5063	RP5063
DRD4	GuideToPharmacology	antagonist	178100337	[3H]SPIPERONE
DRD4	GuideToPharmacology	antagonist	135650026	BROMOCRIPTINE
IL12A	TdgClinicalTrial	/	BRIAKINUMAB	BRIAKINUMAB
IL12A	TTD	/	Ustekinumab	Ustekinumab
IL12A	TdgClinicalTrial	/	AD-RTS-IL-12	AD-RTS-IL-12
IL12A	PharmGKB	/	ustekinumab	ustekinumab
IL12A	ChemblInteractions	inhibitor	CHEMBL1201835	USTEKINUMAB
IL12A	TdgClinicalTrial	/	VIRULIZIN	VIRULIZIN
IL12A	TTD	/	STA-5326	STA-5326
IL12A	TdgClinicalTrial	/	DC-RTS-IL-12	DC-RTS-IL-12
IL12A	ChemblInteractions	inhibitor	CHEMBL1742995	BRIAKINUMAB
IL12A	ClearityFoundationClinicalTrial	/	CID 446414	CID 446414
IL12A	PharmGKB	/	mycophenolate mofetil	mycophenolate mofetil
IL12A	TdgClinicalTrial	/	DNA IL-12	DNA IL-12
IL12A	TdgClinicalTrial	/	HUMANIZED SMART ANTI-IL-12 ANTIBODY	HUMANIZED SMART ANTI-IL-12 ANTIBODY
IL12A	TTD	/	Mirikizumab	Mirikizumab
IL12A	FDA	/	Ustekinumab	Ustekinumab
IL12RB2	TdgClinicalTrial	/	INCELL-1001+ AD-1001	INCELL-1001+ AD-1001
IL12RB2	TdgClinicalTrial	/	AS1409	AS1409
IL12RB2	TdgClinicalTrial	/	DENILEUKIN DIFTITOX	DENILEUKIN DIFTITOX
PDHB	TdgClinicalTrial	/	CPI-613	CPI-613
PDHB	TdgClinicalTrial	/	DEXLIPOTAM	DEXLIPOTAM
PNMT	NCI	/	NERVE GROWTH FACTOR	NERVE GROWTH FACTOR
PNMT	GuideToPharmacology	inhibitor	178103244	LY134046
TYK2	GuideToPharmacology	inhibitor	178102342	FEDRATINIB
TYK2	GuideToPharmacology	inhibitor	315661321	UPADACITINIB
TYK2	TTD	/	PMID27774822-Compound-Figure9Example15	PMID27774822-Compound-Figure9Example15
TYK2	TTD	/	Pyrrolo-pyridone derivative 3	Pyrrolo-pyridone derivative 3

TYK2	ChemblInteractions	inhibitor	CHEMBL2103743	TOFACITINIB CITRATE
TYK2	GuideToPharmacology	inhibitor	405067336	AZD4205
TYK2	GuideToPharmacology	inhibitor	385612218	IZENCITINIB
TYK2	TTD	/	Aminopyrimidine derivative 5	Aminopyrimidine derivative 5
TYK2	GuideToPharmacology	inhibitor	178102315	RUXOLITINIB
TYK2	TTD	/	PMID27774822-Compound-Figure3CompoundI-165	PMID27774822-Compound-Figure3CompoundI-165
TYK2	TTD	/	Pyrazolopyridine derivative 3	Pyrazolopyridine derivative 3
TYK2	DTC	inhibitor	HESPERADIN	HESPERADIN
TYK2	GuideToPharmacology	inhibitor	348353655	OCLACITINIB
TYK2	DTC	/	SP-600125	SP-600125
TYK2	DTC	/	CYC-116	CYC-116
TYK2	DTC	/	TOFACITINIB	TOFACITINIB
TYK2	DTC	/	TAE-684	TAE-684
TYK2	TTD	/	Pyrrolo[2,3-d]pyrimidine derivative 7	Pyrrolo[2,3-d]pyrimidine derivative 7
TYK2	TTD	/	Bis-aminopyrimidine derivative 5	Bis-aminopyrimidine derivative 5
TYK2	TTD	/	Aminopyridine derivative 1	Aminopyridine derivative 1
TYK2	DTC	/	JNJ-7706621	JNJ-7706621
TYK2	TTD	/	Pyrrolo[2,3-d]pyrimidine derivative 6	Pyrrolo[2,3-d]pyrimidine derivative 6
TYK2	NCI	/	IL-12	IL-12
TYK2	DTC	/	ILORASERTIB	ILORASERTIB
TYK2	NCI	/	IFN	IFN
TYK2	TTD	/	Bis-aminopyrimidine derivative 1	Bis-aminopyrimidine derivative 1
TYK2	TTD	/	PF-06826647	PF-06826647
TYK2	TTD	/	Aminotriazolopyridine derivative 1	Aminotriazolopyridine derivative 1
TYK2	TTD	/	Pyrazolo[4,3-c]pyridine derivative 2	Pyrazolo[4,3-c]pyridine derivative 2
TYK2	TTD	/	PMID27774824-Compound-Figure11Example1up	PMID27774824-Compound-Figure11Example1up
TYK2	TTD	/	Imidazo[4,5-c]pyridine derivative 1	Imidazo[4,5-c]pyridine derivative 1
TYK2	TTD	/	Pyrazolopyridine derivative 5	Pyrazolopyridine derivative 5

© 2024 Fan J et al. *JAMA Network Open.*

TYK2	TTD	/	Benzimidazole derivative 7	Benzimidazole derivative 7
TYK2	TTD	/	Thiazolopyridine derivative 1	Thiazolopyridine derivative 1
TYK2	GuideToPharmacology	inhibitor	348353656	SOLCITINIB
TYK2	TTD	/	Tricyclic heterocycle derivative 5	Tricyclic heterocycle derivative 5
TYK2	TTD	/	Pyrrolo[2,3-d]pyrimidine derivative 8	Pyrrolo[2,3-d]pyrimidine derivative 8
TYK2	GuideToPharmacology	inhibitor	381118867	JAK3 INHIBITOR 34
TYK2	GuideToPharmacology	inhibitor	381118866	JAK3 INHIBITOR 32
TYK2	TTD	/	Tricyclic pyrrolopyridine compound 1	Tricyclic pyrrolopyridine compound 1
TYK2	ChemblInteractions	inhibitor	CHEMBL3137308	PEFICITINIB
TYK2	DTC	/	CENISERTIB	CENISERTIB
TYK2	GuideToPharmacology	inhibitor	374883879	COMPOUND 29 [MOSLIN ET AL., 2017]
TYK2	GuideToPharmacology	inhibitor	315661180	TG02
TYK2	TTD	/	PMID27774824-Compound-Figure6Example12	PMID27774824-Compound-Figure6Example12
TYK2	GuideToPharmacology	inhibitor	405560501	PF-956980
TYK2	TTD	/	Imidazo[4,5-c]pyridine derivative 2	Imidazo[4,5-c]pyridine derivative 2
TYK2	NCI	/	IL-2	IL-2
TYK2	DTC	/	TAMATINIB	TAMATINIB
TYK2	GuideToPharmacology	inhibitor	178102614	JAK INHIBITOR I
TYK2	TTD	/	PMID27774824-Compound-Figure9Example2down	PMID27774824-Compound-Figure9Example2down
TYK2	TTD	/	Pyrrole derivative 7	Pyrrole derivative 7
TYK2	DTC	/	PF-562271	PF-562271
TYK2	GuideToPharmacology	inhibitor	223366123	BARICITINIB
TYK2	DTC	/	GO-6976	GO-6976
TYK2	GuideToPharmacology	inhibitor	385612219	BREPOCITINIB
TYK2	TTD	/	BMS-986165	BMS-986165
TYK2	TTD	/	PF-06700841	PF-06700841
TYK2	GuideToPharmacology	inhibitor	249565637	BMS-911543
TYK2	TTD	/	PMID27774822-Compound-Figure11Example5	PMID27774822-Compound-Figure11Example5
TYK2	GuideToPharmacology	inhibitor	252166527	PEFICITINIB

© 2024 Fan J et al. *JAMA Network Open.*

TYK2	TTD	/	PMID27774824-Compound-Figure9Example2up	PMID27774824-Compound-Figure9Example2up
TYK2	DTC	/	RG-1530	RG-1530
TYK2	TTD	/	Tricyclic heterocycle derivative 1	Tricyclic heterocycle derivative 1
TYK2	DTC	/	TOZASERTIB	TOZASERTIB
TYK2	TTD	/	N-methylmethanesulfonamide derivative 1	N-methylmethanesulfonamide derivative 1
TYK2	TTD	/	Bis-aminopyrimidine derivative 3	Bis-aminopyrimidine derivative 3
TYK2	TALC	inhibitor	AT9283	AT9283
TYK2	TTD	/	Tricyclic compound 11	Tricyclic compound 11
TYK2	GuideToPharmacology	allosteric modulator	385612192	BMS-986165
TYK2	GuideToPharmacology	inhibitor	340590249	DELGOCITINIB
TYK2	TTD	/	Bis-aminopyrimidine derivative 4	Bis-aminopyrimidine derivative 4
TYK2	TTD	/	Bis-aminopyrimidine derivative 2	Bis-aminopyrimidine derivative 2
TYK2	ChemblInteractions	inhibitor	CHEMBL3545284	Cerdulatinib
TYK2	TTD	/	Aminooxazole carboxamide derivative 1	Aminooxazole carboxamide derivative 1
TYK2	GuideToPharmacology	inhibitor	387065609	LASW1393
TYK2	TTD	/	TYrosine kinase 2 inhibitor	TYrosine kinase 2 inhibitor
TYK2	GuideToPharmacology	inhibitor	310264738	CERDULATINIB
TYK2	GuideToPharmacology	inhibitor	381118858	JAK INHIBITOR 17B
TYK2	GuideToPharmacology	inhibitor	404859078	EXAMPLE 1 [WO2018112379A1]
TYK2	DTC	/	DOVITINIB	DOVITINIB
TYK2	GuideToPharmacology	inhibitor	354702275	SAR-20347
TYK2	GuideToPharmacology	inhibitor	381118857	JAK INHIBITOR 20A
TYK2	GuideToPharmacology	inhibitor	223366170	ILGINATINIB
TYK2	TTD	/	PMID27774824-Compound-Figure3Example18	PMID27774824-Compound-Figure3Example18
TYK2	GuideToPharmacology	inhibitor	329968373	PF-06263276
TYK2	GuideToPharmacology	inhibitor	249565596	FILGOTINIB
TYK2	TTD	/	Imidazopyridine derivative 3	Imidazopyridine derivative 3
TYK2	GuideToPharmacology	inhibitor	178102304	TOFACITINIB

eTable 13. MR results for blood proteins obtaining a significant MR P value (after Bonferroni correction) for association with rheumatic disorders. The ones, which also showed significant associations with the rheumatic disorders at the mRNA level, are highlighted in bold.

Protein	Druggability Tier	IV	EA	BETA (SE)	OR	95% CI	P-value	R2	F-statistics	Disease	Resource (PMID)
IL27	Tier 3A	rs181209	T	-0.71 (0.15)	0.49	0.36-0.66	2.22×10^{-6}	0.01	45.96	JIA	29875488
**ICAM5**	/	rs2278442	A	0.45 (0.11)	1.58	1.28-1.94	1.80×10^{-5}	0.03	98.36	JIA	29875488
LMAN2L	/	rs2229169; rs2271893	G; A	0.39 (0.09)	1.48	1.24-1.75	7.90×10^{-6}	0.04	128.76	JIA	29875488
**ICAM5**	/	rs2278442	A	0.37 (0.06)	1.45	1.28-1.65	3.73×10^{-9}	0.03	98.36	RA	29875488
**FCRL3**	Tier 3A	rs7528684	G	0.14 (0.03)	1.15	1.09-1.21	1.05×10^{-7}	0.13	440.69	RA	29875488
**FCRL3**	Tier 3A	rs7522061	C	0.09 (0.02)	1.10	1.05-1.14	5.77×10^{-6}	0.22	391.74	RA	28240269
FCGR2B	Tier 1	rs7551957	C	-0.10 (0.02)	0.91	0.87-0.94	1.66×10^{-7}	0.39	223.03	RA	28240269
IL6R	Tier 1	rs4129267	T	-0.06 (0.01)	0.94	0.92-0.97	1.04×10^{-5}	0.70	2321.67	RA	28240269
**PAM**	Tier 2	rs17335068, rs257309	T; G	0.13 (0.03)	1.14	1.07-1.21	1.94×10^{-5}	0.13	475.60	RA	29875488
PPP3CA	Tier 1	rs17266357	C	-0.41 (0.08)	0.66	0.57-0.78	5.47×10^{-7}	0.06	62.92	SLE	28240269
CCL22	Tier 3B	rs223883	G	-0.71 (0.16)	0.49	0.35-0.68	1.48×10^{-5}	0.02	96.32	SLE	35078996
**DEF6**	/	rs3800401	A	-1.04 (0.24)	0.35	0.22-0.57	2.54×10^{-5}	0.01	18.68	SLE	30072576
TIMP4	Tier 3B	rs394945	T	0.34 (0.08)	1.40	1.21-1.63	7.74×10^{-6}	0.04	142.89	SSc	30072576

© 2024 Fan J et al. *JAMA Network Open.*

eFigure 15. Construction of genetic risk score of progression to RA for JIA patients. (A) The rank range of the RA index SNP from each genomic region associated with both JIA and RA. The different percentiles of ranks for each SNP indicated with different darkness of orange color. The index SNPs in the regions identified by both local genetic correlation and colocalization analyses are indicated with asterisks. The "Median" column indicates the median rank achieved by each marker across all 1000 simulations. (B) The histogram depicting the distribution of genetic risk scores for developing adult arthritis among each patient. Overlaid was a fitted curve showing the normal distribution of these polygenic risk scores when considering the additive effects of the established risk variants for rheumatoid arthritis.

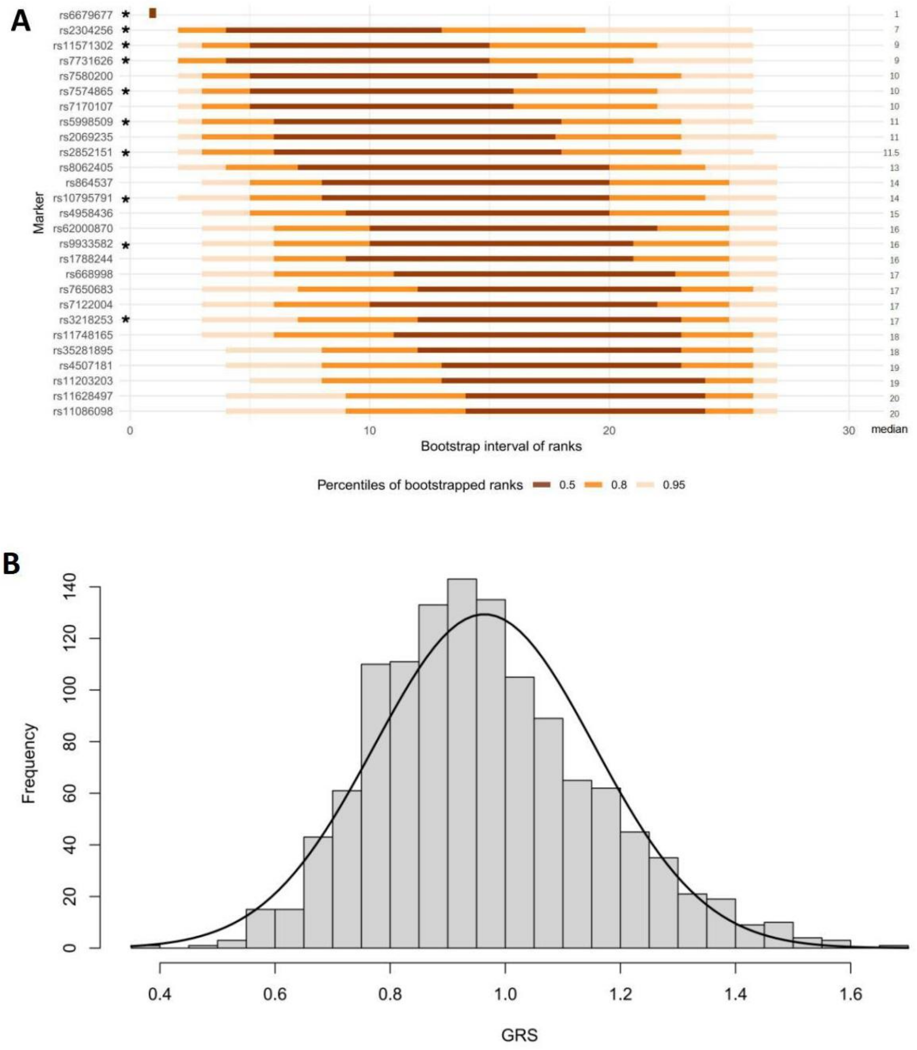

© 2024 Fan J et al. *JAMA Network Open.*

eFigure 16. Tissue expression analysis of 30 general tissue types obtained from Genotype-Tissue Expression version 8 (GTEx v8) using MAGMA gene-property test for 4 autoimmune rheumatic diseases.

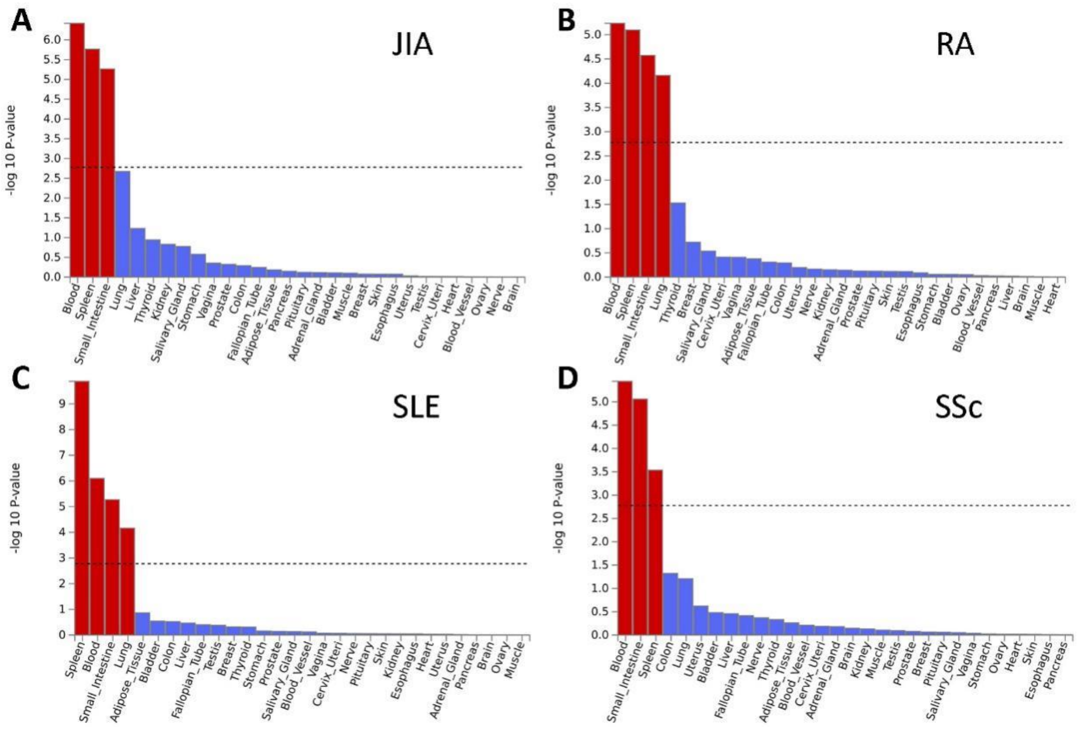

© 2024 Fan J et al. *JAMA Network Open.*

eFigure 17. The UMAPs showing the associations of cell types in blood with genetic risk of rheumatic disorders. The upper panel shows UMAP embedding of 27 cell types in blood annotated by original data. The lower panel shows UMAP embedding of cell types in blood colored by scDRS disease score calculated from GWAS summary statistics of four autoimmune rheumatic disorders.

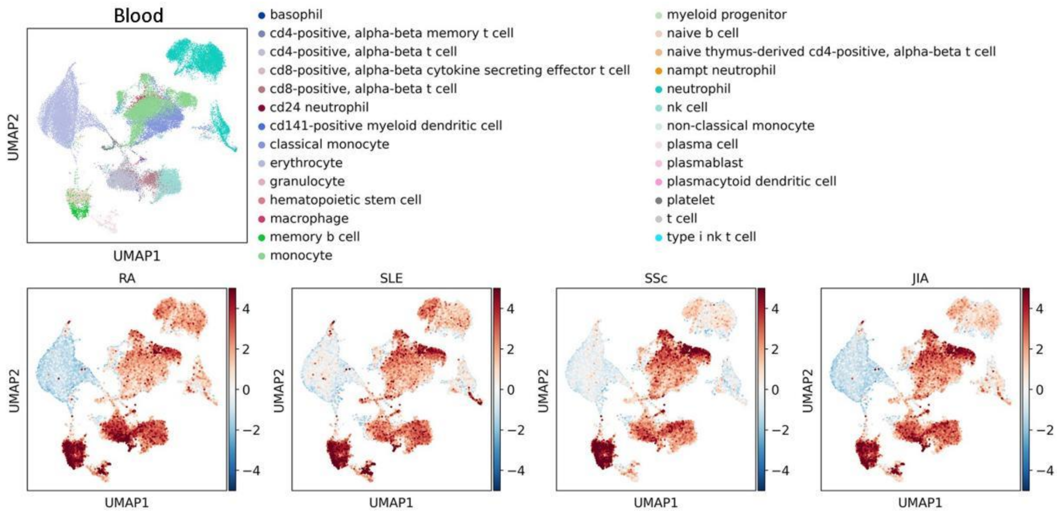

© 2024 Fan J et al. *JAMA Network Open.*

eFigure 18. Associations of cell types in lung with genetic risk of rheumatic disorders. The top panel shows UMAP embedding of 38 cell types in lung annotated by original data. The middle panel shows UMAP embedding of cell types in blood colored by scDRS disease score calculated from GWAS summary statistics of four autoimmune rheumatic disorders. The bottom panel displays the heatmaps depicting each cell type-disease association for each of the four traits, respectively. The darkness of heatmap colors denotes the proportion of significantly associated cells for each disease. Squares represent significant cell type-disease associations with multiple testing adjustment (FDR < 0.05), and cross symbols mean significant heterogeneity across individual cells within a particular cell type for association with the disease (FDR < 0.05)

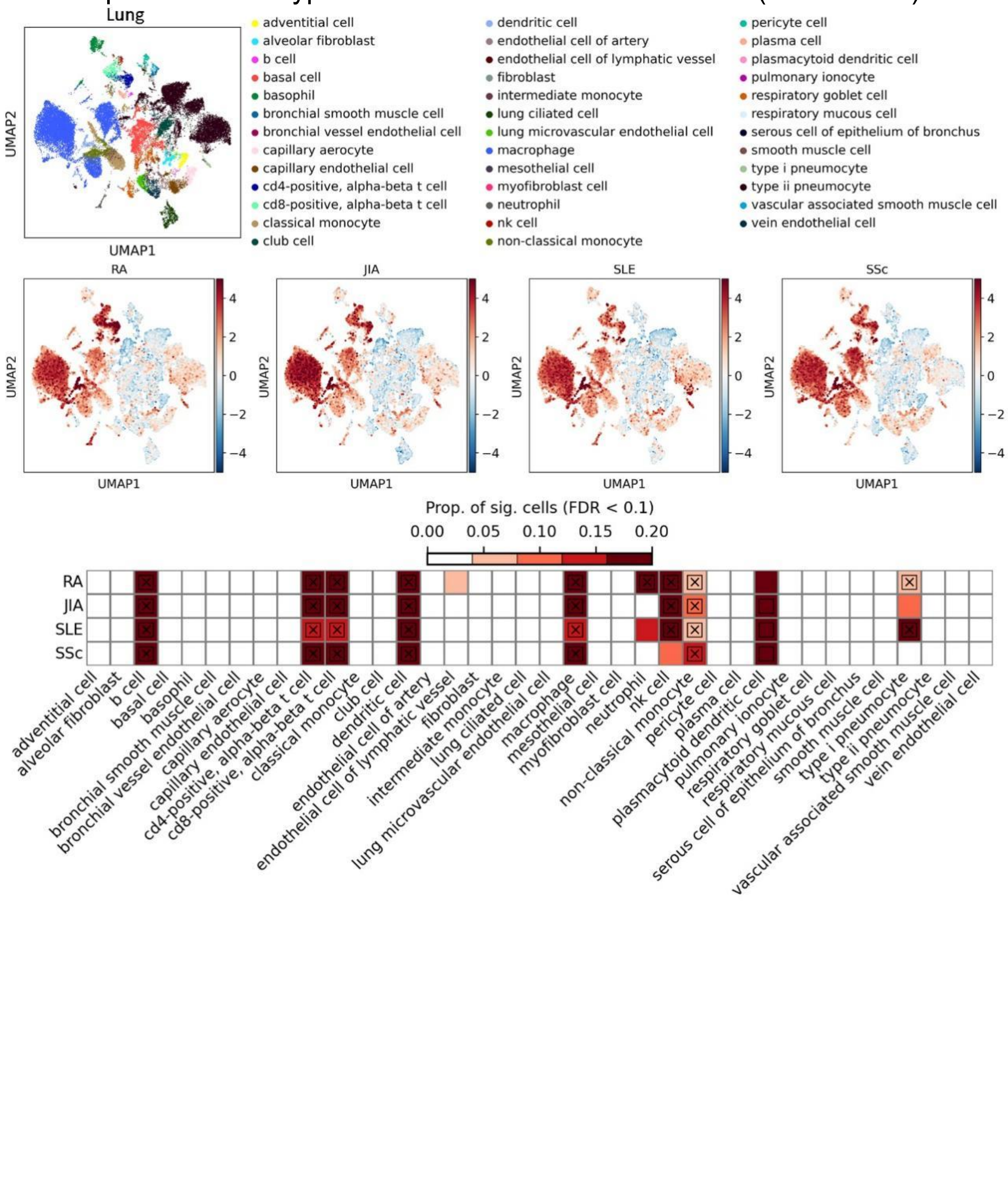

© 2024 Fan J et al. *JAMA Network Open.*

eFigure 19. Associations of cell types in spleen with genetic risk of rheumatic disorders.The top panel shows UMAP embedding of 24 cell types in spleen annotated by original data. The middle panel shows UMAP embedding of cell types in blood colored by scDRS disease score calculated from GWAS summary statistics of four autoimmune rheumatic disorders. The bottom panel displays the heatmaps depicting each cell type-disease association for each of the four traits, respectively. The darkness of heatmap colors denotes the proportion of significantly associated cells for each disease. Squares represent significant cell type-disease associations with multiple testing adjustment (FDR < 0.05), and cross symbols mean significant heterogeneity across individual cells within a particular cell type for association with the disease (FDR < 0.05).

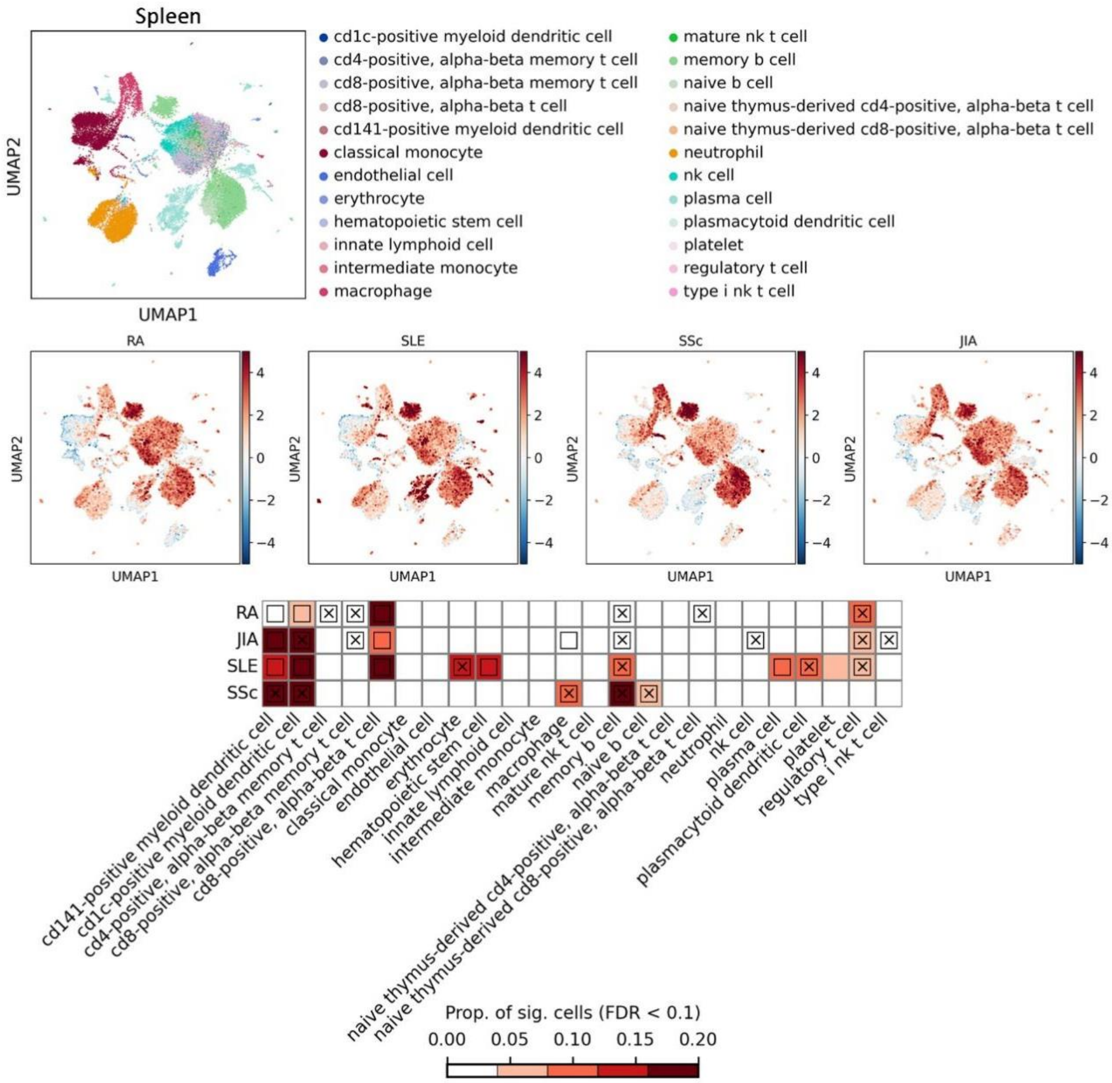

© 2024 Fan J et al. *JAMA Network Open.*

eFigure 20. Associations of cell types in small intestine with genetic risk of autoimmune rheumatic disorders. The top panel shows UMAP embedding of 21 cell types in small intestine annotated by original data. The middle panel shows UMAP embedding of cell types in blood colored by scDRS disease score calculated from GWAS summary statistics of four autoimmune rheumatic disorders. The bottom panel displays the heatmaps depicting each cell type-disease association for each of the four traits, respectively. The darkness of heatmap colors denotes the proportion of significantly associated cells for each disease. Squares represent significant cell type-disease associations with multiple testing adjustment (FDR < 0.05), and cross symbols mean significant heterogeneity across individual cells within a particular cell type for association with the disease (FDR < 0.05).

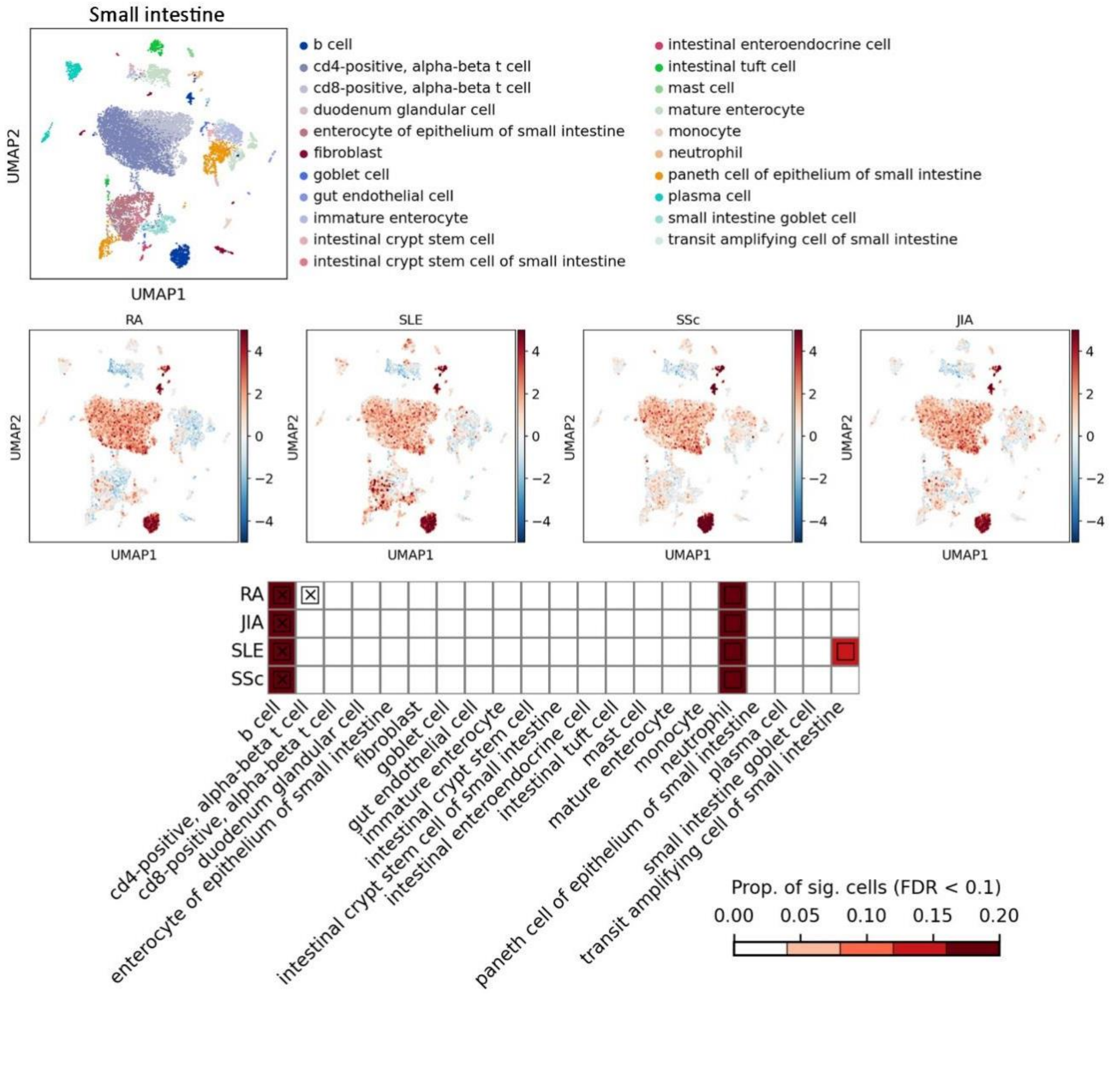

© 2024 Fan J et al. *JAMA Network Open.*

eREFERENCES

1. Lopez-Isac E, Smith SL, Marion MC, et al. Combined genetic analysis of juvenile idiopathic arthritis clinical subtypes identifies novel risk loci, target genes and key regulatory mechanisms. *Ann Rheum Dis.* 2021;80(3):321-328.
2. Fan J, Li X, Yang J, et al. Revealing Novel Genomic Insights and Therapeutic Targets for Juvenile Idiopathic Arthritis through Omics. *Rheumatology (Oxford).* 2024.
3. Okada Y, Wu D, Trynka G, et al. Genetics of rheumatoid arthritis contributes to biology and drug discovery. *Nature.* 2014;506(7488):376-381.
4. Bentham J, Morris DL, Graham DSC, et al. Genetic association analyses implicate aberrant regulation of innate and adaptive immunity genes in the pathogenesis of systemic lupus erythematosus. *Nature genetics.* 2015;47(12):1457-1464.
5. López-Isac E, Acosta-Herrera M, Kerick M, et al. GWAS for systemic sclerosis identifies multiple risk loci and highlights fibrotic and vasculopathy pathways. *Nature communications.* 2019;10(1):4955.
6. Bulik-Sullivan BK, Loh PR, Finucane HK, et al. LD Score regression distinguishes confounding from polygenicity in genome-wide association studies. *Nature genetics.* 2015;47(3):291-295.
7. Turley P, Walters RK, Maghzian O, et al. Multi-trait analysis of genome-wide association summary statistics using MTAG. *Nature genetics.* 2018;50(2):229-237.
8. Bhattacharjee S, Rajaraman P, Jacobs KB, et al. A subset-based approach improves power and interpretation for the combined analysis of genetic association studies of heterogeneous traits. *American journal of human genetics.* 2012;90(5):821-835.
9. Purcell S, Neale B, Todd-Brown K, et al. PLINK: a tool set for whole-genome association and population-based linkage analyses. *American journal of human genetics.* 2007;81(3):559-575.
10. Yang J, Ferreira T, Morris AP, et al. Conditional and joint multiple-SNP analysis of GWAS summary statistics identifies additional variants influencing complex traits. *Nature genetics.* 2012;44(4):369-375, S361-363.
11. Watanabe K, Taskesen E, van Bochoven A, Posthuma D. Functional mapping and annotation of genetic associations with FUMA. *Nature communications.* 2017;8(1):1826.
12. Guo H, Li JJ, Lu Q, Hou L. Detecting local genetic correlations with scan statistics. *Nature communications.* 2021;12(1):2033.
13. Giambartolomei C, Vukcevic D, Schadt EE, et al. Bayesian test for colocalisation between pairs of genetic association studies using summary statistics. *PLoS genetics.* 2014;10(5):e1004383.
14. Bakshi A, Zhu Z, Vinkhuyzen AA, et al. Fast set-based association analysis using summary data from GWAS identifies novel gene loci for human complex traits. *Sci Rep.* 2016;6:32894.
15. Yu G, Wang LG, Han Y, He QY. clusterProfiler: an R package for comparing biological themes among gene clusters. *OMICS.* 2012;16(5):284-287.
16. Consortium GT. The GTEx Consortium atlas of genetic regulatory effects across human tissues. *Science.* 2020;369(6509):1318-1330.
17. Vosa U, Claringbould A, Westra HJ, et al. Large-scale cis- and trans-eQTL analyses identify thousands of genetic loci and polygenic scores that regulate blood gene expression. *Nature genetics.* 2021;53(9):1300-1310.
18. Lloyd-Jones LR, Holloway A, McRae A, et al. The Genetic Architecture of Gene Expression in Peripheral Blood. *American journal of human genetics.* 2017;100(2):371.
19. Barbeira AN, Dickinson SP, Bonazzola R, et al. Exploring the phenotypic consequences of tissue specific gene expression variation inferred from GWAS summary statistics. *Nature communications.*

© 2024 Fan J et al. *JAMA Network Open.*

2018;9(1):1825.
20. Sun BB, Maranville JC, Peters JE, et al. Genomic atlas of the human plasma proteome. *Nature.* 2018;558(7708):73-79.
21. Emilsson V, Ilkov M, Lamb JR, et al. Co-regulatory networks of human serum proteins link genetics to disease. *Science.* 2018;361(6404):769-773.
22. Gudjonsson A, Gudmundsdottir V, Axelsson GT, et al. A genome-wide association study of serum proteins reveals shared loci with common diseases. *Nature communications.* 2022;13(1):480.
23. Suhre K, Arnold M, Bhagwat AM, et al. Connecting genetic risk to disease end points through the human blood plasma proteome. *Nature communications.* 2017;8:14357.
24. Burgess S, Thompson SG. Bias in causal estimates from Mendelian randomization studies with weak instruments. *Stat Med.* 2011;30(11):1312-1323.
25. Bowden J, Spiller W, Del Greco MF, et al. Improving the visualization, interpretation and analysis of two-sample summary data Mendelian randomization via the Radial plot and Radial regression. *Int J Epidemiol.* 2018;47(6):2100.
26. Zheng J, Baird D, Borges MC, et al. Recent Developments in Mendelian Randomization Studies. *Curr Epidemiol Rep.* 2017;4(4):330-345.
27. Verbanck M, Chen CY, Neale B, Do R. Detection of widespread horizontal pleiotropy in causal relationships inferred from Mendelian randomization between complex traits and diseases. *Nature genetics.* 2018;50(5):693-698.
28. Bowden J, Davey Smith G, Haycock PC, Burgess S. Consistent Estimation in Mendelian Randomization with Some Invalid Instruments Using a Weighted Median Estimator. *Genet Epidemiol.* 2016;40(4):304-314.
29. Zhang MJ, Hou K, Dey KK, et al. Polygenic enrichment distinguishes disease associations of individual cells in single-cell RNA-seq data. *Nature genetics.* 2022;54(10):1572-1580.
30. Tabula Sapiens C, Jones RC, Karkanias J, et al. The Tabula Sapiens: A multiple-organ, single-cell transcriptomic atlas of humans. *Science.* 2022;376(6594):eabl4896.
31. Steyerberg EW, Harrell FE, Jr., Borsboom GJ, Eijkemans MJ, Vergouwe Y, Habbema JD. Internal validation of predictive models: efficiency of some procedures for logistic regression analysis. *J Clin Epidemiol.* 2001;54(8):774-781.
32. Yang J, Lee SH, Goddard ME, Visscher PM. GCTA: a tool for genome-wide complex trait analysis. *American journal of human genetics.* 2011;88(1):76-82.
33. Igo RP, Jr., Kinzy TG, Cooke Bailey JN. Genetic Risk Scores. *Curr Protoc Hum Genet.* 2019;104(1):e95.

© 2024 Fan J et al. *JAMA Network Open.*
