# Supplementary material for: Genetic Association of Juvenile Idiopathic Arthritis With Adult Rheumatic Disease
Source: JAMA Netw Open. 2024 Dec 27;7(12):e2451341. doi: 10.1001/jamanetworkopen.2024.51341 (PMC11681380; doi:10.1001/jamanetworkopen.2024.51341)
Supplement: Supplement 2. — Data Sharing Statement [file jamanetwopen-e2451341-s002.pdf]

Data Sharing Statement

Fan. Genetic Association of Juvenile Idiopathic Arthritis With Adult Rheumatic Disease. *JAMA Netw Open*. Published December 17, 2024. doi:10.1001/jamanetworkopen.2024.51341

Data
Data available: Yes
Data types: Other (please specify)
Additional Information: The GWAS summary statistics
How to access data: The GWAS summary statistics are available upon reasonable request to Dr. Hakon Hakonarson (hakonarson@chop.edu).
When available: With publication

Supporting Documents
Document types: Statistical/analytic code
How to access documents: The request should be sent to Dr. Jin Li (jli01@tmu.edu.cn).
When available: With publication

Additional Information
Who can access the data: Researchers whose proposed use of the data has been approved.
Types of analyses: Academic research.
Mechanisms of data availability: with a signed data access agreement.
